# Supplementary material for: CRISPR/Cas9-mediated gene knockin in the hydroid Hydractinia symbiolongicarpus
Source: BMC Genomics. 2018 Sep 3;19:649. doi: 10.1186/s12864-018-5032-z (PMC6122657; doi:10.1186/s12864-018-5032-z)
Supplement: Supplementary file 1 — Figure S1. Pedigree of the colonies used to generate zygotes for microinjection. Figure S2. Effects and outcomes of microinjection. Figure S3. eGFP+ vs. eGFPdim larvae. Figure S4. FLAG immunolocalization controls. Table S1. Oligonucleotide sequences used in this study. (PDF 2121 kb) [file 12864_2018_5032_MOESM1_ESM.pdf]

SUPPLEMENTARY INFORMATION

CRISPR/Cas9-mediated gene knockin in the hydroid *Hydractinia symbiolongicarpus*

Steven M. Sanders[1,2], Zhiwei Ma[1,2], Julia M. Hughes[1,2], Brooke M. Riscoe[1,2], Gregory A. Gibson[3], Alan M. Watson[3], Hakima Flici[4], Uri Frank[4], Christine E. Schnitzler[5], Andreas D. Baxevanis[6], and Matthew L. Nicotra[1,2,7]*

[1] Thomas E. Starzl Transplantation Institute, Department of Surgery, University of Pittsburgh, Pittsburgh, PA, USA

[2] Pittsburgh Center for Evolutionary Biology and Medicine, University of Pittsburgh, Pittsburgh, PA, USA

[3] Center for Biological Imaging and Department of Cell Biology, University of Pittsburgh, Pittsburgh, PA, USA

[4] Centre for Chromosome Biology, School of Natural Sciences, National University of Ireland, Galway, Ireland

[5] Whitney Laboratory for Marine Bioscience, and Department of Biology, University of Florida, St. Augustine, FL, USA

[6] Computational and Statistical Genomics Branch, National Human Genome Research Institute, National Institutes of Health, Bethesda, MD, USA

[7] Department of Immunology, University of Pittsburgh, Pittsburgh, PA, USA

* Corresponding author

Email: matthew.nicotra@pitt.edu (MLN)

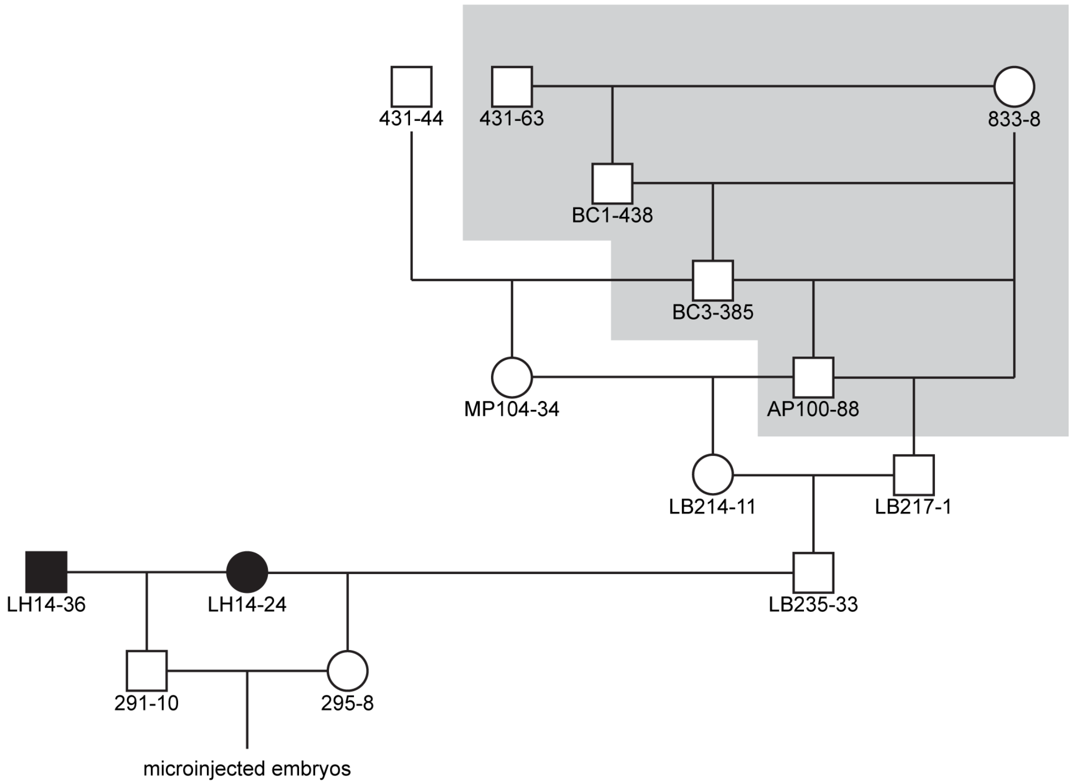

Figure S1. Pedigree of the colonies used to generate zygotes for microinjection.

Field-collected colonies are denoted with black symbols. Colony 291-10 is the offspring of two colonies collected from Lighthouse Point, New Haven, CT in 2014. Colony 295-8 is the offspring of a field collected colony and a laboratory strain, 235-33. The pedigree of colony 235-33 can be recreated by concatenating previously published pedigrees (shaded area) (Powell *et al.* 2007; Cadavid *et al*. 2004). Colony AP100-88 is from the mapping population in Powell *et al.* 2007. Colony 431-44 is from the mapping population in Cadavid *et al*. 2004.

Cadavid LF, Powell AE, Nicotra ML, Moreno M, Buss LW. An invertebrate histocompatibility complex. Genetics. 2004;167:357–65.

Powell AE, Nicotra ML, Moreno MA, Lakkis FG, Dellaporta SL, Buss LW. Differential effect of allorecognition loci on phenotype in Hydractinia symbiolongicarpus (Cnidaria: Hydrozoa). Genetics. 2007;177:2101–7.

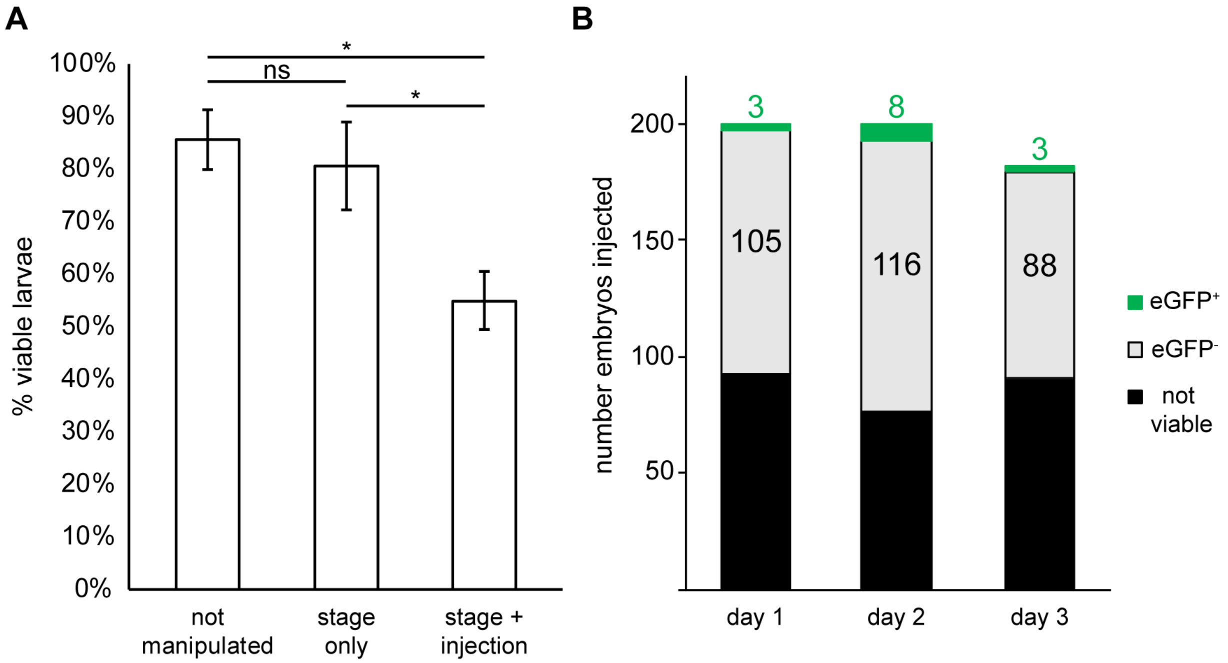

Figure S2. Effects and outcomes of microinjection.

(A) Fertilized embryos were collected and either left in a dish (not manipulated), placed on the microscope stage during microinjections, but not injected (stage only), or injected with the microinjection cocktail (stage + injection). Embryos were assessed at 48 hpf for viability. N = 3 independent experiments. * = $p<0.01$ by Student's t-test. ns = not significant. (B) Raw data from three days of microinjections. Numbers of viable eGFP positive and negative larvae are indicated on or above the bar graph.

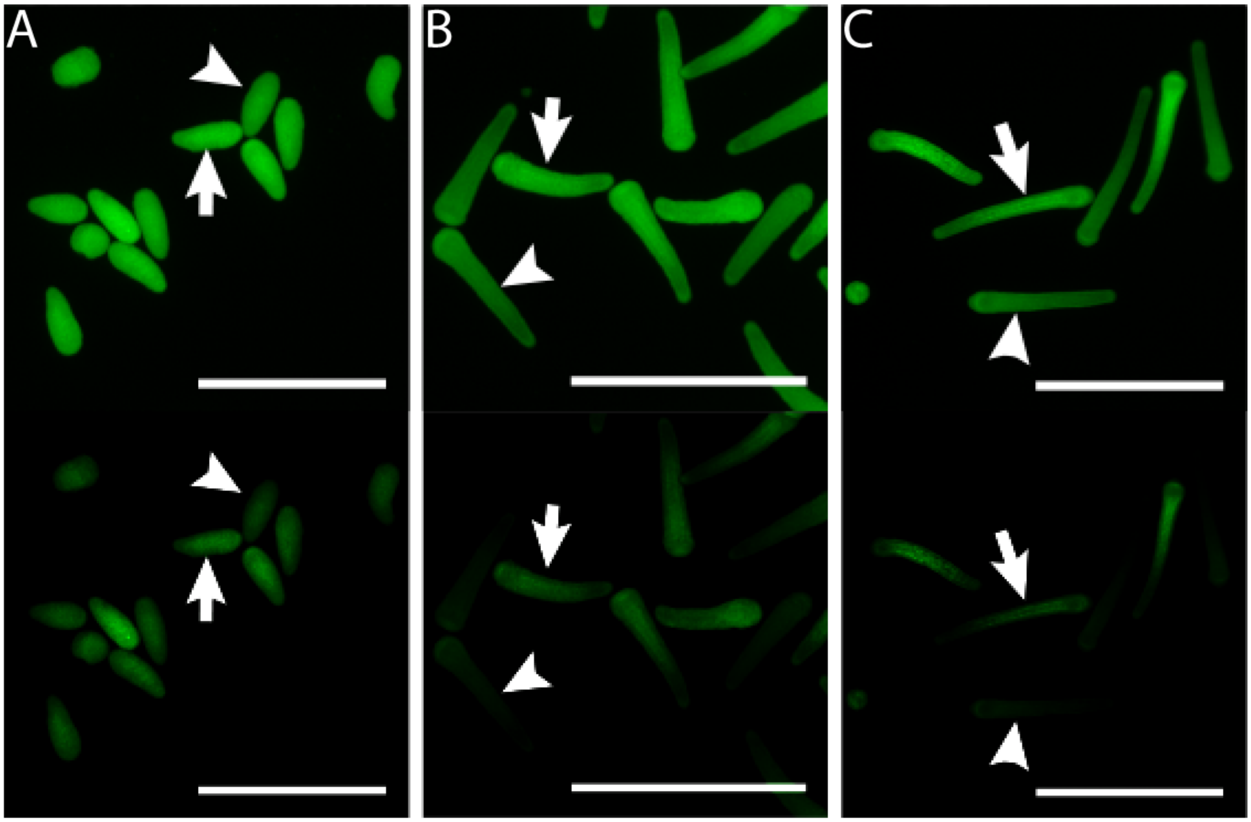

Figure S3. eGFP^{+} vs. eGFP^{dim} larvae.

Figure 2D-F images with gamma correction to increase the contrast between eGFP^{+} and eGFP^{dim} larvae. Original image on top row and gamma corrected version on bottom. (A) 24 hpf embryos from the backcross of 347-10 to 291-10, beginning to show slightly dimmer eGFP signal in some larvae (arrowhead) compared to others (arrow). (B) 72 hpf embryos from the same cross. eGFP^{+} (arrow) and eGFP^{dim} (arrowhead) larvae are now evident. (C) 168 hr-old embryos. The distinction between eGFP^{+} (arrow) and eGFP^{dim} (arrowhead) larvae is even more substantial. All scale bars = 1 mm.

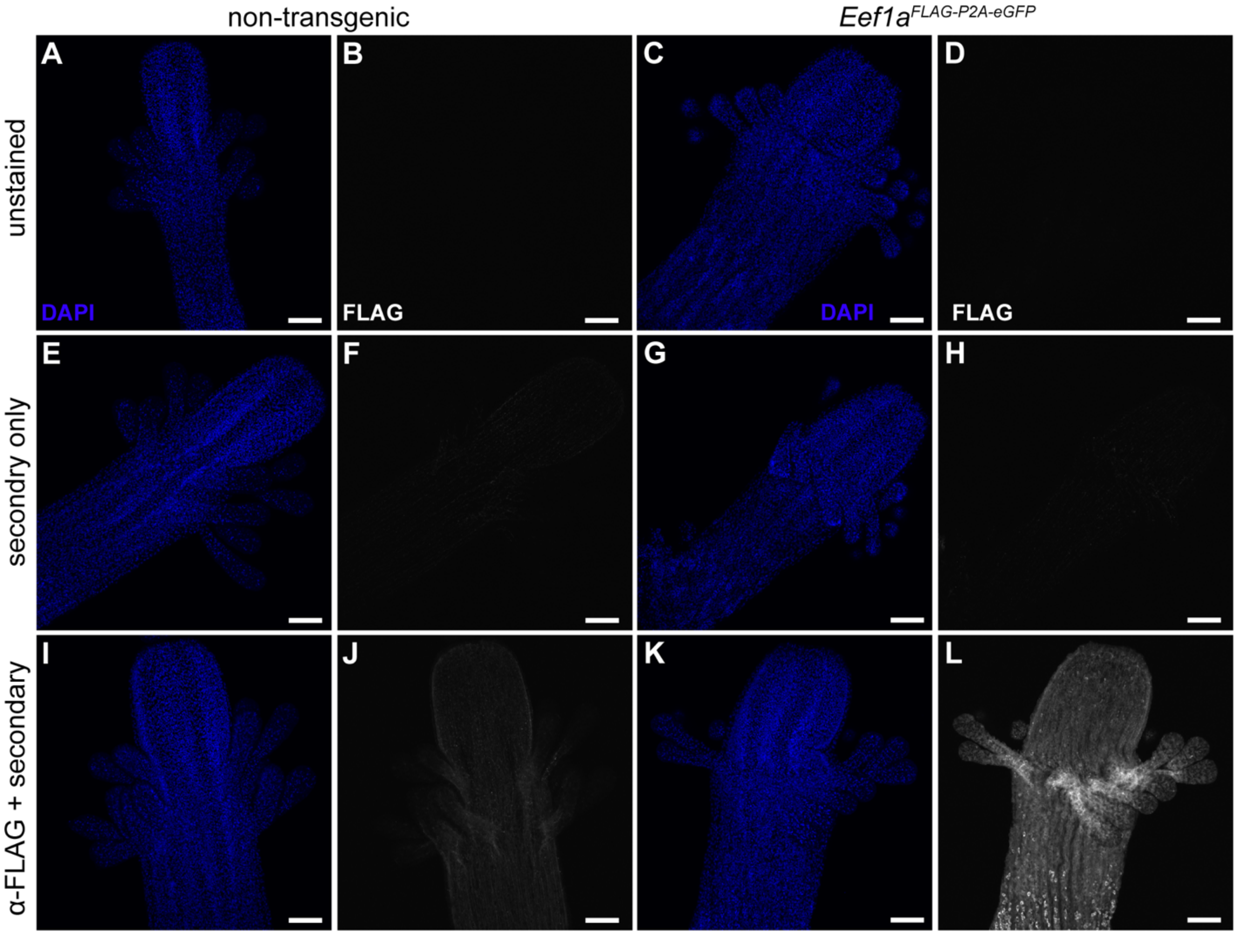

Figure S4. FLAG immunolocalization controls

Gastrozooids were fixed, permeabilized, stained with the indicated combination of antibodies, and counterstained with DAPI. (A-D) Gastrozooids from nontransgenic or transgenic gastrozooids stained with neither primary nor secondary antibody. (E-H) Gastrozooids stained only with the Alexa 647 conjugated goat anti-mouse secondary antibody. (I-L) Gastrozooids stained with mouse anti-FLAG primary antibody followed by Alexa 647 goat anti-mouse secondary. No background signal was detected on the Alexa 647 channel in unstained non-transgenic or transgenic polyps (B,D). Very faint background signal was detected from Alexa 647 in the secondary-only control, although this was equivalent between non-transgenic and transgenic gastrozooids (F and H, respectively). Increased background was detected in the non-transgenic gastrozooids stained with primary and secondary antibodies (J), but this was lower than that observed in transgenic polyps (L). Scale bars = 100 μm in all panels.

Table S1. Oligonucleotide sequences used in this study.

**Oligo ID**	**Sequence (5’→3’)**	Comment
sgRNA_847	CATAGGTCACTTCTTTTTTC	Figure 1
Pr845	GTAGAAACTGGTGTATTGTCCCCT	Figure 3 & 7
Pr875	TTTTCGTTGGGATCTTTCGAAAGG	Figure 3
Pr876	CCAGGTCCAATGAGTAAAGGAGAA	Figure 3
Pr877	CAATAATAAACGCAACATCGCTGC	Figure 3 & 7
Pr846	ACTTCACATTTGGTTTACGCTACG	Figure 7
Pr899	TTGATGCCTCCCAACCCATC	Figure 7
Pr900	CCTGTCCAGTTACCAGGCTAC	Figure 7
Pr901	GCGAATGGAGGGCTCAATGA	Figure 7
Pr996	AGGAACACAAACAGCCAAATTGAA	Figure 7
Pr998	AATGACACACTCGCTGACGT	Figure 7
Pr1000	TTGAAGGGGAGGGTGAAGGA	Figure 7
Pr1045	GATCAGAAGGCCGTCATCATTTG	Figure 7
Pr1046	ATATGTCAGGATTTTGCGCCATG	Figure 7
Pr1047	AGGCCGTCATCATTTGTTTCTTG	Figure 7
Pr1048	AGGGATAAAGCTAAAAGTGCCCA	Figure 7
