# Supplementary material for: CRISPR/Cas9-mediated gene knockin in the hydroid Hydractinia symbiolongicarpus
Source: BMC Genomics. 2018 Sep 3;19:649. doi: 10.1186/s12864-018-5032-z (PMC6122657; doi:10.1186/s12864-018-5032-z)
Supplement: Supplementary file 2 — Alignment of cloned Eef1a sequences from female colony 295–8. Two PCR amplicons (sized 2.4 kb and 2.0 kb) from the genomic region encoding the 3′ end of Eef1a in colony 295–8 were cloned, sequenced (3 clones each), and aligned. This revealed two alleles, with the major differences being a 52 bp and a 313 bp indel. Differences are shown in red boxes. Sequence features are shown on and above the top sequence of the alignment. Green, Eef1a mRNA as determined by alignments to 291–10 transcripts. Yellow, Eef1a coding sequence. (PDF 4523 kb) [file 12864_2018_5032_MOESM2_ESM.pdf]

```
                                    20                  40                  60
Eef1a_gDNA_295-8_2.4kb_allele_clone2 GTAGAAACTGGTGTATTGTCCCCTGGTATGATCGTTACATTTGCCCCTGCCAATGTAACC 60
Eef1a_gDNA_295-8_2.4kb_allele_clone1 GTAGAAACTGGTGTATTGTCCCCTGGTATGATCGTTACATTTGCCCCTGCCAATGTAACC 60
EF1a_gDNA_295-8_2.4kb_allele_clone3 GTAGAAACTGGTGTATTGTCCCCTGGTATGATCGTTACATTTGCCCCTGCCAATGTAACC 60
EF1a_gDNA_295-8_2kb_allele_clone2 GTAGAAACTGGTGTATTGTCCCCTGGTATGATCGTTACATTTGCCCCTGCCAATGTAACC 60
EF1a_gDNA_295-8_2kb_allele_clone3 GTAGAAACTGGTGTATTGTCCCCTGGTATGATCGTTACATTTGCCCCTGCCAATGTAACC 60
Eef1a_gDNA_295-8_2kb_allele_clone5 GCAGAAACTGGTGTATTGTCCCCTGGTATGATCGTTACATTTGCCCCTGCCAATGTAACC 60
```

```
                                    80                  100                 120
Eef1a_gDNA_295-8_2.4kb_allele_clone2 ACTGAGGTCAAATCTGTAGAAATGCACCACGAATCCTTAACTGAAGCACATCCAGGTGAT 120
Eef1a_gDNA_295-8_2.4kb_allele_clone1 ACTGAGGTCAAATCTGTAGAAATGCACCACGAATCCTTAACTGAAGCACATCCAGGTGAT 120
EF1a_gDNA_295-8_2.4kb_allele_clone3 ACTGAGGTCAAATCTGTAGAAATGCACCACGAATCCTTAACTGAAGCACATCCAGGTGAT 120
EF1a_gDNA_295-8_2kb_allele_clone2 ACTGAGGTCAAATCTGTAGAAATGCACCACGAATCCTTAACTGAAGCACATCCAGGTGAT 120
EF1a_gDNA_295-8_2kb_allele_clone3 ACTGAGGTCAAATCTGTAGAAATGCACCACGAATCCTTAACTGAAGCACATCCAGGTGAT 120
Eef1a_gDNA_295-8_2kb_allele_clone5 ACTGAGGTCAAATCTGTAGAAATGCACCACGAATCCTTAACTGAAGCACATCCAGGTGAT 120
```

```
                                    140                 160                 180
Eef1a_gDNA_295-8_2.4kb_allele_clone2 AATGTTGGTTTTAATGTCAAAAATGTGTCTATCAAGGATATCAAACGTGGTATGGTTGCG 180
Eef1a_gDNA_295-8_2.4kb_allele_clone1 AATGTTGGTTTTAATGTCAAAAATGTGTCTATCAAGGATATCAAACGTGGTATGGTTGCG 180
EF1a_gDNA_295-8_2.4kb_allele_clone3 AATGTTGGTTTTAATGTCAAAAATGTGTCTATCAAGGATATCAAACGTGGTATGGTTGCG 180
EF1a_gDNA_295-8_2kb_allele_clone2 AATGTTGGTTTTAATGTCAAAAATGTGTCTATCAAGGATATCAAACGTGGTATGGTTGCG 180
EF1a_gDNA_295-8_2kb_allele_clone3 AATGTTGGTTTTAATGTCAAAAATGTGTCTATCAAGGATATCAAACGTGGTATGGTTGCG 180
Eef1a_gDNA_295-8_2kb_allele_clone5 AATGTTGGTTTTAATGTCAAAAATGTGTCTATCAAGGATATCAAACGTGGTATGGTTGCG 180
```

```
                                    200                 220                 240
Eef1a_gDNA_295-8_2.4kb_allele_clone2 TCTGACAGCAAAAATGATCCAGCCAAAGAAGCCAAAACCTTTTATGCACAGGTATTCTTT 240
Eef1a_gDNA_295-8_2.4kb_allele_clone1 TCTGACAGCAAAAATGATCCAGCCAAAGAAGCCAAAACCTTTTATGCACAGGTATTCTTT 240
EF1a_gDNA_295-8_2.4kb_allele_clone3 TCTGACAGCAAAAATGATCCAGCCAAAGAAGCCAAAACCTTTTATGCACAGGTATTCTTT 240
EF1a_gDNA_295-8_2kb_allele_clone2 TCTGACAGCAAAAATGATCCAGCCAAAGAAGCCAAAACCTTTTATGCACAGGTATTCTTT 240
EF1a_gDNA_295-8_2kb_allele_clone3 TCTGACAGCAAAAATGATCCAGCCAAAGAAGCCAAAACCTTTTATGCACAGGTATTCTTT 240
Eef1a_gDNA_295-8_2kb_allele_clone5 TCTGACAGCAAAAATGATCCAGCCAAAGAAGCCAAAACCTTTTATGCACAGGTATTCTTT 240
```

```
                                    260                 280                 300
Eef1a_gDNA_295-8_2.4kb_allele_clone2 AATTTTTGTACTTAACTTATCAGTTTGTATAAGCGTGTTGGCCGTGGCTTGCCACTTGCT 300
Eef1a_gDNA_295-8_2.4kb_allele_clone1 AATTTTTGTACTTAACTTATCAGTTTGTATAAGCGTGTTGGCCGTGGCTTGCCACTTGCT 300
EF1a_gDNA_295-8_2.4kb_allele_clone3 AATTTTTGTACTTAACTTATCAGTTTGTATAAGCGTGTTGGCCGTGGCTTGCCACTTGCT 300
EF1a_gDNA_295-8_2kb_allele_clone2 AATTTTTGTACTTAACTTATCAGTTTGTATAAGCGTGTTGGCCGTGGCTTGCCACTTGCT 300
EF1a_gDNA_295-8_2kb_allele_clone3 AATTTTTGTACTTAACTTATCAGTTTGTATAAGCGTGTTGGCCGTGGCTTGCCACTTGCT 300
Eef1a_gDNA_295-8_2kb_allele_clone5 AATTTTTGTACTTAACTTATCAGTTTGTATAAGCGTGTTGGCCGTGGCTTGCCACTTGCT 300
```

```
                                    320                 340                 360
Eef1a_gDNA_295-8_2.4kb_allele_clone2 TAGCATGATAAATCAATCAACTTGACATTCCATCAATTTTGTTCAACAGAAACAGGACCT 360
Eef1a_gDNA_295-8_2.4kb_allele_clone1 TAGCATGATAAATCAATCAACTTGACATTCCATCAATTTTGTTCAACAGAAACAGGACCT 360
EF1a_gDNA_295-8_2.4kb_allele_clone3 TAGCATGATAAATCAATCAACTTGACATTCCATCAATTTTGTTCAACAGAAACAGGACCT 360
EF1a_gDNA_295-8_2kb_allele_clone2 TAGCATGATAAATCAATCAACTTGACATTCCATCAATTTTGTTCAACAGAAACAGGACCT 360
EF1a_gDNA_295-8_2kb_allele_clone3 TAGCATGATAAATCAATCAACTTGACATTCCATCAATTTTGTTCAACAGAAGCAGGACCT 360
Eef1a_gDNA_295-8_2kb_allele_clone5 TAGCATGATAAATCAATCAACTTGACATTCCATCAATTTTGTTCAACAGAAACAGGACCT 360
```

```
                                    380                 400                 420
Eef1a_gDNA_295-8_2.4kb_allele_clone2 TTAAATAGCAAACAAAATATTGAAATAAAACTGTATGATTTTGTCACTATCGTTTATGTG 420
Eef1a_gDNA_295-8_2.4kb_allele_clone1 TTAAATAGCAAACAAAATATTGAAATAAAACTGTATGATTTTGTCACTATCGTTTATGTG 420
EF1a_gDNA_295-8_2.4kb_allele_clone3 TTAAATAGCAAACAAAATATTGAAATAAAACTGTATGATTTTGTCACTATCGTTTATGTG 420
EF1a_gDNA_295-8_2kb_allele_clone2 TTAAATAGCAAACAAAATATTGAAATAAAACTGTATGATTTTGTCACTATCGTTTATGTG 420
EF1a_gDNA_295-8_2kb_allele_clone3 TTAAATAGCAAACAAAATATTGAAATAAAACTGTATGATTTTGTCACTATCGTTTATGTG 420
Eef1a_gDNA_295-8_2kb_allele_clone5 TTAAATAGCAAACAAAATATTGAAATAAAACTGTATGATTTTGTCACTATCGTTTATGTG 420
```

Supporting Figure S2

```
                                                                          440                 460                 480
Eef1a_gDNA_295-8_2.4kb_allele_clone2  CCCACCATGAAAATTTTTACAAAGAAGTGGAACTGTTTGAAAGTTCATTAGCAAGAACAAA 480
Eef1a_gDNA_295-8_2.4kb_allele_clone1  CCCACCATGAAAATTTTTACAAAGAAGTGGAACTGTTTGAAAGTTCATTAGCAAGAACAAA 480
EF1a_gDNA_295-8_2.4kb_allele_clone3   CCCACCATGAAAATTTTTACAAAGAAGTGGAACTGTTTGAAAGTTCATTAGCAAGAACAAA 480
EF1a_gDNA_295-8_2kb_allele_clone2     CCCACCATGAAAATTTTTACAAAGAAGTGGAACTGTTTGAAAGTTCATTAGCAAGAACAAA 480
EF1a_gDNA_295-8_2kb_allele_clone3     CCCACCATGAAAATTTTTACAAAGAAGTGGAACTGTTTGAAAGTTCATTAGCAAGAACAAA 480
Eef1a_gDNA_295-8_2kb_allele_clone5    CCCACCATGAAAATTTTTACAAAGAAGTGGAACTGTTTGAAAGTTCATTAGCAAGAACAAA 480

                                                                          500                 520                 540
Eef1a_gDNA_295-8_2.4kb_allele_clone2  TCCTAGAAATGTTAGATTTGTGTCAAGAATTTC--AAGATTTTTGTTTCTTGGCAAGTTT 538
Eef1a_gDNA_295-8_2.4kb_allele_clone1  TCCTAGAAATGTTAGATTTGTGTCAAGAATTTC--AAGATTTTTGTTTCTTGGCAAGTTT 538
EF1a_gDNA_295-8_2.4kb_allele_clone3   TCCTAGAAATGTTAGATTTGTGTCAAGAATTTC--AAGATTTTTGTTTCTTGGCAAGTTT 538
EF1a_gDNA_295-8_2kb_allele_clone2     TCCTAGAAATGTTAGATTTGTGTCAAGAATTTCTTAAGATTTTTGTTTCTTGGCAAGTAT 540
EF1a_gDNA_295-8_2kb_allele_clone3     TCCTAGAAATGTTAGATTTGTGTCAAGAATTTCTTAAGATTTTTGTTTCTTGGCAAGTAT 540
Eef1a_gDNA_295-8_2kb_allele_clone5    TCCTAGAAATGTTAGATTTGTGTCAAGAATTTCTTAAGATTTTTGTTTCTTGGCAAGTAT 540

                                                                          560                 580                 600
Eef1a_gDNA_295-8_2.4kb_allele_clone2  TTAGCACTTGCTTAGGGAACTATTTAAAATATTAATTTTTATTGAATAAAGAACCAACCT 598
Eef1a_gDNA_295-8_2.4kb_allele_clone1  TTAGCACTTGCTTAGGGAACTATTTAAAATATTAATTTTTATTGAATAAAGAACCAACCT 598
EF1a_gDNA_295-8_2.4kb_allele_clone3   TTAGCACTTGCTTAGGGAACTATTTAAAATATTAATTTTTATTGAATAAAGAACCAACCT 598
EF1a_gDNA_295-8_2kb_allele_clone2     TTGGCACTTGCTTAGGGAACTATTTAAAATATTAATTTTTATTGAATAAAGAACCAACCT 600
EF1a_gDNA_295-8_2kb_allele_clone3     TTGGCACTTGCTTAGGGAACTATTTAAAATATTAATTTTTATTGAATAAAGAACCAACCT 600
Eef1a_gDNA_295-8_2kb_allele_clone5    TTGGCACTTGCTTAGGGAACTATTTAAAATATTAATTTTTATTGAATAAAGAACCAACCT 600

                                                                          620                 640                 660
Eef1a_gDNA_295-8_2.4kb_allele_clone2  GTTTGACTTTTATTAGTAATAAAGCTAAAATTATGGCCTAAATCATAATTTATTATGTGA 658
Eef1a_gDNA_295-8_2.4kb_allele_clone1  GTTTGACTTTTATTAGTAATAAAGCTAAAATTATGGCCTAAATCATAATTTATTATGTGA 658
EF1a_gDNA_295-8_2.4kb_allele_clone3   GTTTGACTTTTATTAGTAATAAAGCTAAAATTATGGCCTAAATCATAATTTATTATGTGA 658
EF1a_gDNA_295-8_2kb_allele_clone2     GTTTGACTTTTATTAGTAATAAAGCTAAAATTATGGCCTAAATCATAATTTATTATGTGA 660
EF1a_gDNA_295-8_2kb_allele_clone3     GTTTGACTTTTATTAGTAATAAAGCTAAAATTATGGCCTAAATCATAATTTATTATGTGA 660
Eef1a_gDNA_295-8_2kb_allele_clone5    GTTTGACTTTTATTAGTAATAAAGCTAAAATTATGGCCTAAATCATAATTTATTATGTGA 660

                                                                          680                 700                 720
Eef1a_gDNA_295-8_2.4kb_allele_clone2  TTTGGGAAAGAAATTTGTGTTGATTTTTCCATTAATATTGTAAAAACAAAATCGAAAGTT 718
Eef1a_gDNA_295-8_2.4kb_allele_clone1  TTTGGGAAAGAAATTTGTGTTGATTTTTCCATTAATATTGTAAAAACAAAATCGAAAGTT 718
EF1a_gDNA_295-8_2.4kb_allele_clone3   TTTGGGAAGGAAATTTGTGTTGATTTTTCCATTAATATTGTAAAAACAAAATCGAAAGTT 718
EF1a_gDNA_295-8_2kb_allele_clone2     TTTGGGAAAGAAATTTGTGTTGATTTTTCCATTAATATTGTGAAAACAAAATCGAAAGTT 720
EF1a_gDNA_295-8_2kb_allele_clone3     TTTGGGAAAGAAATTTGTGTTGATTTTTCCATTAATATTGTGAAAACAAAATCGAAAGTT 720
Eef1a_gDNA_295-8_2kb_allele_clone5    TTTGGGAAAGAAATTTGTGTTGATTTTTCCATTAATATTGTGAAAACAAAATCGAAAGTT 720

                                                                          740                 760                 780
Eef1a_gDNA_295-8_2.4kb_allele_clone2  AATTTCTTTCAAAAATTACTTTTAGGTCATCATTCTCAATCATCCAGGTGAGATTCATGC 778
Eef1a_gDNA_295-8_2.4kb_allele_clone1  AATTTCTTTCAAAAATTACTTTTAGGTCATCATTCTCAATCATCCAGGTGAGATTCATGC 778
EF1a_gDNA_295-8_2.4kb_allele_clone3   AATTTCTTTCAAAAATTACTTTTAGGTCATCATTCTCAATCATCCAGGTGAGATTCATGC 778
EF1a_gDNA_295-8_2kb_allele_clone2     AATTTTTTTCAAAAATTACTTTTAGGTCATCATTCTCAATCATCCAGGTGAGATTCATGC 780
EF1a_gDNA_295-8_2kb_allele_clone3     AATTTTTTTCAAAAATTACTTTTAGGTCATCATTCTCAATCATCCAGGTGAGATTCATGC 780
Eef1a_gDNA_295-8_2kb_allele_clone5    AATTTTTTTCAAAAATTACTTTTAGGTCATCATTCTCAATCATCCAGGTGAGATTCATGC 780

                                                                          800                 820                 840
Eef1a_gDNA_295-8_2.4kb_allele_clone2  TGGTTACCAACCTGTTTTTGGATTGCCACACTGCTCACGTTGCCTGCAAATTTACAGAATT 838
Eef1a_gDNA_295-8_2.4kb_allele_clone1  TGGTTACCAACCTGTTTTTGGATTGCCACACTGCTCACGTTGCCTGCAAATTTACAGAATT 838
EF1a_gDNA_295-8_2.4kb_allele_clone3   TGGTTACCAACCTGTTTTTGGATTGCCACACTGCTCACGTTGCCTGCAAATTTACAGAATT 838
EF1a_gDNA_295-8_2kb_allele_clone2     TGGTTACCAACCTGTTTTTGGATTGCCACACTGCTCACGTTGCCTGCAAATTTACAGAATT 840
EF1a_gDNA_295-8_2kb_allele_clone3     TGGTTACCAACCTGTTTTTGGATTGCCACACTGCTCACGTTGCCTGCAAATTTACAGAATT 840
Eef1a_gDNA_295-8_2kb_allele_clone5    TGGTTACCAACCTGTTTTTGGATTGCCACACTGCTCACGTTGCCTGCAAATTTACAGAATT 840
```

Supporting Figure S2

```
                                      860                 880                 900
Eef1a_gDNA_295-8_2.4kb_allele_clone2  AAAGCAGAAATGTGATAGACGAAGTGGTAAAGTTTTAGAAGAAAATCCCAAAATGGTTAA 898
Eef1a_gDNA_295-8_2.4kb_allele_clone1  AAAGCAGAAATGTGATAGACGAAGTGGTAAAGTTTTAGAAGAAAATCCCAAAATGGTTAA 898
EF1a_gDNA_295-8_2.4kb_allele_clone3   AAAGCAGAAATGTGATAGACGAAGTGGTAAAGTTTTAGAAGAAAATCCCAAAATGGTTAA 898
EF1a_gDNA_295-8_2kb_allele_clone2     AAAGCAGAAATGTGATAGACGAAGTGGTAAAGTTTTAGAAGAAAATCCCAAAATGGTTAA 900
EF1a_gDNA_295-8_2kb_allele_clone3     AAAGCAGAAATGTGATAGACGAAGTGGTAAAGTTTTAGAAGAAAATCCCAAAATGGTTAA 900
Eef1a_gDNA_295-8_2kb_allele_clone5    AAAGCAGAAATGTGATAGACGAAGTGGTAAAGTTTTAGAAGAAAATCCCAAAATGGTTAA 900

                                      920                 940                 960
Eef1a_gDNA_295-8_2.4kb_allele_clone2  ATCTGGTGATGCTGCTATGGTTACTTTGACTCCTTCTAAGCCTATGTGTGTTGAAGCTTT 958
Eef1a_gDNA_295-8_2.4kb_allele_clone1  ATCTGGTGATGCTGCTATGGTTACTTTGACTCCTTCTAAGCCTATGTGTGTTGAAGCTTT 958
EF1a_gDNA_295-8_2.4kb_allele_clone3   ATCTGGTGATGCTGCTATGGTTACTTTGACTCCTTCTAAGCCTATGTGTGTTGAAGCTTT 958
EF1a_gDNA_295-8_2kb_allele_clone2     ATCTGGTGATGCTGCTATGGTTACTTTGACTCCTTCTAAGCCTATGTGTGTTGAAGCTTT 960
EF1a_gDNA_295-8_2kb_allele_clone3     ATCTGGTGATGCTGCTATGGTTACTTTGACTCCTTCTAAGCCTATGTGTGTTGAAGCTTT 960
Eef1a_gDNA_295-8_2kb_allele_clone5    ATCTGGTGATGCTGCTATGGTTACTTTGACTCCTTCTAAGCCTATGTGTGTTGAAGCTTT 960

                                      980                 1,000               1,020
Eef1a_gDNA_295-8_2.4kb_allele_clone2  CTCTGACTACCAGCCATTGGGTCGTTTTGCTGTACGTGACATGAGACAAACTGTTGCTGT 1018
Eef1a_gDNA_295-8_2.4kb_allele_clone1  CTCTGACTACCAGCCATTGGGTCGTTTTGCTGTACGTGACATGAGACAAACTGTTGCTGT 1018
EF1a_gDNA_295-8_2.4kb_allele_clone3   CTCTGACTACCAGCCATTGGGTCGTTTTGCTGTACGTGACATGAGACAAACTGTTGCTGT 1018
EF1a_gDNA_295-8_2kb_allele_clone2     CTCTGACTACCAGCCATTGGGTCGTTTTGCTGTACGTGACATGAGACAAACTGTTGCTGT 1020
EF1a_gDNA_295-8_2kb_allele_clone3     CTCTGACTACCAGCCATTGGGTCGTTTTGCTGTACGTGACATGAGACAAACTGTTGCTGT 1020
Eef1a_gDNA_295-8_2kb_allele_clone5    CTCTGACTACCAGCCATTGGGTCGTTTTGCTGTACGTGACATGAGACAAACTGTTGCTGT 1020

                                      1,040               1,060               1,080
Eef1a_gDNA_295-8_2.4kb_allele_clone2  TGGAGTTATCAAGAGTGTTGAAAAGGCCGATGCTACTGGTAAAATGACAAAATCAGCTCA 1078
Eef1a_gDNA_295-8_2.4kb_allele_clone1  TGGAGTTATCAAGAGTGTTGAAAAGGCCGATGCTACTGGTAAAATGACAAAATCAGCTCA 1078
EF1a_gDNA_295-8_2.4kb_allele_clone3   TGGAGTTATCAAGAGTGTTGAAAAGGCCGATGCTACTGGTAAAATGACAAAATCAGCTCA 1078
EF1a_gDNA_295-8_2kb_allele_clone2     TGGAGTTATCAAGAGTGTTGAAAAGGCCGATGCTACTGGTAAAATGACAAAATCAGCTCA 1080
EF1a_gDNA_295-8_2kb_allele_clone3     TGGAGTTATCAAGAGTGTTGAAAAGGCCGATGCTACTGGTAAAATGACAAAATCAGCTCA 1080
Eef1a_gDNA_295-8_2kb_allele_clone5    TGGAGTTATCAAGAGTGTTGAAAAGGCCGATGCTACTGGTAAAATGACAAAATCAGCTCA 1080

                                      1,100               1,120               1,140
Eef1a_gDNA_295-8_2.4kb_allele_clone2  AAAGGCTACCGGAAAAAAGAAGTGACCTATGAAAATTAAATACTGCATAATGTTATTCCG 1138
Eef1a_gDNA_295-8_2.4kb_allele_clone1  AAAGGCTACCGGAAAAAAGAAGTGACCTATGAAAATTAAATACTGCATAATGTTATTCCG 1138
EF1a_gDNA_295-8_2.4kb_allele_clone3   AAAGGCTACCGGAAAAAAGAAGTGACCTATGAAAATTAAATACTGCATAATGTTATTCCG 1138
EF1a_gDNA_295-8_2kb_allele_clone2     AAAGGCTACCGGAAAAAAGAAGTGACCTATGAAAATTAAATACTGCATAATGTTATTCCG 1140
EF1a_gDNA_295-8_2kb_allele_clone3     AAAGGCTACCGGAAAAAAGAAGTGACCTATGAAAATTAAATACTGCATAATGTTATTCCG 1140
Eef1a_gDNA_295-8_2kb_allele_clone5    AAAGGCTACCGGAAAAAAGAAGTGACCTATGAAAATTAAATACTGCATAATGTTATTCCG 1140

                                      1,160               1,180               1,200
Eef1a_gDNA_295-8_2.4kb_allele_clone2  AACTAAAATATTTCATCGAAAACATTTGAGGAGGAAAAACATGTAAATTCTGTCAAATAA 1198
Eef1a_gDNA_295-8_2.4kb_allele_clone1  AACTAAAATATTTCATCGAAAACATTTGAGGAGGAAAAACATGTAAATTCTGTCAAATAA 1198
EF1a_gDNA_295-8_2.4kb_allele_clone3   AACTAAAATATTTCATCGAAAACATTTGAGGAGGAAAAACATGTAAATTCTGTCAAATAA 1198
EF1a_gDNA_295-8_2kb_allele_clone2     AACTAAAATATTTCATCGAAAACATTTGAGGAGGAAAAACATGTAAATTCTGTCAAATGA 1200
EF1a_gDNA_295-8_2kb_allele_clone3     AACTAAAATATTTCATCGAAAACATTTGAGGAGGAAAAACATGTAAATTCTGTCAAATGA 1200
Eef1a_gDNA_295-8_2kb_allele_clone5    AACTAAAATATTTCATCGAAAACATTTGAGGAGGAAAAACATGTAAATTCTGTCAAATGA 1200

                                      1,220               1,240               1,260
Eef1a_gDNA_295-8_2.4kb_allele_clone2  AAAAATTGGAGTTTATTTGTTGTATTTTTTTCACTAAATTAGTGTGGCAGTGAATTCCA 1258
Eef1a_gDNA_295-8_2.4kb_allele_clone1  AAAAATTGGAGTTTATTTGTTGTATTTTTTTCACTAAATTAGTGTGGCAGTGAATTCCA 1258
EF1a_gDNA_295-8_2.4kb_allele_clone3   AAAAATTGGAGTTTATTTGTTGTATTTTTTTCACTAAATTAGTGTGGCAGTGAATTCCA 1258
EF1a_gDNA_295-8_2kb_allele_clone2     AAAAATTGGAGTTAATTTGTTGTATTTTTTTCACTAAATTAGTGTGGCAGTGAATTCCA 1260
EF1a_gDNA_295-8_2kb_allele_clone3     AAAAATTGGAGTTAATTTGTTGTATTTTTTTCACTAAATTAGTGTGGCAGTGAATTCCA 1260
Eef1a_gDNA_295-8_2kb_allele_clone5    AAAAATTGGAGTTAATTTGTTGTATTTTTTTCACTAAATTAGTGTGGCAGTGAATTCCA 1260

                                      1,280               1,300               1,320
Eef1a_gDNA_295-8_2.4kb_allele_clone2  TTTAGCTGATTAAAATTCATTTTGCGGCTAAATATTGTCCCGATTTGCGGTCACCAATTA 1318
Eef1a_gDNA_295-8_2.4kb_allele_clone1  TTTAGCTGATTAAAATTCATTTTGCGGCTAAATATTGTCCCGATTTGCGGTCACCAATTA 1318
EF1a_gDNA_295-8_2.4kb_allele_clone3   TTTAGCTGATTAAAATTCATTTTGCGGCTAAATATTGTCCCGATTTGCGGTCACCAATTA 1318
EF1a_gDNA_295-8_2kb_allele_clone2     TTTAGCTGATTAAAATTCATTTTGCGGCTAAATATTGTCCCGATTTGCGGTCACCAATTA 1320
EF1a_gDNA_295-8_2kb_allele_clone3     TTTAGCTGATTAAAATTCATTTTGCGGCTAAATATTGTCCCGATTTGCGGTCACCAATTA 1320
Eef1a_gDNA_295-8_2kb_allele_clone5    TTTAGCTGATTAAAATTCATTTTGCGGCTAAATATTGTCCCGATTTGCGGTCACCAATTA 1320
```

Supporting Figure S2

```
                                                       1,340               1,360               1,380
Eef1a_gDNA_295-8_2.4kb_allele_clone2  ATAGTGCGACCTTACAGAAAAATTTTCTGTCGCGCTAAATTGCAAATGCACCTGGATAAA 1378
Eef1a_gDNA_295-8_2.4kb_allele_clone1  ATAGTGCGACCTTACAGAAAAATTTTCTGTCGCGCTAAATTGCAAATGCACCTGGATAAA 1378
EF1a_gDNA_295-8_2.4kb_allele_clone3   ATAGTGCGACCTTACAGAAAAATTTTCTGTCGCGCTAAATTGCAAATGCACCTGGATAAA 1378
EF1a_gDNA_295-8_2kb_allele_clone2     ATAGTGCGACCTTACAGAAAAA-------------------------------------- 1342
EF1a_gDNA_295-8_2kb_allele_clone3     ATAGTGCGACCTTACAGAAAAA-------------------------------------- 1342
Eef1a_gDNA_295-8_2kb_allele_clone5    ATAGTGCGACCTTACAGAAAAA-------------------------------------- 1342
                                                       1,400               1,420               1,440
Eef1a_gDNA_295-8_2.4kb_allele_clone2  AAAGCGCGAAGAAAATAAAGCGGAATTAATGTTTGCGAGTGTACTTCGTATTCGCCCGAA 1438
Eef1a_gDNA_295-8_2.4kb_allele_clone1  AAAGCGCGAAGAAAATAAAGCGGAATTAATGTTTGCGAGTGTACTTCGTATTCGCCCGAA 1438
EF1a_gDNA_295-8_2.4kb_allele_clone3   AAAGCGCGAAGAAAATAAAGCGGAATTAATGTTTGCGAGTGTACTTCGTATTCGCCCGAA 1438
EF1a_gDNA_295-8_2kb_allele_clone2     --------------ATAAAGCGGAATTAATGTTTGCGAGTGTACTTCGTATTCGCCCGAA 1388
EF1a_gDNA_295-8_2kb_allele_clone3     --------------ATAAAGCGGAATTAATGTTTGCGAGTGTACTTCGTATTCGCCCGAA 1388
Eef1a_gDNA_295-8_2kb_allele_clone5    --------------ATAAAGCGGAATTAATGTTTGCGAGTGTACTTCGTATTCGCCCGAA 1388
                                                       1,460               1,480               1,500
Eef1a_gDNA_295-8_2.4kb_allele_clone2  GGTGTTCTATAAAATTTAACTAAGCCTTTTTTAAACAAAAACAGACGATATCAATCTTCC 1498
Eef1a_gDNA_295-8_2.4kb_allele_clone1  GGTGTTCTATAAAATTTAACTAAGCCTTTTTTAAACAAAAACAGACGATATCAATCTTCT 1498
EF1a_gDNA_295-8_2.4kb_allele_clone3   GGTGTTCTATAAAATTTAACTAAGCCTTTTTTAAACAAAAACAGACGATATCAATCTTCT 1498
EF1a_gDNA_295-8_2kb_allele_clone2     GGTGTTCTATAAAATTTAACTAAGCCTTTTTTAAACAAAAACAGACGATATCAATCTTCT 1448
EF1a_gDNA_295-8_2kb_allele_clone3     GGTGTTCTATAAAATTTAACTAAGCCTTTTTTAAACAAAAACAGACGATATCAATCTTCT 1448
Eef1a_gDNA_295-8_2kb_allele_clone5    GGTGTTCTATAAAATTTAACTAAGCCTTTTTTAAACAAAAACAGACGATATCAATCTTCT 1448
                                                       1,520               1,540               1,560
Eef1a_gDNA_295-8_2.4kb_allele_clone2  TGCATGATACAAAAAAATTTAAGCTTA--GTGTAAATATGGCTTAATG----TTTTTAAG 1552
Eef1a_gDNA_295-8_2.4kb_allele_clone1  TGCATGATACAAAAAAATTTAAGCTTA--GTGTAAATATGGCTTAATG----TTTTTAAG 1552
EF1a_gDNA_295-8_2.4kb_allele_clone3   TGCATGATACAAAAAAATTTAAGCTTA--GTGTAAATATGGCTTAATG----TTTTTAAG 1552
EF1a_gDNA_295-8_2kb_allele_clone2     TGCATGATACAAAAAAATTTGATCTTATAGTGTAAATATGGCTTAAAAATGTTTTTTAAG 1508
EF1a_gDNA_295-8_2kb_allele_clone3     TGCATGATACAAAAAAATTTGATCTTATAGTGTAAATATGGCTTAAAAATGTTTTTTAAG 1508
Eef1a_gDNA_295-8_2kb_allele_clone5    TGCATGATACAAAAAAATTTGATCTTATAGTGTAAATATGGCTTAAAAATGTTTTTTAAG 1508
                                                       1,580               1,600               1,620
Eef1a_gDNA_295-8_2.4kb_allele_clone2  TTCGAGTATTTTCAAGTCATTAAACGGTGTTTTACTATGTGAATATCAACTGTTTGCTCA 1612
Eef1a_gDNA_295-8_2.4kb_allele_clone1  TTCGAGTATTTTCAAGTCATTAAACGGTGTTTTACTATGTGAATATCAACTGTTTGCTCA 1612
EF1a_gDNA_295-8_2.4kb_allele_clone3   TTCGAGTATTTTCAAGTCATTAAACGGTGTTTTACTATGTGAATATCAACTGTTTGCTCA 1612
EF1a_gDNA_295-8_2kb_allele_clone2     TTCGAGTATTTTCAAGTCATTAAACGGTGTTTTACTATGTGAATATCAACTGTTTGCTCA 1568
Eef1a_gDNA_295-8_2kb_allele_clone3    TTCGAGTATTTTCAAGTCATTAAACGGTGTTTTACTATGTGAATATCAACTGTTTGCTCA 1568
Eef1a_gDNA_295-8_2kb_allele_clone5    TTCGAGTATTTTCAAGTCATTAAACGGTGTTTTACTATGTGAATATCAACTGTTTGCTCA 1568
                                                       1,640               1,660               1,680
Eef1a_gDNA_295-8_2.4kb_allele_clone2  CACAATTATCTTCAGTAATAATTTTTACTGCTTAATTATGTTTAAGTTGATGATGATTGC 1672
Eef1a_gDNA_295-8_2.4kb_allele_clone1  CACAATTATCTTCAGTAATAATTTTTACTGCTTAATTATGTTTAAGTTGATGATGATTGC 1672
EF1a_gDNA_295-8_2.4kb_allele_clone3   CACAATTATCTTCAGTAATAATTTTTACTGCTTAATTATGTTTAAGTTGATGATGATTGC 1672
EF1a_gDNA_295-8_2kb_allele_clone2     CACAATTATCTTCAGTAATAATTTTTACTGCTTAATTATGTTCAAGTTGATGATGATTGC 1628
EF1a_gDNA_295-8_2kb_allele_clone3     CACAATTATCTTCAGTAATAATTTTTACTGCTTAATTATGTTCAAGTTGATGATGATTGC 1628
Eef1a_gDNA_295-8_2kb_allele_clone5    CACAATTATCTTCAGTAATAATTTTTACTGCTTAATTATGTTCAAGTTGATGATGATTGC 1628
                                                       1,700               1,720               1,740
Eef1a_gDNA_295-8_2.4kb_allele_clone2  GGTACCGTGTTTGAGTAGCACATCTTGTCTAATAACAGATACCATTTCTAATTGTATGGT 1732
Eef1a_gDNA_295-8_2.4kb_allele_clone1  GGTACCGTGTTTGAGTAGCACATCTTGTCTAATAACAGATACCATTTCTAATTGTATGGT 1732
EF1a_gDNA_295-8_2.4kb_allele_clone3   GGTACCGTGTTTGAGTAGCACATCTTGTCTAATAACAGATACCATTTCTAATTGTATGGT 1732
EF1a_gDNA_295-8_2kb_allele_clone2     GGTACCGTGTTTGAGTAGCACATCTTGTCTAATAACAGATACCATTTCTAATTGTATGGT 1688
EF1a_gDNA_295-8_2kb_allele_clone3     GGTACCGTGTTTGAGTAGCACATCTTGTCTAATAACAGATACCATTTCTAATTGTATGGT 1688
Eef1a_gDNA_295-8_2kb_allele_clone5    GGTACCGTGTTTGAGTAGCACATCTTGTCTAATAACAGATACCATTTCTAATTGTATGGT 1688
                                                       1,760               1,780               1,800
Eef1a_gDNA_295-8_2.4kb_allele_clone2  AGGCATGCTACCGAACATCAAAACGTTGCTCACACGTCGGTTATCCAGTTACAAGGCAAG 1792
Eef1a_gDNA_295-8_2.4kb_allele_clone1  AGGCATGCTACCGAACATCAAAACGTTGCTCACACGTCGGTTATCCAGTTACAAGGCAAG 1792
EF1a_gDNA_295-8_2.4kb_allele_clone3   AGGCATGCTACCGAACATCAAAACGTTGCTCACACGTCGGTTATCCAGTTACAAGGCAAG 1792
EF1a_gDNA_295-8_2kb_allele_clone2     AGGCATGCTACCGAACATCAAAACGTTGCTCATACGTCGGTTATCCAGTTACAAGGCAAG 1748
EF1a_gDNA_295-8_2kb_allele_clone3     AGGCATGCTACCGAACATCAAAACGTTGCTCATACGTCGGTTATCCAGTTACAAGGCAAG 1748
Eef1a_gDNA_295-8_2kb_allele_clone5    AGGCATGCTACCGAACATCAAAACGTTGCTCATACGTCGGTTATCCAGTTACAAGGCAAG 1748
                                                       1,820               1,840               1,860
Eef1a_gDNA_295-8_2.4kb_allele_clone2  ATGTACTAATCGGCCACGGTATTTCGGTATCCAAGAATAAAACGTGTGTGATTGCGTATA 1852
Eef1a_gDNA_295-8_2.4kb_allele_clone1  ATGTACTAATCGGCCACGGTATTTCGGTATCCAAGAATAAAACGTGTGTGATTGCGTATA 1852
EF1a_gDNA_295-8_2.4kb_allele_clone3   ATGTACTAATCGGCCACGGTATTTCGGTATCCAAGAATAAAACGTGTGTGATTGCGTATA 1852
EF1a_gDNA_295-8_2kb_allele_clone2     ATGTACTAATCGGCCACGGTATTTCGGTATCCAAGAATAAAACGTGTGTGATTGCGTATA 1808
EF1a_gDNA_295-8_2kb_allele_clone3     ATGTACTAATCGGCCACGGTATTTCGGTATCCAAGAATAAAACGTGTGTGATTGCGTATA 1808
Eef1a_gDNA_295-8_2kb_allele_clone5    ATGTACTAATCGGCCACGGTATTTCGGTATCCAAGAATAAAACGTGTGTGATTGCGTATA 1808
```

Supporting Figure S2

```
                                          1,880                     1,900                     1,920
Eef1a_gDNA_295-8_2.4kb_allele_clone2  TTAGCGAACATGTGCAACAAGATGTTAAAAACACCTGTTAACTAATAGTCGTTACCATAG 1912
Eef1a_gDNA_295-8_2.4kb_allele_clone1  TTAGCGAACATGTGCAACAAGATGTTAAAAACACCTGTTAACTAATAGTCGTTACCATAG 1912
EF1a_gDNA_295-8_2.4kb_allele_clone3   TTAGCGAACATGTGCAACAAGATGTTAAAAACACCTGTTAACTAATAGTCGTTACCATAG 1912
EF1a_gDNA_295-8_2kb_allele_clone2     TTAGCGAACATGTGCAACAAGATGTTAAAAACACCTGTTAACTAATAGTCGTTACCATAG 1868
EF1a_gDNA_295-8_2kb_allele_clone3     TTAGCGAACATGTGCAACAAGATGTTAAAAACACCTGTTAACTAATAGTCGTTACCATAG 1868
Eef1a_gDNA_295-8_2kb_allele_clone5    TTAGCGAACATGTGCAACAAGATGTTAAAAACACCTGTTAACTAATAGTCGTTACCATAG 1868
                                          1,940                     1,960                     1,980
Eef1a_gDNA_295-8_2.4kb_allele_clone2  TGACTAATATTAATGCAAATGTAAAAGAATTCCGTAAAAAACTTTCAAAGTTCATTTTTA 1972
Eef1a_gDNA_295-8_2.4kb_allele_clone1  TGACTAATATTAATGCAAATGTAAAAGAATTCCGTAAAAAACTTTCAAAGTTCATTTTTA 1972
EF1a_gDNA_295-8_2.4kb_allele_clone3   TGACTAATATTAATGCAAATGTAAAAGAATTCCGTAAAAAACTTTCAAAGTTCATTTTTA 1972
EF1a_gDNA_295-8_2kb_allele_clone2     TGACTAATATTAATGCAAATGTAAAAGAATTCCGTAAAAAACTTTCAAAGTTCATTTTTA 1928
EF1a_gDNA_295-8_2kb_allele_clone3     TGACTAATATTAATGCAAATGTAAAAGAATTCCGTAAAAAACTTTCAAAGTTCATTTTTA 1928
Eef1a_gDNA_295-8_2kb_allele_clone5    TGACTAATATTAATGCAAATGTAAAAGAATTCCGTAAAAAACTTTCAAAGTTCATTTTTA 1928
                                          2,000                     2,020                     2,040
Eef1a_gDNA_295-8_2.4kb_allele_clone2  GGAGAGTTATTTGACAAAACATAATTTTTGCAATTAATTATTCTTACGCTTCATTGAAGA 2032
Eef1a_gDNA_295-8_2.4kb_allele_clone1  GGAGAGTTATTTGACAAAACATAATTTTTGCAATTAATTATTCTTACGCTTCATTGAAGA 2032
EF1a_gDNA_295-8_2.4kb_allele_clone3   GGAGAGTTATTTGACAAAACATAATTTTTGCAATTAATTATTCTTACGCTTCATTGAAGA 2032
EF1a_gDNA_295-8_2kb_allele_clone2     GGAGAGTTATTTGACAAAACATAATTTTTGCAATTAATTATTCTTACGCTTCATTGAAGA 1988
EF1a_gDNA_295-8_2kb_allele_clone3     GGAGAGTTATTTGACAAAACATAATTTTTGCAATTAATTATTCTTACGCTTCATTGAAGA 1988
Eef1a_gDNA_295-8_2kb_allele_clone5    GGAGAGTTATTTGACAAAACATAATTTTTGCAATTAATTATTCTTACGCTTCATTGAAGA 1988
                                          2,060                     2,080                     2,100
Eef1a_gDNA_295-8_2.4kb_allele_clone2  TCATACTTAATAATTTTTTTAATAGTAATTATTAGTTATTCTTGAAAATTGGTGTGGACA 2092
Eef1a_gDNA_295-8_2.4kb_allele_clone1  TCATACTTAATAATTTTTTTAATAGTAATTATTAGTTATTCTTGAAAATTGGTGTGGACA 2092
EF1a_gDNA_295-8_2.4kb_allele_clone3   TCATACTTAATAATTTTTTTAATAGTAATTATTAGTTATTCTTGAAAATTGGTGTGGACA 2092
EF1a_gDNA_295-8_2kb_allele_clone2     TCATACTTTATAATTTTTCTAATAGTAATTATTAGTTATTCTT----------------- 2031
EF1a_gDNA_295-8_2kb_allele_clone3     TCATACTTTATAATTTTTCTAATAGTAATTATTAGTTATTCTT----------------- 2031
Eef1a_gDNA_295-8_2kb_allele_clone5    TCATACTTTATAATTTTTCTAATAGTAATTATTAGTTATTCTT----------------- 2031
                                          2,120                     2,140                     2,160
Eef1a_gDNA_295-8_2.4kb_allele_clone2  CTCAAAACGCGTCGAATGCCCCTCTATTTTTGCTGATACGTCAGCGAGTGTGTCATTCTG 2152
Eef1a_gDNA_295-8_2.4kb_allele_clone1  CTCAAAACGCGTCGAATGCCCCTCTATTTTTGCTGATACGTCAGCGAGTGTGTCATTCTG 2152
EF1a_gDNA_295-8_2.4kb_allele_clone3   CTCAAAACGCGTCGAATGCCCCTCTATTTTTGCTGATACGTCAGCGAGTGTGTCATTCTG 2152
EF1a_gDNA_295-8_2kb_allele_clone2     ------------------------------------------------------------ 2031
EF1a_gDNA_295-8_2kb_allele_clone3     ------------------------------------------------------------ 2031
Eef1a_gDNA_295-8_2kb_allele_clone5    ------------------------------------------------------------ 2031
                                          2,180                     2,200                     2,220
Eef1a_gDNA_295-8_2.4kb_allele_clone2  GCCGCTAGCAGTTGAATTTTTGAACGTTTCACCAGAAAACTAAGTCAGCATTGTTACTTA 2212
Eef1a_gDNA_295-8_2.4kb_allele_clone1  GCCGCTAGCAGTTGAATTTTTGAACGTTTCACCAGAAAACTAAGTCAGCATTGTTACTTA 2212
EF1a_gDNA_295-8_2.4kb_allele_clone3   GCCGCTAGCAGTTGAATTTTTGAACGTTTCACCAGAAAACTAAGTCAGCATTGTTACTTA 2212
EF1a_gDNA_295-8_2kb_allele_clone2     ------------------------------------------------------------ 2031
EF1a_gDNA_295-8_2kb_allele_clone3     ------------------------------------------------------------ 2031
Eef1a_gDNA_295-8_2kb_allele_clone5    ------------------------------------------------------------ 2031
                                          2,240                     2,260                     2,280
Eef1a_gDNA_295-8_2.4kb_allele_clone2  CGTCACTATTGAACCAATAATGACTCATCTCTGCTAACTTAGTACATAATTCATCCAAAA 2272
Eef1a_gDNA_295-8_2.4kb_allele_clone1  CGTCACTATTGAACCAATAATGACTCATCTCTGCTAACTTAGTACATAATTCATCCAAAA 2272
EF1a_gDNA_295-8_2.4kb_allele_clone3   CGTCACTATTGAACCAATAATGACTCATCTCTGCTAACTTAGTACATAATTCATCCAAAA 2272
EF1a_gDNA_295-8_2kb_allele_clone2     ------------------------------------------------------------ 2031
EF1a_gDNA_295-8_2kb_allele_clone3     ------------------------------------------------------------ 2031
Eef1a_gDNA_295-8_2kb_allele_clone5    ------------------------------------------------------------ 2031
                                          2,300                     2,320                     2,340
Eef1a_gDNA_295-8_2.4kb_allele_clone2  TTTCGGCAGCGATGTTGCGTTTATTATTGTGTCTTCGAATGTTAAAACCAAGTTCGTAGA 2332
Eef1a_gDNA_295-8_2.4kb_allele_clone1  TTTCGGCAGCGATGTTGCGTTTATTATTGTGTCTTCGAATGTTAAAACCAAGTTCGTAGA 2332
EF1a_gDNA_295-8_2.4kb_allele_clone3   TTTCGGCAGCGATGTTGCGTTTATTATTGTGTCTCCGAATGTTAAAACCAAGTTCGTAGA 2332
EF1a_gDNA_295-8_2kb_allele_clone2     ------------------------------------------------------------ 2031
EF1a_gDNA_295-8_2kb_allele_clone3     ------------------------------------------------------------ 2031
Eef1a_gDNA_295-8_2kb_allele_clone5    ------------------------------------------------------------ 2031
                                          2,360                     2,380                     2,400
Eef1a_gDNA_295-8_2.4kb_allele_clone2  ATTAAGATCGCAAAATTATCTGCAGGTCGTAGAATTATCGATCGTAGAATTATCTTAAGA 2392
Eef1a_gDNA_295-8_2.4kb_allele_clone1  ATTAAGATCGCAAAATTATCTGCAGATCGTAGAATTATCGATCGTAGAATTATCTTAAGA 2392
EF1a_gDNA_295-8_2.4kb_allele_clone3   ATTAAGATCGCAAAATTATCTGCAGATCGTAGAATTATCGATCGTAGAATTATCTTAAGA 2392
EF1a_gDNA_295-8_2kb_allele_clone2     --------------------------------------------------------AAGA 2035
EF1a_gDNA_295-8_2kb_allele_clone3     --------------------------------------------------------AAGA 2035
Eef1a_gDNA_295-8_2kb_allele_clone5    --------------------------------------------------------AAGA 2035
```

Supporting Figure S2

```
                                             2,420
                                               |
Eef1a_gDNA_295-8_2.4kb_allele_clone2  TCGTAGCGTAAACCAAATGTGAAGT 2417
Eef1a_gDNA_295-8_2.4kb_allele_clone1  TCGTAGCGTAAACCAAATGTGAAGT 2417
EF1a_gDNA_295-8_2.4kb_allele_clone3   TCGTAGCGTAAACCAAATGTGAAGT 2417
EF1a_gDNA_295-8_2kb_allele_clone2     TCGTAGCGTAAACCAAATGTGAAGT 2060
EF1a_gDNA_295-8_2kb_allele_clone3     TCGTAGCGTAAACCAAATGTGAAGT 2060
Eef1a_gDNA_295-8_2kb_allele_clone5    TCGTAGCGTAAACCAAATGTGAAGT 2060
```

S2 Fig. Alignment of cloned *Eef1a* sequences from female colony 295-8. Two PCR amplicons (sized 2.4 kb and 2.0 kb) from the genomic region encoding the 3’ end of *Eef1a* in colony 295-8 were cloned, sequenced (3 clones each), and aligned. This revealed two alleles, with the major differences being a 52 bp and a 313 bp indel. Differences are shown in red boxes. Sequence features are shown on and above the top sequence of the alignment. Green, *Eef1a* mRNA as determined by alignments to 291-10 transcripts. Yellow, *Eef1a* coding sequence.
