# Supplementary material for: CRISPR/Cas9-mediated gene knockin in the hydroid Hydractinia symbiolongicarpus
Source: BMC Genomics. 2018 Sep 3;19:649. doi: 10.1186/s12864-018-5032-z (PMC6122657; doi:10.1186/s12864-018-5032-z)
Supplement: Supplementary file 3 — Alignment of Eef1a alleles from colonies 291–10 and 295–8. Nucleotide alignment of the region encoding exons 3 and 4 of Eef1a from the four alleles in 291–10 and 295–8. Differences are highlighted in red boxes. Sequence features are shown on and above the top sequence of the alignment. Green, mRNA as determined by alignments to 291–10 transcripts. Yellow, coding sequence. Blue, sgRNA_847 binding site. (PDF 4205 kb) [file 12864_2018_5032_MOESM3_ESM.pdf]

```
                                      20                  40                  60
Eef1a_gDNA_291-10_allele_tig00001480  GTAGAAACTGGTGTATTGTCCCCTGGTATGATCGTTACATTTGCCCCTGCCAATGTAACC  60
Eef1a_gDNA_291-10_allele_tig00008702  GTAGAAACTGGTGTATTGTCCCCTGGTATGATCGTTACATTTGCCCCTGCCAATGTAACC  60
Eef1a_gDNA_295-8_2.4kb_allele         GTAGAAACTGGTGTATTGTCCCCTGGTATGATCGTTACATTTGCCCCTGCCAATGTAACC  60
Eef1a_gDNA_295-8_2.0kb_allele         GTAGAAACTGGTGTATTGTCCCCTGGTATGATCGTTACATTTGCCCCTGCCAATGTAACC  60

                                      80                  100                 120
Eef1a_gDNA_291-10_allele_tig00001480  ACTGAGGTCAAATCTGTAGAAATGCACCACGAATCCTTAACTGAAGCACATCCAGGTGAT  120
Eef1a_gDNA_291-10_allele_tig00008702  ACTGAGGTCAAATCTGTAGAAATGCACCACGAATCCTTAACTGAAGCACATCCAGGTGAT  120
Eef1a_gDNA_295-8_2.4kb_allele         ACTGAGGTCAAATCTGTAGAAATGCACCACGAATCCTTAACTGAAGCACATCCAGGTGAT  120
Eef1a_gDNA_295-8_2.0kb_allele         ACTGAGGTCAAATCTGTAGAAATGCACCACGAATCCTTAACTGAAGCACATCCAGGTGAT  120

                                      140                 160                 180
Eef1a_gDNA_291-10_allele_tig00001480  AATGTTGGTTTTAATGTCAAAAATGTGTCTATCAAGGATATCAAACGTGGTATGGTTGCG  180
Eef1a_gDNA_291-10_allele_tig00008702  AATGTTGGTTTTAATGTCAAAAATGTGTCTATCAAGGATATCAAACGTGGTATGGTTGCG  180
Eef1a_gDNA_295-8_2.4kb_allele         AATGTTGGTTTTAATGTCAAAAATGTGTCTATCAAGGATATCAAACGTGGTATGGTTGCG  180
Eef1a_gDNA_295-8_2.0kb_allele         AATGTTGGTTTTAATGTCAAAAATGTGTCTATCAAGGATATCAAACGTGGTATGGTTGCG  180

                                      200                 220                 240
Eef1a_gDNA_291-10_allele_tig00001480  TCTGACAGCAAAAATGATCCAGCCAAAGAAGCCAAAACCTTTTATGCACAGGTATTCTTT  240
Eef1a_gDNA_291-10_allele_tig00008702  TCTGACAGCAAAAATGATCCAGCCAAAGAAGCCAAAACCTTTTATGCACAGGTATTCTTT  240
Eef1a_gDNA_295-8_2.4kb_allele         TCTGACAGCAAAAATGATCCAGCCAAAGAAGCCAAAACCTTTTATGCACAGGTATTCTTT  240
Eef1a_gDNA_295-8_2.0kb_allele         TCTGACAGCAAAAATGATCCAGCCAAAGAAGCCAAAACCTTTTATGCACAGGTATTCTTT  240

                                      260                 280                 300
Eef1a_gDNA_291-10_allele_tig00001480  AATTTTTGTACTTAACTTATCAGTTTGTATAAGCGTGTTGGCCGTGGCTTGCCACTTGCT  300
Eef1a_gDNA_291-10_allele_tig00008702  CATTTTTGTACTTAACTTATCAGTTTGTATAAGCGTGTTGGCCGTGGC------CGTG--  292
Eef1a_gDNA_295-8_2.4kb_allele         AATTTTTGTACTTAACTTATCAGTTTGTATAAGCGTGTTGGCCGTGGCTTGCCACTTGCT  300
Eef1a_gDNA_295-8_2.0kb_allele         AATTTTTGTACTTAACTTATCAGTTTGTATAAGCGTGTTGGCCGTGGCTTGCCACTTGCT  300

                                      320                 340                 360
Eef1a_gDNA_291-10_allele_tig00001480  TAGCATGATAAATCAATCAACTTGACATTCCATCAATTTTGTTCAACAGAAACAGGACCT  360
Eef1a_gDNA_291-10_allele_tig00008702  ------GATAAATCAATCAACTTGACATTCCATCAATTTTGTTCAACAGAAACACGACCT  346
Eef1a_gDNA_295-8_2.4kb_allele         TAGCATGATAAATCAATCAACTTGACATTCCATCAATTTTGTTCAACAGAAACAGGACCT  360
Eef1a_gDNA_295-8_2.0kb_allele         TAGCATGATAAATCAATCAACTTGACATTCCATCAATTTTGTTCAACAGAAACAGGACCT  360

                                      380                 400                 420
Eef1a_gDNA_291-10_allele_tig00001480  TTAAATAGCAAACAAAATATTGAAATAAAACTGTATGATTTTGTCACTATCGTTTATGTG  420
Eef1a_gDNA_291-10_allele_tig00008702  TTAAATAGCAAACAAAATATTGAAATAAAACTGTATGATTTTGTCATTATCGTTTATGTG  406
Eef1a_gDNA_295-8_2.4kb_allele         TTAAATAGCAAACAAAATATTGAAATAAAACTGTATGATTTTGTCACTATCGTTTATGTG  420
Eef1a_gDNA_295-8_2.0kb_allele         TTAAATAGCAAACAAAATATTGAAATAAAACTGTATGATTTTGTCACTATCGTTTATGTG  420

                                      440                 460                 480
Eef1a_gDNA_291-10_allele_tig00001480  CCCACCATGAAAATTTTACAAAGAAGTGGAACTGTTTGAAAGTTCATTAGCAAGAACAAA  480
Eef1a_gDNA_291-10_allele_tig00008702  CCCACCATGAAAATTTTACAAAGAAGTGGAACTGTTTGAAAGTTCATTAGCAAGAACAAA  466
Eef1a_gDNA_295-8_2.4kb_allele         CCCACCATGAAAATTTTACAAAGAAGTGGAACTGTTTGAAAGTTCATTAGCAAGAACAAA  480
Eef1a_gDNA_295-8_2.0kb_allele         CCCACCATGAAAATTTTACAAAGAAGTGGAACTGTTTGAAAGTTCATTAGCAAGAACAAA  480

                                      500                 520                 540
Eef1a_gDNA_291-10_allele_tig00001480  TCCTAGAAATGTTAGATTTGTGTCAAGAATTTC--AAGATTTTTGTTTCTTGGCAAGTTT  538
Eef1a_gDNA_291-10_allele_tig00008702  TCCTAGAAATGTTAGATTTGTGTCAAGAATTTCTTAAGATTTTTGTTTCTTGGCAAGTTT  526
Eef1a_gDNA_295-8_2.4kb_allele         TCCTAGAAATGTTAGATTTGTGTCAAGAATTTC--AAGATTTTTGTTTCTTGGCAAGTTT  538
Eef1a_gDNA_295-8_2.0kb_allele         TCCTAGAAATGTTAGATTTGTGTCAAGAATTTCTTAAGATTTTTGTTTCTTGGCAAGTAT  540
```

Supporting Figure S3

```
                                                    560                 580                 600
Eef1a_gDNA_291-10_allele_tig00001480  TTAGCACTTGCTTAGGGAACTATTTAAAATATTAATTTTTATTGAATAAAGAACCAACCT  598
Eef1a_gDNA_291-10_allele_tig00008702  TTAGCACTTGCTTAGGGAACTATTTAAAATATTAATTTTTATTGAATAAAGAACCAACCT  586
    Eef1a_gDNA_295-8_2.4kb_allele     TTAGCACTTGCTTAGGGAACTATTTAAAATATTAATTTTTATTGAATAAAGAACCAACCT  598
    Eef1a_gDNA_295-8_2.0kb_allele     TTGGCACTTGCTTAGGGAACTATTTAAAATATTAATTTTTATTGAATAAAGAACCAACCT  600

                                                    620                 640                 660
Eef1a_gDNA_291-10_allele_tig00001480  GTTTGACTTTTATTAGTAATAAAGCTAAAATTATGGCCTAAATCATAATTTATTATGTGA  658
Eef1a_gDNA_291-10_allele_tig00008702  GTTTGACTTTTATTAGTAATAAAGCTAAAATTATGGCCTAAATCATAATTTATTATGTGA  646
    Eef1a_gDNA_295-8_2.4kb_allele     GTTTGACTTTTATTAGTAATAAAGCTAAAATTATGGCCTAAATCATAATTTATTATGTGA  658
    Eef1a_gDNA_295-8_2.0kb_allele     GTTTGACTTTTATTAGTAATAAAGCTAAAATTATGGCCTAAATCATAATTTATTATGTGA  660

                                                    680                 700                 720
Eef1a_gDNA_291-10_allele_tig00001480  TTTGGGAAAGAAATTTGTGTTGATTTTTCCATTAATATTGTAAAAACAAAATCGAAAGTT  718
Eef1a_gDNA_291-10_allele_tig00008702  TTTGGGAAAGAAATTTGTGTTGATTTTTCCATTAATATTGTAAAAACAAAATCAAAAGTT  706
    Eef1a_gDNA_295-8_2.4kb_allele     TTTGGGAAAGAAATTTGTGTTGATTTTTCCATTAATATTGTAAAAACAAAATCGAAAGTT  718
    Eef1a_gDNA_295-8_2.0kb_allele     TTTGGGAAAGAAATTTGTGTTGATTTTTCCATTAATATTGTGAAAACAAAATCGAAAGTT  720

                                                    740                 760                 780
Eef1a_gDNA_291-10_allele_tig00001480  AATTTCTTTCAAAAATTACTTTTAGGTCATCATTCTCAATCATCCAGGTGAGATTCATGC  778
Eef1a_gDNA_291-10_allele_tig00008702  AATTTCTTTCAAAAATTACTTTTAGGTCATCATTCTCAATCATCCAGGTGAGATTCATGC  766
    Eef1a_gDNA_295-8_2.4kb_allele     AATTTCTTTCAAAAATTACTTTTAGGTCATCATTCTCAATCATCCAGGTGAGATTCATGC  778
    Eef1a_gDNA_295-8_2.0kb_allele     AATTTTTTTCAAAAATTACTTTTAGGTCATCATTCTCAATCATCCAGGTGAGATTCATGC  780

                                                    800                 820                 840
Eef1a_gDNA_291-10_allele_tig00001480  TGGTTACCAACCTGTTTTGGATTGCCACACTGCTCACGTTGCCTGCAAATTTACAGAATT  838
Eef1a_gDNA_291-10_allele_tig00008702  TGGTTACCAACCTGTTTTGGATTGCCACACTGCTCACGTTGCCTGCAAATTTACAGAATT  826
    Eef1a_gDNA_295-8_2.4kb_allele     TGGTTACCAACCTGTTTTGGATTGCCACACTGCTCACGTTGCCTGCAAATTTACAGAATT  838
    Eef1a_gDNA_295-8_2.0kb_allele     TGGTTACCAACCTGTTTTGGATTGCCACACTGCTCACGTTGCCTGCAAATTTACAGAATT  840

                                                    860                 880                 900
Eef1a_gDNA_291-10_allele_tig00001480  AAAGCAGAAATGTGATAGACGAAGTGGTAAAGTTTTAGAAGAAAATCCCAAAATGGTTAA  898
Eef1a_gDNA_291-10_allele_tig00008702  AAAGCAGAAATGTGATAGACGAAGTGGTAAAGTTTTAGAAGAAAATCCCAAAATGGTTAA  886
    Eef1a_gDNA_295-8_2.4kb_allele     AAAGCAGAAATGTGATAGACGAAGTGGTAAAGTTTTAGAAGAAAATCCCAAAATGGTTAA  898
    Eef1a_gDNA_295-8_2.0kb_allele     AAAGCAGAAATGTGATAGACGAAGTGGTAAAGTTTTAGAAGAAAATCCCAAAATGGTTAA  900

                                                    920                 940                 960
Eef1a_gDNA_291-10_allele_tig00001480  ATCTGGTGATGCTGCTATGGTTACTTTGACTCCTTCTAAGCCTATGTGTGTTGAAGCTTT  958
Eef1a_gDNA_291-10_allele_tig00008702  ATCTGGTGATGCTGCTATGGTTACTTTGACTCCTTCTAAGCCTATGTGTGTTGAAGCTTT  946
    Eef1a_gDNA_295-8_2.4kb_allele     ATCTGGTGATGCTGCTATGGTTACTTTGACTCCTTCTAAGCCTATGTGTGTTGAAGCTTT  958
    Eef1a_gDNA_295-8_2.0kb_allele     ATCTGGTGATGCTGCTATGGTTACTTTGACTCCTTCTAAGCCTATGTGTGTTGAAGCTTT  960

                                                    980                 1,000               1,020
Eef1a_gDNA_291-10_allele_tig00001480  CTCTGACTACCAGCCATTGGGTCGTTTTGCTGTACGTGACATGAGACAAACTGTTGCTGT  1018
Eef1a_gDNA_291-10_allele_tig00008702  CTCTGACTACCAGCCATTGGGTCGTTTTGCTGTACGTGACATGAGACAAACTGTTGCTGT  1006
    Eef1a_gDNA_295-8_2.4kb_allele     CTCTGACTACCAGCCATTGGGTCGTTTTGCTGTACGTGACATGAGACAAACTGTTGCTGT  1018
    Eef1a_gDNA_295-8_2.0kb_allele     CTCTGACTACCAGCCATTGGGTCGTTTTGCTGTACGTGACATGAGACAAACTGTTGCTGT  1020

                                                    1,040               1,060               1,080
Eef1a_gDNA_291-10_allele_tig00001480  TGGAGTTATCAAGAGTGTTGAAAAGGCCGATGCTACTGGTAAAATGACAAAATCAGCTCA  1078
Eef1a_gDNA_291-10_allele_tig00008702  TGGAGTTATCAAGAGTGTTGAAAAGGCCGATGCTACTGGTAAAATGACAAAATCAGCTCA  1066
    Eef1a_gDNA_295-8_2.4kb_allele     TGGAGTTATCAAGAGTGTTGAAAAGGCCGATGCTACTGGTAAAATGACAAAATCAGCTCA  1078
    Eef1a_gDNA_295-8_2.0kb_allele     TGGAGTTATCAAGAGTGTTGAAAAGGCCGATGCTACTGGTAAAATGACAAAATCAGCTCA  1080
```

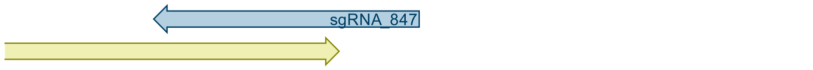


```
                                                   1,100                         1,120                       1,140
Eef1a_gDNA_291-10_allele_tig00001480  AAAGGCTACCGGAAAAAAGAAGTGACCTATGAAAATTAAATACTGCATAATGTTATTCCG 1138
Eef1a_gDNA_291-10_allele_tig00008702  AAAGGCTACCGGAAAAAAGAAGTGACCTATGAAAATTAAATACTGCATAATGTTATTCCG 1126
     Eef1a_gDNA_295-8_2.4kb_allele  AAAGGCTACCGGAAAAAAGAAGTGACCTATGAAAATTAAATACTGCATAATGTTATTCCG 1138
     Eef1a_gDNA_295-8_2.0kb_allele  AAAGGCTACCGGAAAAAAGAAGTGACCTATGAAAATTAAATACTGCATAATGTTATTCCG 1140

                                                   1,160                         1,180                       1,200
Eef1a_gDNA_291-10_allele_tig00001480  AACTAAAATATTTCATCGAAAACATTTGAGGAGGAAAAACATGTAAATTCTGTCAAATAA 1198
Eef1a_gDNA_291-10_allele_tig00008702  AACTAAAATATTTCATCGAAAACATTTGAGGAGGAAAAACATGTAAATTCTGTCAAATGA 1186
     Eef1a_gDNA_295-8_2.4kb_allele  AACTAAAATATTTCATCGAAAACATTTGAGGAGGAAAAACATGTAAATTCTGTCAAATAA 1198
     Eef1a_gDNA_295-8_2.0kb_allele  AACTAAAATATTTCATCGAAAACATTTGAGGAGGAAAAACATGTAAATTCTGTCAAATGA 1200

                                                   1,220                         1,240                       1,260
Eef1a_gDNA_291-10_allele_tig00001480  AAAAATTGGAGTTTATTTGTTGTATTTTTTTCACTAAATTAGTGTGGGCAGTGAATTCCA 1258
Eef1a_gDNA_291-10_allele_tig00008702  AAAAATTGGAGTTAATTTGTTGTATTTTTTTCACTAAATTAGTGTGGGCAGTGAATTCCA 1246
     Eef1a_gDNA_295-8_2.4kb_allele  AAAAATTGGAGTTTATTTGTTGTATTTTTTTCACTAAATTAGTGTGGGCAGTGAATTCCA 1258
     Eef1a_gDNA_295-8_2.0kb_allele  AAAAATTGGAGTTAATTTGTTGTATTTTTTTCACTAAATTAGTGTGGGCAGTGAATTCCA 1260

                                                   1,280                         1,300                       1,320
Eef1a_gDNA_291-10_allele_tig00001480  TTTAGCTGATTAAAATTCATTTTGCGGCTAAATATTGTCCCGATTTGCGGTCACCAATTA 1318
Eef1a_gDNA_291-10_allele_tig00008702  TTTAGCTGATTAAAATTCATTTTGCGGCTAAATATTGTCCCGATTTGCGGTCACCAATTA 1306
     Eef1a_gDNA_295-8_2.4kb_allele  TTTAGCTGATTAAAATTCATTTTGCGGCTAAATATTGTCCCGATTTGCGGTCACCAATTA 1318
     Eef1a_gDNA_295-8_2.0kb_allele  TTTAGCTGATTAAAATTCATTTTGCGGCTAAATATTGTCCCGATTTGCGGTCACCAATTA 1320

                                                   1,340                         1,360                       1,380
Eef1a_gDNA_291-10_allele_tig00001480  ATAGTGCGACCTTACAGAAAAATTTTCTGTCGCGCTAAATTGCAAATGCACCTGGATAAA 1378
Eef1a_gDNA_291-10_allele_tig00008702  ATAGTGCGACCTTACAGAAAAATTTTCTGTCGCACTAAATTGCAAATGCACCTGGATAAA 1366
     Eef1a_gDNA_295-8_2.4kb_allele  ATAGTGCGACCTTACAGAAAAATTTTCTGTCGCGCTAAATTGCAAATGCACCTGGATAAA 1378
     Eef1a_gDNA_295-8_2.0kb_allele  ATAGTGCGACCTTACAGAAAAA-------------------------------------- 1342

                                                   1,400                         1,420                       1,440
Eef1a_gDNA_291-10_allele_tig00001480  AAAGCGCGAAGAAAATAAAGCGGAATTAATGTTTGCGAGTGTACTTCGTATTCGCCCGAA 1438
Eef1a_gDNA_291-10_allele_tig00008702  AAAGCGCGAACAAAATAAAGCGGAATTAATGTTTGCGAGTGTACTTCGTATTCGCCCGAA 1426
     Eef1a_gDNA_295-8_2.4kb_allele  AAAGCGCGAAGAAAATAAAGCGGAATTAATGTTTGCGAGTGTACTTCGTATTCGCCCGAA 1438
     Eef1a_gDNA_295-8_2.0kb_allele  --------------ATAAAGCGGAATTAATGTTTGCGAGTGTACTTCGTATTCGCCCGAA 1388

                                                   1,460                         1,480                       1,500
Eef1a_gDNA_291-10_allele_tig00001480  GGTGTTCTATAAAATTTAACTAAGCCTTTTTTAAACAAAAACAGACGATATCAATCTTCT 1498
Eef1a_gDNA_291-10_allele_tig00008702  GGTGTTCTATAAAATTTAACTAAGCCTTTTTTAAACAAAAACAGACGATATCAATCTTCT 1486
     Eef1a_gDNA_295-8_2.4kb_allele  GGTGTTCTATAAAATTTAACTAAGCCTTTTTTAAACAAAAACAGACGATATCAATCTTCT 1498
     Eef1a_gDNA_295-8_2.0kb_allele  GGTGTTCTATAAAATTTAACTAAGCCTTTTTTAAACAAAAACAGACGATATCAATCTTCT 1448

                                                   1,520                         1,540                       1,560
Eef1a_gDNA_291-10_allele_tig00001480  TGCATGATACAAAAAAAATTTAAGCTTA--GTGTAAATATGGCTTAA---TGTTTTT-AAG 1552
Eef1a_gDNA_291-10_allele_tig00008702  TGCATGATACAAAAAAAATTTGATCTTATAGTGTAAATATGGCTTAAAAATGTTTTT-AAG 1545
     Eef1a_gDNA_295-8_2.4kb_allele  TGCATGATACAAAAAAAATTTAAGCTTA--GTGTAAATATGGCTTAA---TGTTTTT-AAG 1552
     Eef1a_gDNA_295-8_2.0kb_allele  TGCATGATACAAAAAAAATTTGATCTTATAGTGTAAATATGGCTTAAAAATGTTTTTTAAG 1508

                                                   1,580                         1,600                       1,620
Eef1a_gDNA_291-10_allele_tig00001480  TTCGAGTATTTTCAAGTCATTAAACGGTGTTTTACTATGTGAATATCAACTGTTTGCTCA 1612
Eef1a_gDNA_291-10_allele_tig00008702  TTCGAGTATTTTCAAGTCATTAAACGGTGTTTTACTATGTGAATATCAACTGTTTGCTCA 1605
     Eef1a_gDNA_295-8_2.4kb_allele  TTCGAGTATTTTCAAGTCATTAAACGGTGTTTTACTATGTGAATATCAACTGTTTGCTCA 1612
     Eef1a_gDNA_295-8_2.0kb_allele  TTCGAGTATTTTCAAGTCATTAAACGGTGTTTTACTATGTGAATATCAACTGTTTGCTCA 1568

                                                   1,640                         1,660                       1,680
Eef1a_gDNA_291-10_allele_tig00001480  CACAATTATCTTCAGTAATAATTTTTACTGCTTAATTATGTTTAAGTTGATGATGATTGC 1672
Eef1a_gDNA_291-10_allele_tig00008702  CACAATTATCTTCAGTAATAATTTTTACTGCTCAATTATGTTCAAGTTGATGATGATTGC 1665
     Eef1a_gDNA_295-8_2.4kb_allele  CACAATTATCTTCAGTAATAATTTTTACTGCTTAATTATGTTTAAGTTGATGATGATTGC 1672
     Eef1a_gDNA_295-8_2.0kb_allele  CACAATTATCTTCAGTAATAATTTTTACTGCTTAATTATGTTCAAGTTGATGATGATTGC 1628

                                                   1,700                         1,720                       1,740
Eef1a_gDNA_291-10_allele_tig00001480  GGTACCGTGTTTGAGTAGCACATCTTGTCTAATAACAGATACCATTTCTAATTGTATGGT 1732
Eef1a_gDNA_291-10_allele_tig00008702  GGTACCGTGTTTGAGTAGCACATCTTGTCTAATAACAGATACCATTTCTAATTGTATGGT 1725
     Eef1a_gDNA_295-8_2.4kb_allele  GGTACCGTGTTTGAGTAGCACATCTTGTCTAATAACAGATACCATTTCTAATTGTATGGT 1732
     Eef1a_gDNA_295-8_2.0kb_allele  GGTACCGTGTTTGAGTAGCACATCTTGTCTAATAACAGATACCATTTCTAATTGTATGGT 1688

                                                   1,760                         1,780                       1,800
Eef1a_gDNA_291-10_allele_tig00001480  AGGCATGCTACCGAACATCAAAACGTTGCTCACACGTCGGTTATCCAGTTACAAGGCAAG 1792
Eef1a_gDNA_291-10_allele_tig00008702  AGGCATGCTACCGAACATCAAAACGTTGCTCACACTTCGGTTATCCAGTTACAAGGCAAG 1785
     Eef1a_gDNA_295-8_2.4kb_allele  AGGCATGCTACCGAACATCAAAACGTTGCTCACACGTCGGTTATCCAGTTACAAGGCAAG 1792
     Eef1a_gDNA_295-8_2.0kb_allele  AGGCATGCTACCGAACATCAAAACGTTGCTCATACGTCGGTTATCCAGTTACAAGGCAAG 1748
```

```
                                                                       1,820                  1,840                  1,860
Eef1a_gDNA_291-10_allele_tig00001480  ATGTACTAATCGGCCACGGTATTTCGGTATCCAAGAATAAAACGTGTGTGATTGCGTATA 1852
Eef1a_gDNA_291-10_allele_tig00008702  ATGTACTAATCGGCCACGGTATTTCGGTATCCAAGAATAAAACGTGTGTGATTGCGTATA 1845
    Eef1a_gDNA_295-8_2.4kb_allele  ATGTACTAATCGGCCACGGTATTTCGGTATCCAAGAATAAAACGTGTGTGATTGCGTATA 1852
    Eef1a_gDNA_295-8_2.0kb_allele  ATGTACTAATCGGCCACGGTATTTCGGTATCCAAGAATAAAACGTGTGTGATTGCGTATA 1808
                                                                       1,880                  1,900                  1,920
Eef1a_gDNA_291-10_allele_tig00001480  TTAGCGAACATGTGCAACAAGATGTTAAAAACACCTGTTAACTAATAGTCGTTACCATAG 1912
Eef1a_gDNA_291-10_allele_tig00008702  TTAGCGAACATGTGCAACAAAATGTTAAAAACACCTGTTAACTAATAGTCGTTACCATAG 1905
    Eef1a_gDNA_295-8_2.4kb_allele  TTAGCGAACATGTGCAACAAGATGTTAAAAACACCTGTTAACTAATAGTCGTTACCATAG 1912
    Eef1a_gDNA_295-8_2.0kb_allele  TTAGCGAACATGTGCAACAAGATGTTAAAAACACCTGTTAACTAATAGTCGTTACCATAG 1868
                                                                       1,940                  1,960                  1,980
Eef1a_gDNA_291-10_allele_tig00001480  TGACTAATATTAATGCAAATGTAAAAGAATTCCGTAAAAAACTTTCAAAGTTCATTTTTA 1972
Eef1a_gDNA_291-10_allele_tig00008702  TGACTAATATTAATGCAAATGTAAAAGAATTCCGTAAAAAACTTTCAAAGTTCATTTTTA 1965
    Eef1a_gDNA_295-8_2.4kb_allele  TGACTAATATTAATGCAAATGTAAAAGAATTCCGTAAAAAACTTTCAAAGTTCATTTTTA 1972
    Eef1a_gDNA_295-8_2.0kb_allele  TGACTAATATTAATGCAAATGTAAAAGAATTCCGTAAAAAACTTTCAAAGTTCATTTTTA 1928
                                                                       2,000                  2,020                  2,040
Eef1a_gDNA_291-10_allele_tig00001480  GGAGAGTTATTTGACAAAACATAATTTTTGCAATTAATTATTCTTACGCTTCATTGAAGA 2032
Eef1a_gDNA_291-10_allele_tig00008702  GGATAGTTATTTGACAAAACATAATTTTTGCAATTAATTATTCTTACGCTTCATTGAAGA 2025
    Eef1a_gDNA_295-8_2.4kb_allele  GGAGAGTTATTTGACAAAACATAATTTTTGCAATTAATTATTCTTACGCTTCATTGAAGA 2032
    Eef1a_gDNA_295-8_2.0kb_allele  GGAGAGTTATTTGACAAAACATAATTTTTGCAATTAATTATTCTTACGCTTCATTGAAGA 1988
                                                                       2,060                  2,080                  2,100
Eef1a_gDNA_291-10_allele_tig00001480  TCATACTTAATAATTTTTTTAATAGTAATTATTAGTTATTCTTGAAAATTGGTGTGGACA 2092
Eef1a_gDNA_291-10_allele_tig00008702  TCATACTTTATAATTTTTCTAATAGTAATTATTAGTTATTCTTGAAAATTGGTGTGGACA 2085
    Eef1a_gDNA_295-8_2.4kb_allele  TCATACTTAATAATTTTTTTAATAGTAATTATTAGTTATTCTTGAAAATTGGTGTGGACA 2092
    Eef1a_gDNA_295-8_2.0kb_allele  TCATACTTTATAATTTTTCTAATAGTAATTATTAGTTATTCTT----------------- 2031
                                                                       2,120                  2,140                  2,160
Eef1a_gDNA_291-10_allele_tig00001480  CTCAAAACGCGTCGAATGCCCCTCTATTTTTGCTGATACGTCAGCGAGTGTGTCATTCTG 2152
Eef1a_gDNA_291-10_allele_tig00008702  CTCGAAACGCGTCGAATGCCCCTCTATTTTTGCTGATACGTCAGCGAGTGTGTCATTCGG 2145
    Eef1a_gDNA_295-8_2.4kb_allele  CTCAAAACGCGTCGAATGCCCCTCTATTTTTGCTGATACGTCAGCGAGTGTGTCATTCTG 2152
    Eef1a_gDNA_295-8_2.0kb_allele  ------------------------------------------------------------ 2031
                                                                       2,180                  2,200                  2,220
Eef1a_gDNA_291-10_allele_tig00001480  GCCGCTAGCAGTTGAATTTTTGAACGTTTCACCAGAAAACTAAGTCAGCATTGTTACTTA 2212
Eef1a_gDNA_291-10_allele_tig00008702  GCCGCTAGCTGTTGAATTTTTGAACGTTTCACCAAAAAACTAAGTCAGCATTGTTACTTA 2205
    Eef1a_gDNA_295-8_2.4kb_allele  GCCGCTAGCAGTTGAATTTTTGAACGTTTCACCAGAAAACTAAGTCAGCATTGTTACTTA 2212
    Eef1a_gDNA_295-8_2.0kb_allele  ------------------------------------------------------------ 2031
                                                                       2,240                  2,260                  2,280
Eef1a_gDNA_291-10_allele_tig00001480  CGTCACTATTGAACCAATAATGACTCATCTCTGCTAACTTAGTACATAATTCATCCAAAA 2272
Eef1a_gDNA_291-10_allele_tig00008702  CGTCACTATTGAACCAATAATGACTCATCTCTGCTAACTTAGTACATAATTCATCCAAAA 2265
    Eef1a_gDNA_295-8_2.4kb_allele  CGTCACTATTGAACCAATAATGACTCATCTCTGCTAACTTAGTACATAATTCATCCAAAA 2272
    Eef1a_gDNA_295-8_2.0kb_allele  ------------------------------------------------------------ 2031
                                                                       2,300                  2,320                  2,340
Eef1a_gDNA_291-10_allele_tig00001480  TTTCGGCAGCGATGTTGCGTTTATTATTGTGTCTTCGAATGTTAAAACCAAGTTCGTAGA 2332
Eef1a_gDNA_291-10_allele_tig00008702  TTTCGGCAGCGATGTTGCGTTTATTATTGTGTCTTCGAATGTTAAAACCAAGTTCGTAGA 2325
    Eef1a_gDNA_295-8_2.4kb_allele  TTTCGGCAGCGATGTTGCGTTTATTATTGTGTCTTCGAATGTTAAAACCAAGTTCGTAGA 2332
    Eef1a_gDNA_295-8_2.0kb_allele  ------------------------------------------------------------ 2031
                                                                       2,360                  2,380                  2,400
Eef1a_gDNA_291-10_allele_tig00001480  ATTAAGATCGCAAAATTATCTGCAGATCGTAGAATTATCGATCGTAGAATTATCTTAAGA 2392
Eef1a_gDNA_291-10_allele_tig00008702  ATTAAGATCGCAAAATTATCTGCAGATCGTAGAATTATCGATCGTAGAATTATCTTAAGA 2385
    Eef1a_gDNA_295-8_2.4kb_allele  ATTAAGATCGCAAAATTATCTGCAGATCGTAGAATTATCGATCGTAGAATTATCTTAAGA 2392
    Eef1a_gDNA_295-8_2.0kb_allele  --------------------------------------------------------AAGA 2035
                                                                       2,420
Eef1a_gDNA_291-10_allele_tig00001480  TCGTAGCGTAAACCAAATGTGAAGT 2417
Eef1a_gDNA_291-10_allele_tig00008702  TCGTAGCGTAAACCAAATGTGAAGT 2410
    Eef1a_gDNA_295-8_2.4kb_allele  TCGTAGCGTAAACCAAATGTGAAGT 2417
    Eef1a_gDNA_295-8_2.0kb_allele  TCGTAGCGTAAACCAAATGTGAAGT 2060
```

S3 Fig. Alignment of *Eef1a* alleles from colonies 291-10 and 295-8. Nucleotide alignment of the region encoding exons 3 and 4 of *Eef1a* from the four alleles in 291-10 and 295-8. Differences are highlighted in red boxes. Sequence features are shown on and above the top sequence of the alignment. Green, mRNA as determined by alignments to 291-10 transcripts. Yellow, coding sequence. Blue, gRNA_847 binding site.
