# Supplementary material for: CRISPR/Cas9-mediated gene knockin in the hydroid Hydractinia symbiolongicarpus
Source: BMC Genomics. 2018 Sep 3;19:649. doi: 10.1186/s12864-018-5032-z (PMC6122657; doi:10.1186/s12864-018-5032-z)
Supplement: Supplementary file 5 — Alignment of upstream and downstream integration sites from Eef1aFLAG-P2A-eGFP colonies. Nucleotide alignment of the regions encoding the up- and downstream integration sites from the founder colony, 347–10, and two transgenic offspring, 354–3 and 354–5. Three cloned amplicons were sequenced for each animal at each site. Primer binding sites are indicated in green on the expected sequence. Differences are highlighted in red boxes. Note that the regions that do not overlap between the upstream and downstream sequences are shown as gaps at the beginning and end of the alignment. (PDF 4434 kb) [file 12864_2018_5032_MOESM5_ESM.pdf]

```
                                              Pr845
                                              20                  40                  60
expected sequence of Eef1a-FLAG-2A-eGFP allele GTAGAAACTGGTGTATTGTCCCCTGGTATGATCGTTACATTTGCCCCTGCCAATGTAACC 60
347-10 LHA clone 1                             GTAGAAACTGGTGTATTGTCCCCTGGTATGATCGTTACATTTGCCCCTGCCAATGTAACC 60
347-10 LHA clone 2                             GTAGAAACTGGTGTATTGTCCCCTGGTATGATCGTTACATTTGCCCCTGCCAATGTAACC 60
347-10 LHA clone3                              GTAGAAACTGGTGTATTGTCCCCTGGTATGATCGTTACATTTGCCCCTGCCAATGTAACC 60
354-3 LHA clone 1                              GTAGAAACTGGTGTATTGTCCCCTGGTATGATCGTTACATTTGCCCCTGCCAATGTAACC 60
354-3 LHA clone 2                              GTAGAAACTGGTGTATTGTCCCCTGGTATGATCGTTACATTTGCCCCTGCCAATGTAACC 60
354-3 LHA clone 3                              GTAGAAACTGGTGTATTGTCCCCTGGTATGATCGTTACATTTGCCCCTGCCAATGTAACC 60
354-5_L5                                       GTAGAAACTGGTGTATTGTCCCCTGGTATGATCGTTACATTTGCCCCTGCCAATGTAACC 60
354-5_L6                                       GTAGAAACTGGTGTATTGTCCCCTGGTATGATCGTTACATTTGCCCCTGCCAATGTAACC 60
354-5_L8                                       GTAGAAACTGGTGTATTGTCCCCTGGTATGATCGTTACATTTGCCCCTGCCAATGTAACC 60
347-10_R5                                      ------------------------------------------------------------ -
347-10_R6                                      ------------------------------------------------------------ -
347-10_R7                                      ------------------------------------------------------------ -
354-3_R1                                       ------------------------------------------------------------ -
354-3_R2                                       ------------------------------------------------------------ -
354-3_R10                                      ------------------------------------------------------------ -
354-5_R9                                       ------------------------------------------------------------ -
354-5_R11                                      ------------------------------------------------------------ -
354-5_R12                                      ------------------------------------------------------------ -

                                              80                  100                 120
expected sequence of Eef1a-FLAG-2A-eGFP allele ACTGAGGTCAAATCTGTAGAAATGCACCACGAATCCTTAACTGAAGCACATCCAGGTGAT 120
347-10 LHA clone 1                             ACTGAGGTCAAATCTGTAGAAATGCACCACGAATCCTTAACTGAAGCACATCCAGGTGAT 120
347-10 LHA clone 2                             ACTGAGGTCAAATCTGTAGAAATGCACCACGAATCCTTAACTGAAGCACATCCAGGTGAT 120
347-10 LHA clone3                              ACTGAGGTCAAATCTGTAGAAATGCACCACGAATCCTTAACTGAAGCACATCCAGGTGAT 120
354-3 LHA clone 1                              ACTGAGGTCAAATCTGTAGAAATGCACCACGAATCCTTAACTGAAGCACATCCAGGTGAT 120
354-3 LHA clone 2                              ACTGAGGTCAAATCTGTAGAAATGCACCACGAATCCTTAACTGAAGCACATCCAGGTGAT 120
354-3 LHA clone 3                              ACTGAGGTCAAATCTGTAGAAATGCACCACGAATCCTTAACTGAAGCACATCCAGGTGAT 120
354-5_L5                                       ACTGAGGTCAAATCTGTAGAAATGCACCACGAATCCTTAACTGAAGCACATCCAGGTGAT 120
354-5_L6                                       ACTGAGGTCAAATCTGTAGAAATGCACCACGAATCCTTAACTGAAGCACATCCAGGTGAT 120
354-5_L8                                       ACTGAGGTCAAATCTGTAGAAATGCACCACGAATCCTTAACTGAAGCACATCCAGGTGAT 120
347-10_R5                                      ------------------------------------------------------------ -
347-10_R6                                      ------------------------------------------------------------ -
347-10_R7                                      ------------------------------------------------------------ -
354-3_R1                                       ------------------------------------------------------------ -
354-3_R2                                       ------------------------------------------------------------ -
354-3_R10                                      ------------------------------------------------------------ -
354-5_R9                                       ------------------------------------------------------------ -
354-5_R11                                      ------------------------------------------------------------ -
354-5_R12                                      ------------------------------------------------------------ -

                                              140                 160                 180
expected sequence of Eef1a-FLAG-2A-eGFP allele AATGTTGGTTTTAATGTCAAAAATGTGTCTATCAAGGATATCAAACGTGGTATGGTTGCG 180
347-10 LHA clone 1                             AATGTTGGTTTTAATGTCAAAAATGTGTCTATCAAGGATATCAAACGTGGTATGGTTGCG 180
347-10 LHA clone 2                             AATGTTGGTTTTAATGTCAAAAATGTGTCTATCAAGGATATCAAACGTGGTATGGTTGCG 180
347-10 LHA clone3                              AATGTTGGTTTTAATGTCAAAAATGTGTCTATCAAGGATATCAAACGTGGTATGGTTGCG 180
354-3 LHA clone 1                              AATGTTGGTTTTAATGTCAAAAATGTGTCTATCAAGGATATCAAACGTGGTATGGtTGCG 180
354-3 LHA clone 2                              AATGTTGGTTTTAATGTCAAAAATGTGTCTATCAAGGATATCAAACGTGGTATGGTTGCG 180
354-3 LHA clone 3                              AATGTTGGTTTTAATGTCAAAAATGTGTCTATCAAGGATATCAAACGTGGTATGGTtGCG 180
354-5_L5                                       AATGTTGGTTTTAATGTCAAAAATGTGTCTATCAAGGATATCAAACGTGGTATGGTTGCG 180
354-5_L6                                       AATGTTGGTTTTAATGTCAAAAATGTGTCTATCAAGGATATCAAACGTGGTATGGTTGCG 180
354-5_L8                                       AATGTTGGTTTTAATGTCAAAAATGTGTCTATCAAGGATATCAAACGTGGTATGGTTGCG 180
347-10_R5                                      ------------------------------------------------------------ -
347-10_R6                                      ------------------------------------------------------------ -
347-10_R7                                      ------------------------------------------------------------ -
354-3_R1                                       ------------------------------------------------------------ -
354-3_R2                                       ------------------------------------------------------------ -
354-3_R10                                      ------------------------------------------------------------ -
354-5_R9                                       ------------------------------------------------------------ -
354-5_R11                                      ------------------------------------------------------------ -
354-5_R12                                      ------------------------------------------------------------ -
```

```
                                                                          200                 220                 240
                                                                            |                   |                   |
expected sequence of Eef1a-FLAG-2A-eGFP allele  TCTGACAGCAAAAATGATCCAGCCAAAGAAGCCAAAACCTTTTATGCACAGGTATTCTTT 240
                      347-10 LHA clone 1  TCTGACAGCAAAAATGATCCAGCCAAAGAAGCCAAAACCTTTTATGCACAGGTATTCTTT 240
                      347-10 LHA clone 2  TCTGACAGCAAAAATGATCCAGCCAAAGAAGCCAAAACCTTTTATGCACAGGTATTCTTT 240
                       347-10 LHA clone3  TCTGACAGCAAAAATGATCCAGCCAAAGAAGCCAAAACCTTTTATGCACAGGTATTCTTT 240
                       354-3 LHA clone 1  TCTGACAGCAAAAATGATCCAGCCAAAGAAGCCAAAACCTTTTATGCACAGGTATTCTTT 240
                       354-3 LHA clone 2  TCTGACAGCAAAAATGATCCAGCCAAAGAAGCCAAAACCTTTTATGCACAGGTATTCTTT 240
                       354-3 LHA clone 3  TCTGACAGCAAAAATGATCCAGCCAAAGAAGCCAAAACCTTTTATGCACAGGTATTCTTT 240
                                354-5_L5  TCTGACAGCAAAAATGATCCAGCCAAAGAAGCCAAAACCTTTTATGCACAGGTATTCTTT 240
                                354-5_L6  TCTGACAGCAAAAATGATCCAGCCAAAGAAGCCAAAACCTTTTATGCACAGGTATTCTTT 240
                                354-5_L8  TCTGACAGCAAAAATGATCCAGCCAAAGAAGCCAAAACCTTTTATGCACAGGTATTCTTT 240
                               347-10_R5  ------------------------------------------------------------ -
                               347-10_R6  ------------------------------------------------------------ -
                               347-10_R7  ------------------------------------------------------------ -
                                354-3_R1  ------------------------------------------------------------ -
                                354-3_R2  ------------------------------------------------------------ -
                               354-3_R10  ------------------------------------------------------------ -
                                354-5_R9  ------------------------------------------------------------ -
                               354-5_R11  ------------------------------------------------------------ -
                               354-5_R12  ------------------------------------------------------------ -
                                                                          260                 280                 300
                                                                            |                   |                   |
expected sequence of Eef1a-FLAG-2A-eGFP allele  AATTTTTGTACTTAACTTATCAGTTTGTATAAGCGTGTTGGCCGTGGCTTGCCACTTGCT 300
                      347-10 LHA clone 1  AATTTTTGTACTTAACTTATCAGTTTGTATAAGCGTGTTGGCCGTGGCTTGCCACTTGCT 300
                      347-10 LHA clone 2  AATTTTTGTACTTAACTTATCAGTTTGTATAAGCGTGTTGGCCGTGGCTTGCCACTTGCT 300
                       347-10 LHA clone3  AATTTTTGTACTTAACTTATCAGTTTGTATAAGCGTGTTGGCCGTGGCTTGCCACTTGCT 300
                       354-3 LHA clone 1  AATTTTTGTACTTAACTTATCAGTTTGTATAAGCGTGTTGGCCGTGGCTTGCCACTTGCT 300
                       354-3 LHA clone 2  AATTTTTGTACTTAACTTATCAGTTTGTATAAGCGTGTTGGCCGTGGCTTGCCACTTGCT 300
                       354-3 LHA clone 3  AATTTTTGTACTTAACTTATCAGTTTGTATAAGCGTGTTGGCCGTGGCTTGCCACTTGCT 300
                                354-5_L5  AATTTTTGTACTTAACTTATCAGTTTGTATAAGCGTGTTGGCCGTGGCTTGCCACTTGCT 300
                                354-5_L6  AATTTTTGTACTTAACTTATCAGTTTGTATAAGCGTGTTGGCCGTGGCTTGCCACTTGCT 300
                                354-5_L8  AATTTTTGTACTTAACTTATCAGTTTGTATAAGCGTGTTGGCCGTGGCTTGCCACTTGCT 300
                               347-10_R5  ------------------------------------------------------------ -
                               347-10_R6  ------------------------------------------------------------ -
                               347-10_R7  ------------------------------------------------------------ -
                                354-3_R1  ------------------------------------------------------------ -
                                354-3_R2  ------------------------------------------------------------ -
                               354-3_R10  ------------------------------------------------------------ -
                                354-5_R9  ------------------------------------------------------------ -
                               354-5_R11  ------------------------------------------------------------ -
                               354-5_R12  ------------------------------------------------------------ -
                                                                          320                 340                 360
                                                                            |                   |                   |
expected sequence of Eef1a-FLAG-2A-eGFP allele  TAGCATGATAAATCAATCAACTTGACATTCCATCAATTTTGTTCAACAGAAACAGGACCT 360
                      347-10 LHA clone 1  TAGCATGATAAATCAATCAACTTGACATTCCATCAATTTTGTTCAACAGAAACAGGACCT 360
                      347-10 LHA clone 2  TAGCATGATAAATCAATCAACTTGACATTCCATCAATTTTGTTCAACAGAAACAGGACCT 360
                       347-10 LHA clone3  TAGCATGATAAATCAATCAACTTGACATTCCATCAATTTTGTTCAACAGAAACAGGACCT 360
                       354-3 LHA clone 1  TAGCATGATAAATCAATCAACTTGACATTCCATCAATTTTGTTCAACAGAAACAGGACCT 360
                       354-3 LHA clone 2  TAGCATGATAAATCAATCAACTTGACATTCCATCAATTTTGTTCAACAGAAACAGGACCT 360
                       354-3 LHA clone 3  TAGCATGATAAATCAATCAACTTGACATTCCATCAATTTTGTTCAACAGAAACAGGACCT 360
                                354-5_L5  TAGCATGATAAATCAATCAACTTGACATTCCATCAATTTTGTTCAACAGAAACAGGACCT 360
                                354-5_L6  TAGCATGATAAATCAATCAACTTGACATTCCATCAATTTTGTTCAACAGAAACAGGACCT 360
                                354-5_L8  TAGCATGATAAATCAATCAACTTGACATTCCATCAATTTTGTTCAACAGAAACAGGACCT 360
                               347-10_R5  ------------------------------------------------------------ -
                               347-10_R6  ------------------------------------------------------------ -
                               347-10_R7  ------------------------------------------------------------ -
                                354-3_R1  ------------------------------------------------------------ -
                                354-3_R2  ------------------------------------------------------------ -
                               354-3_R10  ------------------------------------------------------------ -
                                354-5_R9  ------------------------------------------------------------ -
                               354-5_R11  ------------------------------------------------------------ -
                               354-5_R12  ------------------------------------------------------------ -
```

	380 400 420	
expected sequence of Eef1a-FLAG-2A-eGFP allele	TTAAATAGCAAACAAAATATTGAAATAAAACTGTATGATTTTGTCACTATCGTTTATGTG	420
347-10 LHA clone 1	TTAAATAGCAAACAAAATATTGAAATAAAACTGTATGATTTTGTCACTATCGTTTATGTG	420
347-10 LHA clone 2	TTAAATAGCAAACAAAATATTGAAATAAAACTGTATGATTTTGTCACTATCGTTTATGTG	420
347-10 LHA clone3	TTAAATAGCAAACAAAATATTGAAATAAAACTGTATGATTTTGTCACTATCGTTTATGTG	420
354-3 LHA clone 1	TTAAATAGCAAACAAAATATTGAAATAAAACTGTATGATTTTGTCACTATCGTTTATGTG	420
354-3 LHA clone 2	TTAAATAGCAAACAAAATATTGAAATAAAACTGTATGATTTTGTCACTATCGTTTATGTG	420
354-3 LHA clone 3	TTAAATAGCAAACAAAATATTGAAATAAAACTGTATGATTTTGTCACTATCGTTTATGTG	420
354-5_L5	TTAAATAGCAAACAAAATATTGAAATAAAACTGTATGATTTTGTCACTATCGTTTATGTG	420
354-5_L6	TTAAATAGCAAACAAAATATTGAAATAAAACTGTATGATTTTGTCACTATCGTTTATGTG	420
354-5_L8	TTAAATAGCAAACAAAATATTGAAATAAAACTGTATGATTTTGTCACTATCGTTTATGTG	420
347-10_R5	--	-
347-10_R6	--	-
347-10_R7	--	-
354-3_R1	--	-
354-3_R2	--	-
354-3_R10	--	-
354-5_R9	--	-
354-5_R11	--	-
354-5_R12	--	-

	440 460 480	
expected sequence of Eef1a-FLAG-2A-eGFP allele	CCCACCATGAAAATTTTACAAAGAAGTGGAACTGTTTGAAAGTTCATTAGCAAGAACAAA	480
347-10 LHA clone 1	CCCACCATGAAAATTTTACAAAGAAGTGGAACTGTTTGAAAGTTCATTAGCAAGAACAAA	480
347-10 LHA clone 2	CCCACCATGAAAATTTTACAAAGAAGTGGAACTGTTTGAAAGTTCATTAGCAAGAACAAA	480
347-10 LHA clone3	CCCACCATGAAAATTTTACAAAGAAGTGGAACTGTTTGAAAGTTCATTAGCAAGAACAAA	480
354-3 LHA clone 1	CCCACCATGAAAATTTTACAAAGAAGTGGAACTGTTTGAAAGTTCATTAGCAAGAACAAA	480
354-3 LHA clone 2	CCCACCATGAAAATTTTACAAAGAAGTGGAACTGTTTGAAAGTTCATTAGCAAGAACAAA	480
354-3 LHA clone 3	CCCACCATGAAAATTTTACAAAGAAGTGGAACTGTTTGAAAGTTCATTAGCAAGAACAAA	480
354-5_L5	CCCACCATGAAAATTTTACAAAGAAGTGGAACTGTTTGAAAGTTCATTAGCAAGAACAAA	480
354-5_L6	CCCACCATGAAAATTTTACAAAGAAGTGGAACTGTTTGAAAGTTCATTAGCAAGAACAAA	480
354-5_L8	CCCACCATGAAAATTTTACAAAGAAGTGGAACTGTTTGAAAGTTCATTAGCAAGAACAAA	480
347-10_R5	--	-
347-10_R6	--	-
347-10_R7	--	-
354-3_R1	--	-
354-3_R2	--	-
354-3_R10	--	-
354-5_R9	--	-
354-5_R11	--	-
354-5_R12	--	-

	500 520 540	
expected sequence of Eef1a-FLAG-2A-eGFP allele	TCCTAGAAATGTTAGATTTGTGTCAAGAATTTCAAGATTTTTGTTTCTTGGCAAGTTTTT	540
347-10 LHA clone 1	TCCTAGAAATGTTAGATTTGTGTCAAGAATTTCAAGATTTTTGTTTCTTGGCAAGTTTTT	540
347-10 LHA clone 2	TCCTAGAAATGTTAGATTTGTGTCAAGAATTTCAAGATTTTTGTTTCTTGGCAAGTTTTT	540
347-10 LHA clone3	TCCTAGAAATGTTAGATTTGTGTCAAGAATTTCAAGATTTTTGTTTCTTGGCAAGTTTTT	540
354-3 LHA clone 1	TCCTAGAAATGTTAGATTTGTGTCAAGAATTTCAAGATTTTTGTTTCTTGGCAAGTTTTT	540
354-3 LHA clone 2	TCCTAGAAATGTTAGATTTGTGTCAAGAATTTCAAGATTTTTGTTTCTTGGCAAGTTTTT	540
354-3 LHA clone 3	TCCTAGAAATGTTAGATTTGTGTCAAGAATTTCAAGATTTTTGTTTCTTGGCAAGTTTTT	540
354-5_L5	TCCTAGAAATGTTAGATTTGTGTCAAGAATTTCAAGATTTTTGTTTCTTGGCAAGTTTTT	540
354-5_L6	TCCTAGAAATGTTAGATTTGTGTCAAGAATTTCAAGATTTTTGTTTCTTGGCAAGTTTTT	540
354-5_L8	TCCTAGAAATGTTAGATTTGTGTCAAGAATTTCAAGATTTTTGTTTCTTGGCAAGTTTTT	540
347-10_R5	--	-
347-10_R6	--	-
347-10_R7	--	-
354-3_R1	--	-
354-3_R2	--	-
354-3_R10	--	-
354-5_R9	--	-
354-5_R11	--	-
354-5_R12	--	-

	560 580 600	
expected sequence of Eef1a-FLAG-2A-eGFP allele	AGCACTTGCTTAGGGAACTATTTAAAATATTAATTTTTATTGAATAAAGAACCAACCTGT	600
347-10 LHA clone 1	AGCACTTGCTTAGGGAACTATTTAAAATATTAATTTTTATTGAATAAAGAACCAACCTGT	600
347-10 LHA clone 2	AGCACTTGCTTAGGGAACTATTTAAAATATTAATTTTTATTGAATAAAGAACCAACCTGT	600
347-10 LHA clone3	AGCACTTGCTTAGGGAACTATTTAAAATATTAATTTTTATTGAATAAAGAACCAACCTGT	600
354-3 LHA clone 1	AGCACTTGCTTAGGGAACTATTTAAAATATTAATTTTTATTGAATAAAGAACCAACCTGT	600
354-3 LHA clone 2	AGCACTTGCTTAGGGAACTATTTAAAATATTAATTTTTATTGAATAAAGAACCAACCTGT	600
354-3 LHA clone 3	AGCACTTGCTTAGGGAACTATTTAAAATATTAATTTTTATTGAATAAAGAACCAACCTGT	600
354-5_L5	AGCACTTGCTTAGGGAACTATTTAAAATATTAATTTTTATTGAATAAAGAACCAACCTGT	600
354-5_L6	AGCACTTGCTTAGGGAACTATTTAAAATATTAATTTTTATTGAATAAAGAACCAACCTGT	600
354-5_L8	AGCACTTGCTTAGGGAACTATTTAAAATATTAATTTTTATTGAATAAAGAACCAACCTGT	600
347-10_R5	--	-
347-10_R6	--	-
347-10_R7	--	-
354-3_R1	--	-
354-3_R2	--	-
354-3_R10	--	-
354-5_R9	--	-
354-5_R11	--	-
354-5_R12	--	-

	620 640 660	
expected sequence of Eef1a-FLAG-2A-eGFP allele	TTGACTTTTATTAGTAATAAAGCTAAAATTATGGCCTAAATCATAATTTATTATGTGATT	660
347-10 LHA clone 1	TTGACTTTTATTAGTAATAAAGCTAAAATTATGGCCTAAATCATAATTTATTATGTGATT	660
347-10 LHA clone 2	TTGACTTTTATTAGTAATAAAGCTAAAATTATGGCCTAAATCATAATTTATTATGTGATT	660
347-10 LHA clone3	TTGACTTTTATTAGTAATAAAGCTAAAATTATGGCCTAAATCATAATTTATTATGTGATT	660
354-3 LHA clone 1	TTGACTTTTATTAGTAATAAAGCTAAAATTATGGCCTAAATCATAATTTATTATGTGATT	660
354-3 LHA clone 2	TTGACTTTTATTAGTAATAAAGCTAAAATTATGGCCTAAATCATAATTTATTATGTGATT	660
354-3 LHA clone 3	TTGACTTTTATTAGTAATAAAGCTAAAATTATGGCCTAAATCATAATTTATTATGTGATT	660
354-5_L5	TTGACTTTTATTAGTAATAAAGCTAAAATTATGGCCTAAATCATAATTTATTATGTGATT	660
354-5_L6	TTGACTTTTATTAGTAATAAAGCTAAAATTATGGCCTAAATCATAATTTATTATGTGATT	660
354-5_L8	TTGACTTTTATTAGTAATAAAGCTAAAATTATGGCCTAAATCATAATTTATTATGTGATT	660
347-10_R5	--	-
347-10_R6	--	-
347-10_R7	--	-
354-3_R1	--	-
354-3_R2	--	-
354-3_R10	--	-
354-5_R9	--	-
354-5_R11	--	-
354-5_R12	--	-

	680 700 720	
expected sequence of Eef1a-FLAG-2A-eGFP allele	TGGGAAAGAAATTTGTGTTGATTTTTCCATTAATATTGTAAAAACAAAATCGAAAGTTAA	720
347-10 LHA clone 1	TGGGAAAGAAATTTGTGTTGATTTTTCCATTAATATTGTAAAAACAAAATCGAAAGTTAA	720
347-10 LHA clone 2	TGGGAAAGAAATTTGTGTTGATTTTTCCATTAATATTGTAAAAACAAAATCGAAAGTTAA	720
347-10 LHA clone3	TGGGAAAGAAATTTGTGTTGATTTTTCCATTAATATTGTAAAAACAAAATCGAAAGTTAA	720
354-3 LHA clone 1	TGGGAAAGAAATTTGTGTTGATTTTTCCATTAATATTGTAAAAACAAAATCGAAAGTTAA	720
354-3 LHA clone 2	TGGGAAAGAAATTTGTGTTGATTTTTCCATTAATATTGTAAAAACAAAATCGAAAGTTAA	720
354-3 LHA clone 3	TGGGAAAGAAATTTGTGTTGATTTTTCCATTAATATTGTAAAAACAAAATCGAAAGTTAA	720
354-5_L5	TGGGAAAGAAATTTGTGTTGATTTTTCCATTAATATTGTAAAAACAAAATCGAAAGTTAA	720
354-5_L6	TGGGAAAGAAATTTGTGTTGATTTTTCCATTAATATTGTAAAAACAAAATCGAAAGTTAA	720
354-5_L8	TGGGAAAGAAATTTGTGTTGATTTTTCCATTAATATTGTAAAAACAAAATCGAAAGTTAA	720
347-10_R5	--	-
347-10_R6	--	-
347-10_R7	--	-
354-3_R1	--	-
354-3_R2	--	-
354-3_R10	--	-
354-5_R9	--	-
354-5_R11	--	-
354-5_R12	--	-

	740 / 760 / 780	
expected sequence of Eef1a-FLAG-2A-eGFP allele	TTTCTTTCAAAAATTACTTTTAGGTCATCATTCTCAATCATCCAGGTGAGATTCATGCTG	780
347-10 LHA clone 1	TTTCTTTCAAAAATTACTTTTAGGTCATCATTCTCAATCATCCAGGTGAGATTCATGCTG	780
347-10 LHA clone 2	TTTCTTTCAAAAATTACTTTTAGGTCATCATTCTCAATCATCCAGGTGAGATTCATGCTG	780
347-10 LHA clone3	TTTCTTTCAAAAATTACTTTTAGGTCATCATTCTCAATCATCCAGGTGAGATTCATGCTG	780
354-3 LHA clone 1	TTTCTTTCAAAAATTACTTTTAGGTCATCATTCTCAATCATCCAGGTGAGATTCATGCTG	780
354-3 LHA clone 2	TTTCTTTCAAAAATTACTTTTAGGTCATCATTCTCAATCATCCAGGTGAGATTCATGCTG	780
354-3 LHA clone 3	TTTCTTTCAAAAATTACTTTTAGGTCATCATTCTCAATCATCCAGGTGAGATTCATGCTG	780
354-5_L5	TTTCTTTCAAAAATTACTTTTAGGTCATCATTCTCAATCATCCAGGTGAGATTCATGCTG	780
354-5_L6	TTTCTTTCAAAAaTTACTTTTAGGTCATCATTCTCAATCATCCAGGTGAGATTCATGCTG	780
354-5_L8	TTTCTTTCAAAAATTACTTTTAGGTCATCATTCTCAATCATCCAGGTGAGATTCATGCTG	780
347-10_R5	--	-
347-10_R6	--	-
347-10_R7	--	-
354-3_R1	--	-
354-3_R2	--	-
354-3_R10	--	-
354-5_R9	--	-
354-5_R11	--	-
354-5_R12	--	-

	800 / 820 / 840	
expected sequence of Eef1a-FLAG-2A-eGFP allele	GTTACCAACCTGTTTTGGATTGCCACACTGCTCACGTTGCCTGCAAATTTACAGAATTAA	840
347-10 LHA clone 1	GTTACCAACCTGTTTTGGATTGCCACACTGCTCACGTTGCCTGCAAATTTACAGAATTAA	840
347-10 LHA clone 2	GTTACCAACCTGTTTTGGATTGCCACACTGCTCACGTTGCCTGCAAATTTACAGAATTAA	840
347-10 LHA clone3	GTTACCAACCTGTTTTGGATTGCCACACTGCTCACGTTGCCTGCAAATTTACAGAATTAA	840
354-3 LHA clone 1	GTTACCAACCTGTTTTGGATTGCCACACTGCTCACGTTGCCTGCAAATTTACAGAATTAA	840
354-3 LHA clone 2	GTTACCAACCTGTTTTGGATTGCCACACTGCTCACGTTGCCTGCAAATTTACAGAATTAA	840
354-3 LHA clone 3	GTTACCAACCTGTTTTGGATTGCCACACTGCTCACGTTGCCTGCAAATTTACAGAATTAA	840
354-5_L5	GTTACCAACCTGTTTTGGATTGCCACACTGCTCACGTTGCCTGCAAATTTACAGAATTAA	840
354-5_L6	GTTACCAACCTGTTTTGGATTGCCACACTGCTCACGTTGCCTGCAAATTTACAGAATTAA	840
354-5_L8	GTTACCAACCTGTTTTGGATTGCCACACTGCTCACGTTGCCTGCAAATTTACAGAATTAA	840
347-10_R5	--	-
347-10_R6	--	-
347-10_R7	--	-
354-3_R1	--	-
354-3_R2	--	-
354-3_R10	--	-
354-5_R9	--	-
354-5_R11	--	-
354-5_R12	--	-

	860 / 880 / 900	
expected sequence of Eef1a-FLAG-2A-eGFP allele	AGCAGAAATGTGATAGACGAAGTGGTAAAGTTTTAGAAGAAAATCCCAAAATGGTTAAAT	900
347-10 LHA clone 1	AGCAGAAATGTGATAGACGAAGTGGTAAAGTTTTAGAAGAAAATCCCAAAATGGTTAAAT	900
347-10 LHA clone 2	AGCAGAAATGTGATAGACGAAGTGGTAAAGTTTTAGAAGAAAATCCCAAAATGGTTAAAT	900
347-10 LHA clone3	AGCAGAAATGTGATAGACGAAGTGGTAAAGTTTTAGAAGAAAATCCCAAAATGGTTAAAT	900
354-3 LHA clone 1	AGCAGAAATGTGATAGACGAAGTGGTAAAGTTTTAGAAGAAAATCCCAAAATGGTTAAAT	900
354-3 LHA clone 2	AGCAGAAATGTGATAGACGAAGTGGTAAAGTTTTAGAAGAAAATCCCAAAATGGTTAAAT	900
354-3 LHA clone 3	AGCAGAAATGTGATAGACGAAGTGGTAAAGTTTTAGAAGAAAATCCCAAAATGGTTAAAT	900
354-5_L5	AGCAGAAATGTGATAGACGAAGTGGTAAAGTTTTAGAAGAAAATCCCAAAATGGTTAAAT	900
354-5_L6	AGCAGAAATGTGATAGACGAAGTGGTAAAGTTTTAGAAGAAAATCCCAAAATGGTTAAAT	900
354-5_L8	AGCAGAAATGTGATAGACGAAGTGGTAAAGTTTTAGAAGAAAATCCCAAAATGGTTAAAT	900
347-10_R5	--	-
347-10_R6	--	-
347-10_R7	--	-
354-3_R1	--	-
354-3_R2	--	-
354-3_R10	--	-
354-5_R9	--	-
354-5_R11	--	-
354-5_R12	--	-

	920 · 940 · 960	
expected sequence of Eef1a-FLAG-2A-eGFP allele	CTGGTGATGCTGCTATGGTTACTTTGACTCCTTCTAAGCCTATGTGTGTTGAAGCTTTCT	960
347-10 LHA clone 1	CTGGTGATGCTGCTATGGTTACTTTGACTCCTTCTAAGCCTATGTGTGTTGAAGCTTTCT	960
347-10 LHA clone 2	CTGGTGATGCTGCTATGGTTACTTTGACTCCTTCTAAGCCTATGTGTGTTGAAGCTTTCT	960
347-10 LHA clone3	CTGGTGATGCTGCTATGGTTACTTTGACTCCTTCTAAGCCTATGTGTGTTGAAGCTTTCT	960
354-3 LHA clone 1	CTGGTGATGCTGCTATGGTTACTTTGACTCCTTCTAAGCCTATGTGTGTTGAAGCTTTCT	960
354-3 LHA clone 2	CTGGTGATGCTGCTATGGTTACTTTGACTCCTTCTAAGCCTATGTGTGTTGAAGCTTTCT	960
354-3 LHA clone 3	CTGGTGATGCTGCTATGGTTACTTTGACTCCTTCTAAGCCTATGTGTGTTGAAGCTTTCT	960
354-5_L5	CTGGTGATGCTGCTATGGTTACTTTGACTCCTTCTAAGCCTATGTGTGTTGAAGCTTTCT	960
354-5_L6	CTGGTGATGCTGCTATGGTTACTTTGACTCCTTCTAAGCCTATGTGTGTTGAAGCTTTCT	960
354-5_L8	CTGGTGATGCTGCTATGGTTACTTTGACTCCTTCTAAGCCTATGTGTGTTGAAGCTTTCT	960
347-10_R5	--	-
347-10_R6	--	-
347-10_R7	--	-
354-3_R1	--	-
354-3_R2	--	-
354-3_R10	--	-
354-5_R9	--	-
354-5_R11	--	-
354-5_R12	--	-

	980 · 1,000 · 1,020	
expected sequence of Eef1a-FLAG-2A-eGFP allele	CTGACTACCAGCCATTGGGTCGTTTTGCTGTACGTGACATGAGACAAACTGTTGCTGTTG	1020
347-10 LHA clone 1	CTGACTACCAGCCATTGGGTCGTTTTGCTGTACGTGACATGAGACAAACTGTTGCTGTTG	1020
347-10 LHA clone 2	CTGACTACCAGCCATTGGGTCGTTTTGCTGTACGTGACATGAGACAAACTGTTGCTGTTG	1020
347-10 LHA clone3	CTGACTACCAGCCATTGGGTCGTTTTGCTGTACGTGACATGAGACAAACTGTTGCTGTTG	1020
354-3 LHA clone 1	CTGACTACCAGCCATTGGGTCGTTTTGCTGTACGTGACATGAGACAAACTGTTGCTGTTG	1020
354-3 LHA clone 2	CTGACTACCAGCCATTGGGTCGTTTTGCTGTACGTGACATGAGACAAACTGTTGCTGTTG	1020
354-3 LHA clone 3	CTGACTACCAGCCATTGGGTCGTTTTGCTGTACGTGACATGAGACAAACTGTTGCTGTTG	1020
354-5_L5	CTGACTACCAGCCATTGGGTCGTTTTGCTGTACGTGACATGAGACAAACTGTTGCTGTTG	1020
354-5_L6	CTGACTACCAGCCATTGGGTCGTTTTGCTGTACGTGACATGAGACAAACTGTTGCTGTTG	1020
354-5_L8	CTGACTACCAGCCATTGGGTCGTTTTGCTGTACGTGACATGAGACAAACTGTTGCTGTTG	1020
347-10_R5	--	-
347-10_R6	--	-
347-10_R7	--	-
354-3_R1	--	-
354-3_R2	--	-
354-3_R10	--	-
354-5_R9	--	-
354-5_R11	--	-
354-5_R12	--	-

	1,040 · 1,060 · 1,080	
expected sequence of Eef1a-FLAG-2A-eGFP allele	GAGTTATCAAGAGTGTTGAAAAGGCCGATGCTACTGGTAAAATGACAAAATCAGCTCAAA	1080
347-10 LHA clone 1	GAGTTATCAAGAGTGTTGAAAAGGCCGATGCTACTGGTAAAATGACAAAATCAGCTCAAA	1080
347-10 LHA clone 2	GAGTTATCAAGAGTGTTGAAAAGGCCGATGCTACTGGTAAAATGACAAAATCAGCTCAAA	1080
347-10 LHA clone3	GAGTTATCAAGAGTGTTGAAAAGGCCGATGCTACTGGTAAAATGACAAAATCAGCTCAAA	1080
354-3 LHA clone 1	GAGTTATCAAGAGTGTTGAAAAGGCCGATGCTACTGGTAAAATGACAAAATCAGCTCAAA	1080
354-3 LHA clone 2	GAGTTATCAAGAGTGTTGAAAAGGCCGATGCTACTGGTAAAATGACAAAATCAGCTCAAA	1080
354-3 LHA clone 3	GAGTTATCAAGAGTGTTGAAAAGGCCGATGCTACTGGTAAAATGACAAAATCAGCTCAAA	1080
354-5_L5	GAGTTATCAAGAGTGTTGAAAAGGCCGATGCTACTGGTAAAATGACAAAATCAGCTCAAA	1080
354-5_L6	GAGTTATCAAGAGTGTTGAAAAGGCCGATGCTACTGGTAAAATGACAAAATCAGCTCAAA	1080
354-5_L8	GAGTTATCAAGAGTGTTGAAAAGGCCGATGCTACTGGTAAAATGACAAAATCAGCTCAAA	1080
347-10_R5	--	-
347-10_R6	--	-
347-10_R7	--	-
354-3_R1	--	-
354-3_R2	--	-
354-3_R10	--	-
354-5_R9	--	-
354-5_R11	--	-
354-5_R12	--	-

```
                                            1xFLAG                     GSG linker     P2A
                                            1,100                1,120                  1,140
expected sequence of Eef1a-FLAG-2A-eGFP allele  AGGCTACCGGAAAGAAAAAAGATTATAAAGATGATGATGATAAAGGTTCAGGTGCTACAA 1140
347-10 LHA clone 1   AGGCTACCGGAAAGAAAAAAGATTATAAAGATGATGATGATAAAGGTTCAGGTGCTACAA 1140
347-10 LHA clone 2   AGGCTACCGGAAAGAAAAAAGATTATAAAGATGATGATGATAAAGGTTCAGGTGCTACAA 1140
347-10 LHA clone3    AGGCTACCGGAAAGAAAAAAGATTATAAAGATGATGATGATAAAGGTTCAGGTGCTACAA 1140
354-3 LHA clone 1    AGGCTACCGGAAAGAAAAAAGATTATAAAGATGATGATGATAAAGGTTCAGGTGCTACAA 1140
354-3 LHA clone 2    AGGCTACCGGAAAGAAAAAAGATTATAAAGATGATGATGATAAAGGTTCAGGTGCTACAA 1140
354-3 LHA clone 3    AGGCTACCGGAAAGAAAAAAGATTATAAAGATGATGATGATAAAGGTTCAGGTGCTACAA 1140
354-5_L5             AGGCTACCGGAAAGAAAAAAGATTATAAAGATGATGATGATAAAGGTTCAGGTGCTACAA 1140
354-5_L6             AGGCTACCGGAAAGAAAAAAGATTATAAAGATGATGATGATAAAGGTTCAGGTGCTACAA 1140
354-5_L8             AGGCTACCGGAAAGAAAAAAGATTATAAAGATGATGATGATAAAGGTTCAGGTGCTACAA 1140
347-10_R5            ------------------------------------------------------------ -
347-10_R6            ------------------------------------------------------------ -
347-10_R7            ------------------------------------------------------------ -
354-3_R1             ------------------------------------------------------------ -
354-3_R2             ------------------------------------------------------------ -
354-3_R10            ------------------------------------------------------------ -
354-5_R9             ------------------------------------------------------------ -
354-5_R11            ------------------------------------------------------------ -
354-5_R12            ------------------------------------------------------------ -

                     P2A                                      Pr876      eGFP
                                          1,160                1,180          1,200
expected sequence of Eef1a-FLAG-2A-eGFP allele  ATTTTTCATTATTAAAACAAGCTGGTGATGTTGAAGAAAATCCAGGTCCAATGAGTAAAG 1200
347-10 LHA clone 1   ATTTTTCATTATTAAAACAAGCTGGTGATGTTGAAGAAAATCCAGGTCCAATGAGTAAAG 1200
347-10 LHA clone 2   ATTTTTCATTATTAAAACAAGCTGGTGATGTTGAAGAAAATCCAGGTCCAATGAGTAAAG 1200
347-10 LHA clone3    ATTTTTCATTATTAAAACAAGCTGGTGATGTTGAAGAAAATCCAGGTCCAATGAGTAAAG 1200
354-3 LHA clone 1    ATTTTTCATTATTAAAACAAGCTGGTGATGTTGAAGAAAATCCAGGTCCAATGAGTAAAG 1200
354-3 LHA clone 2    ATTTTTCATTATTAAAACAAGCTGGTGATGTTGAAGAAAATCCAGGTCCAATGAGTAAAG 1200
354-3 LHA clone 3    ATTTTTCATTATTAAAACAAGCTGGTGATGTTGAAGAAAATCCAGGTCCAATGAGTAAAG 1200
354-5_L5             ATTTTTCATTATTAAAACAAGCTGGTGATGTTGAAGAAAATCCAGGTCCAATGAGTAAAG 1200
354-5_L6             ATTTTTCATTATTAAAACAAGCTGGTGATGTTGAAGAAAATCCAGGTCCAATGAGTAAAG 1200
354-5_L8             ATTTTTCATTATTAAAACAAGCTGGTGATGTTGAAGAAAATCCAGGTCCAATGAGTAAAG 1200
347-10_R5            -----------------------------------------CCAGGTCCAATGAGTAAAG 19
347-10_R6            ------------------------------------------CAGGTCCAATGAGTAAAG 18
347-10_R7            ------------------------------------------CAGGTCCAATGAGTAAAG 18
354-3_R1             -----------------------------------------CCAGGTCCAATGAGTAAAG 19
354-3_R2             -----------------------------------------CCAGGTCCAATGAGTAAAG 19
354-3_R10            -----------------------------------------CCAGGTCCAATGAGTAAAG 19
354-5_R9             -----------------------------------------CCAGGTCCAATGAGTAAAG 19
354-5_R11            ------------------------------------------CAGGTCCAATGAGTAAAG 18
354-5_R12            -----------------------------------------CCAGGTCCAATGAGTAAAG 19
```

Pr876
eGFP

1,220 1,240 1,260

expected sequence of Eef1a-FLAG-2A-eGFP allele	GAGAAGAACTTTTCACTGGAGTTGTCCCAATTCTTGTTGAATTAGATGGTGATGTTAATG	1260
347-10 LHA clone 1	GAGAAGAACTTTTCACTGGAGTTGTCCCAATTCTTGTTGAATTAGATGGTGATGTTAATG	1260
347-10 LHA clone 2	GAGAAGAACTTTTCACTGGAGTTGTCCCAATTCTTGTTGAATTAGATGGTGATGTTAATG	1260
347-10 LHA clone3	GAGAAGAACTTTTCACTGGAGTTGTCCCAATTCTTGTTGAATTAGATGGTGATGTTAATG	1260
354-3 LHA clone 1	GAGAAGAACTTTTCACTGGAGTTGTCCCAATTCTTGTTGAATTAGATGGTGATGTTAATG	1260
354-3 LHA clone 2	GAGAAGAACTTTTCACTGGAGTTGTCCCAATTCTTGTTGAATTAGATGGTGATGTTAATG	1260
354-3 LHA clone 3	GAGAAGAACTTTTCACTGGAGTTGTCCCAATTCTTGTTGAATTAGATGGTGATGTTAATG	1260
354-5_L5	GAGAAGAACTTTTCACTGGAGTTGTCCCAATTCTTGTTGAATTAGATGGTGATGTTAATG	1260
354-5_L6	GAGAAGAACTTTTCACTGGAGTTGTCCCAATTCTTGTTGaATTAGATGGTGATGTTAATG	1260
354-5_L8	GAGAAGAACTTTTCACTGGAGTTGTCCCAATTCTTGTTGAATTAGATGGTGATGTTAATG	1260
347-10_R5	GAGAAGAACTTTTCACTGGAGTTGTCCCAATTCTTGTTGAATTAGATGGTGATGTTAATG	79
347-10_R6	GAGAAGAACTTTTCACTGGAGTTGTCCCAATTCTTGTTGAATTAGATGGTGATGTTAATG	78
347-10_R7	GAGAAGAACTTTTCACTGGAGTTGTCCCAATTCTTGTTGAATTAGATGGTGATGTTAATG	78
354-3_R1	GAGAAGAACTTTTCACTGGAGTTGTCCCAATTCTTGTTGAATTAGATGGTGATGTTAATG	79
354-3_R2	GAGAAGAACTTTTCACTGGAGTTGTCCCAATTCTTGTTGAATTAGATGGTGATGTTAATG	79
354-3_R10	GAGAAGAACTTTTCACTGGAGTTGTCCCAATTCTTGTTGAATTAGATGGTGATGTTAATG	79
354-5_R9	GAGAAGAACTTTTCACTGGAGTTGTCCCAATTCTTGTTGAATTAGATGGTGATGTTAATG	79
354-5_R11	GAGAAGAACTTTTCACTGGAGTTGTCCCAATTCTTGTTGAATTAGATGGTGATGTTAATG	78
354-5_R12	GAGAAGAACTTTTCACTGGAGTTGTCCCAATTCTTGTTGAATTAGATGGTGATGTTAATG	79

eGFP

1,280 1,300 1,320

expected sequence of Eef1a-FLAG-2A-eGFP allele	GGCACAAATTTTCTGTCAGTGGAGAGGGTGAAGGTGATGCAACATACGGAAAACTTACCC	1320
347-10 LHA clone 1	GGCACAAATTTTCTGTCAGTGGAGAGGGTGAAGGTGATGCAACATACGGAAAACTTACCC	1320
347-10 LHA clone 2	GGCACAAATTTTCTGTCAGTGGAGAGGGTGAAGGTGATGCAACATACGGAAAACTTACCC	1320
347-10 LHA clone3	GGCACAAATTTTCTGTCAGTGGAGAGGGTGAAGGTGATGCAACATACGGAAAACTTACCC	1320
354-3 LHA clone 1	GGCACAAATTTTCTGTCAGTGGAGAGGGTGAAGGTGATGCAACATACGGAAAACTTACCC	1320
354-3 LHA clone 2	GGCACAAATTTTCTGTCAGTGGAGAGGGTGAAGGTGATGCAACATACGGAAAACTTACCC	1320
354-3 LHA clone 3	GGCACAAATTTTCTGTCAGTGGAGAGGGTGAAGGTGATGCAACATACGGAAAACTTACCC	1320
354-5_L5	GGCACAAATTTTCTGTCAGTGGAGAGGGTGAAGGTGATGCAACATACGGAAAACTTACCC	1320
354-5_L6	GGCACAAATTTTCTGTCAGTGGAGAGGGTGAAGGTGATGCAACATACGGAAAACTTACCC	1320
354-5_L8	GGCACAAATTTTCTGTCAGTGGAGAGGGTGAAGGTGATGCAACATACGGAAAACTTACCC	1320
347-10_R5	GGCACAAATTTTCTGTCAGTGGAGAGGGTGAAGGTGATGCAACATACGGAAAACTTACCC	139
347-10_R6	GGCACAAATTTTCTGTCAGTGGAGAGGGTGAAGGTGATGCAACATACGGAAAACTTACCC	138
347-10_R7	GGCACAAATTTTCTGTCAGTGGAGAGGGTGAAGGTGATGCAACATACGGAAAACTTACCC	138
354-3_R1	GGCACAAATTTTCTGTCAGTGGAGAGGGTGAAGGTGATGCAACATACGGAAAACTTACCC	139
354-3_R2	GGCACAAATTTTCTGTCAGTGGAGAGGGTGAAGGTGATGCAACATACGGAAAACTTACCC	139
354-3_R10	GGCACAAATTTTCTGTCAGTGGAGAGGGTGAAGGTGATGCAACATACGGAAAACTTACCC	139
354-5_R9	GGCACAAATTTTCTGTCAGTGGAGAGGGTGAAGGTGATGCAACATACGGAAAACTTACCC	139
354-5_R11	GGCACAAATTTTCTGTCAGTGGAGAGGGTGAAGGTGATGCAACATACGGAAAACTTACCC	138
354-5_R12	GGCACAAATTTTCTGTCAGTGGAGAGGGTGAAGGTGATGCAACATACGGAAAACTTACCC	139

eGFP

1,340 1,360 1,380

expected sequence of Eef1a-FLAG-2A-eGFP allele	TTAAATTTATTTGCACTACTGGAAAACTACCTGTTCCATGGCCAACACTTGTCACTACTT	1380
347-10 LHA clone 1	TTAAATTTATTTGCACTACTGGAAAACTACCTGTTCCATGGCCAACACTTGTCACTACTT	1380
347-10 LHA clone 2	TTAAATTTATTTGCACTACTGGAAAACTACCTGTTCCATGGCCAACACTTGTCACTACTT	1380
347-10 LHA clone3	TTAAATTTATTTGCACTACTGGAAAACTACCTGTTCCATGGCCAACACTTGTCACTACTT	1380
354-3 LHA clone 1	TTAAATTTATTTGCACTACTGGAAAACTACCTGTTCCATGGCCAACACTTGTCACTACTT	1380
354-3 LHA clone 2	TTAAATTTATTTGCACTACTGGAAAACTACCTGTTCCATGGCCAACACTTGTCACTACTT	1380
354-3 LHA clone 3	TTAAATTTATTTGCACTACTGGAAAACTACCTGTTCCATGGCCAACACTTGTCACTACTT	1380
354-5_L5	TTAAATTTATTTGCACTACTGGAAAACTACCTGTTCCATGGCCAACACTTGTCACTACTT	1380
354-5_L6	TTAAATTTATTTGCACTACTGGAAAACTACCTGTTCCATGGCCAACACTTGTCACTACTT	1380
354-5_L8	TTAAATTTATTTGCACTACTGGAAAACTACCTGTTCCATGGCCAACACTTGTCACTACTT	1380
347-10_R5	TTAAATTTATTTGCACTACTGGAAAACTACCTGTTCCATGGCCAACACTTGTCACTACTT	199
347-10_R6	TTAAATTTATTTGCACTACTGGAAAACTACCTGTTCCATGGCCAACACTTGTCACTACTT	198
347-10_R7	TTAAATTTATTTGCACTACTGGAAAACTACCTGTTCCATGGCCAACACTTGTCACTACTT	198
354-3_R1	TTAAATTTATTTGCACTACTGGAAAACTACCTGTTCCATGGCCAACACTTGTCACTACTT	199
354-3_R2	TTAAATTTATTTGCACTACTGGAAAACTACCTGTTCCATGGCCAACACTTGTCACTACTT	199
354-3_R10	TTAAATTTATTTGCACTACTGGAAAACTACCTGTTCCATGGCCAACACTTGTCACTACTT	199
354-5_R9	TTAAATTTATTTGCACTACTGGAAAACTACCTGTTCCATGGCCAACACTTGTCACTACTT	199
354-5_R11	TTAAATTTATTTGCACTACTGGAAAACTACCTGTTCCATGGCCAACACTTGTCACTACTT	198
354-5_R12	TTAAATTTATTTGCACTACTGGAAAACTACCTGTTCCATGGCCAACACTTGTCACTACTT	199

eGFP

1,400 1,420 1,440

expected sequence of Eef1a-FLAG-2A-eGFP allele	TCTGTTATGGTGTTCAATGCTTTTCAAGATACCCAGATCATATGAAACGGCATGACTTTT	1440
347-10 LHA clone 1	TCTGTTATGGTGTTCAATGCTTTTCAAGATACCCAGATCATATGAAACGGCATGACTTTT	1440
347-10 LHA clone 2	TCTGTTATGGTGTTCAATGCTTTTCAAGATACCCAGATCATATGAAACGGCATGACTTTT	1440
347-10 LHA clone3	TCTGTTATGGTGTTCAATGCTTTTCAAGATACCCAGATCATATGAAACGGCATGACTTTT	1440
354-3 LHA clone 1	TCTGTTATGGTGTTCAATGCTTTTCAAGATACCCAGATCATATGAAACGGCATGACTTTT	1440
354-3 LHA clone 2	TCTGTTATGGTGTTCAATGCTTTTCAAGATACCCAGATCATATGAAACGGCATGACTTTT	1440
354-3 LHA clone 3	TCTGTTATGGTGTTCAATGCTTTTCAAGATACCCAGATCATATGAAACGGCATGACTTTT	1440
354-5_L5	TCTGTTATGGTGTTCAATGCTTTTCAAGATACCCAGATCATATGAAACGGCATGACTTTT	1440
354-5_L6	TCTGTTATGGTGTTCAATGCTTTTCAAGATACCCAGATCATATGAAACGGCATGACTTTT	1440
354-5_L8	TCTGTTATGGTGTTCAATGCTTTTCAAGATACCCAGATCATATGAAACGGCATGACTTTT	1440
347-10_R5	TCTGTTATGGTGTTCAATGCTTTTCAAGATACCCAGATCATATGAAACGGCATGACTTTT	259
347-10_R6	TCTGTTATGGTGTTCAATGCTTTTCAAGATACCCAGATCATATGAAACGGCATGACTTTT	258
347-10_R7	TCTGTTATGGTGTTCAATGCTTTTCAAGATACCCAGATCATATGAAACGGCATGACTTTT	258
354-3_R1	TCTGTTATGGTGTTCAATGCTTTTCAAGATACCCAGATCATATGAAACGGCATGACTTTT	259
354-3_R2	TCTGTTATGGTGTTCAATGCTTTTCAAGATACCCAGATCATATGAAACGGCATGACTTTT	259
354-3_R10	TCTGTTATGGTGTTCAATGCTTTTCAAGATACCCAGATCATATGAAACGGCATGACTTTT	259
354-5_R9	TCTGTTATGGTGTTCAATGCTTTTCAAGATACCCAGATCATATGAAACGGCATGACTTTT	259
354-5_R11	TCTGTTATGGTGTTCAATGCTTTTCAAGATACCCAGATCATATGAAACGGCATGACTTTT	258
354-5_R12	TCTGTTATGGTGTTCAATGCTTTTCAAGATACCCAGATCATATGAAACGGCATGAcTTTT	259

eGFP

1,460 1,480 1,500

expected sequence of Eef1a-FLAG-2A-eGFP allele	TCAAGAGTGCCATGCCCGAAGGTTATGTACAGGAAAGAACTATATTTTTCAAAGATGACG	1500
347-10 LHA clone 1	TCAAGAGTGCCATGCCCGAAGGTTATGTACAGGAAAGAACTATATTTTTCAAAGATGACG	1500
347-10 LHA clone 2	TCAAGAGTGCCATGCCCGAAGGTTATGTACAGGAAAGAACTATATTTTTCAAAGATGACG	1500
347-10 LHA clone3	TCAAGAGTGCCATGCCCGAAGGTTATGTACAGGAAAGAACTATATTTTTCAAAGATGACG	1500
354-3 LHA clone 1	TCAAGAGTGCCATGCCCGAAGGTTATGTACAGGAAAGAACTATATTTTTCAAAGATGACG	1500
354-3 LHA clone 2	TCAAGAGTGCCATGCCCGAAGGTTATGTACAGGAAAGAACTATATTTTTCAAAGATGACG	1500
354-3 LHA clone 3	TCAAGAGTGCCATGCCCGAAGGTTATGTACAGGAAAGAACTATATTTTTCAAAGATGACG	1500
354-5_L5	TCAAGAGTGCCATGCCCGAAGGTTATGTACAGGAAAGAACTATATTTTTCAAAGATGACG	1500
354-5_L6	TCAAGAGTGCCATGCCCGAAGGTTATGTACAGGAAAGAACTATATTTTTCAAAGATGACG	1500
354-5_L8	TCAAGAGTGCCATGCCCGAAGGTTATGTACAGGAAAGAACTATATTTTTCAAAGATGACG	1500
347-10_R5	TCAAGAGTGCCATGCCCGAAGGTTATGTACAGGAAAGAACTATATTTTTCAAAGATGACG	319
347-10_R6	TCAAGAGTGCCATGCCCGAAGGTTATGTACAGGAAAGAACTATATTTTTCAAAGATGACG	318
347-10_R7	TCAAGAGTGCCATGCCCGAAGGTTATGTACAGGAAAGAACTATATTTTTCAAAGATGACG	318
354-3_R1	TCAAGAGTGCCATGCCCGAAGGTTATGTACAGGAAAGAACTATATTTTTCAAAGATGACG	319
354-3_R2	TCAAGAGTGCCATGCCCGAAGGTTATGTACAGGAAAGAACTATATTTTTCAAAGATGACG	319
354-3_R10	TCAAGAGTGCCATGCCCGAAGGTTATGTACAGGAAAGAACTATATTTTTCAAAGATGACG	319
354-5_R9	TCAAGAGTGCCATGCCCGAAGGTTATGTACAGGAAAGAACTATATTTTTCAAAGATGACG	319
354-5_R11	TCAAGAGTGCCATGCCCGAAGGTTATGTACAGGAAAGAACTATATTTTTCAAAGATGACG	318
354-5_R12	TCAAGAGTGCCATGCCCGAAGGTTATGTACAGGAAAGAACTATATTTTTCAAAGATGACG	319

eGFP

1,520 1,540 1,560

expected sequence of Eef1a-FLAG-2A-eGFP allele	GGAACTACAAGACACGTGCTGAAGTCAAGTTTGAAGGTGATACCCTTGTTAATAGAATCG	1560
347-10 LHA clone 1	GGAACTACAAGACACGTGCTGAAGTCAAGTTTGAAGGTGATACCCTTGTTAATAGAATCG	1560
347-10 LHA clone 2	GGAACTACAAGACACGTGCTGAAGTCAAGTTTGAAGGTGATACCCTTGTTAATAGAATCG	1560
347-10 LHA clone3	GGAACTACAAGACACGTGCTGAAGTCAAGTTTGAAGGTGATACCCTTGTTAATAGAATCG	1560
354-3 LHA clone 1	GGAACTACAAGACACGTGCTGAAGTCAAGTTTGAAGGTGATACCCTTGTTAATAGAATCG	1560
354-3 LHA clone 2	GGAACTACAAGACACGTGCTGAAGTCAAGTTTGAAGGTGATACCCTTGTTAATAGAATCG	1560
354-3 LHA clone 3	GGAACTACAAGACACGTGCTGAAGTCAAGTTTGAAGGTGATACCCTTGTTAATAGAATCG	1560
354-5_L5	GGAACTACAAGACACGTGCTGAAGTCAAGTTTGAAGGTGATACCCTTGTTAATAGAATCG	1560
354-5_L6	GGAACTACAAGACACGTGCTGAAGTCAAGTTTGAAGGTGATACCCTTGTTAATAGAATCG	1560
354-5_L8	GGAACTACAAGACACGTGCTGAAGTCAAGTTTGAAGGTGATACCCTTGTTAATAGAATCG	1560
347-10_R5	GGAACTACAAGACACGTGCTGAAGTCAAGTTTGAAGGTGATACCCTTGTTAATAGAATCG	379
347-10_R6	GGAACTACAAGACACGTGCTGAAGTCAAGTTTGAAGGTGATACCCTTGTTAATAGAATCG	378
347-10_R7	GGAACTACAAGACACGTGCTGAAGTCAAGTTTGAAGGTGATACCCTTGTTAATAGAATCG	378
354-3_R1	GGAACTACAAGACACGTGCTGAAGTCAAGTTTGAAGGTGATACCCTTGTTAATAGAATCG	379
354-3_R2	GGAACTACAAGACACGTGCTGAAGTCAAGTTTGAAGGTGATACCCTTGTTAATAGAATCG	379
354-3_R10	GGAACTACAAGACACGTGCTGAAGTCAAGTTTGAAGGTGATACCCTTGTTAATAGAATCG	379
354-5_R9	GGAACTACAAGACACGTGCTGAAGTCAAGTTTGAAGGTGATACCCTTGTTAATAGAATCG	379
354-5_R11	GGAACTACAAGACACGTGCTGAAGTCAAGTTTGAAGGTGATACCCTTGTTAATAGAATCG	378
354-5_R12	GGAACTACAAGACACGTGCTGAAGTCAAGTTTGAAGGTGATACCCTTGTTAATAGAATCG	379

eGFP

1,580 1,600 1,620

expected sequence of Eef1a-FLAG-2A-eGFP allele	AGTTAAAAGGTATTGATTTTAAAGAAGATGGAAACATTCTTGGACACAAATTGGAATACA	1620
347-10 LHA clone 1	AGTTAAAAGGTATTGATTTTAAAGAAGATGGAAACATTCTTGGACACAAATTGGAATACA	1620
347-10 LHA clone 2	AGTTAAAAGGTATTGATTTTAAAGAAGATGGAAACATTCTTGGACACAAATTGGAATACA	1620
347-10 LHA clone3	AGTTAAAAGGTATTGATTTTAAAGAAGATGGAAACATTCTTGGACACAAATTGGAATACA	1620
354-3 LHA clone 1	AGTTAAAAGGTATTGATTTTAAAGAAGATGGAAACATTCTTGGACACAAATTGGAATACA	1620
354-3 LHA clone 2	AGTTAAAAGGTATTGATTTTAAAGAAGATGGAAACATTCTTGGACACAAATTGGAATACA	1620
354-3 LHA clone 3	AGTTAAAAGGTATTGATTTTAAAGAAGATGGAAACATTCTTGGACACAAATTGGAATACA	1620
354-5_L5	AGTTAAAAGGTATTGATTTTAAAGAAGATGGAAACATTCTTGGACACAAATTGGAATACA	1620
354-5_L6	AGTTAAAAGGTATTGATTTTAAAGAAGATGGAAACATTCTTGGACACAAATTGGAATACA	1620
354-5_L8	AGTTAAAAGGTATTGATTTTAAAGAAGATGGAAACATTCTTGGACACAAATTGGAATACA	1620
347-10_R5	AGTTAAAAGGTATTGATTTTAAAGAAGATGGAAACATTCTTGGACACAAATTGGAATACA	439
347-10_R6	AGTTAAAAGGTATTGATTTTAAAGAAGATGGAAACATTCTTGGACACAAATTGGAATACA	438
347-10_R7	AGTTAAAAGGTATTGATTTTAAAGAAGATGGAAACATTCTTGGACACAAATTGGAATACA	438
354-3_R1	AGTTAAAAGGTATTGATTTTAAAGAAGATGGAAACATTCTTGGACACAAATTGGAATACA	439
354-3_R2	AGTTAAAAGGTATTGATTTTAAAGAAGATGGAAACATTCTTGGACACAAATTGGAATACA	439
354-3_R10	AGTTAAAAGGTATTGATTTTAAAGAAGATGGAAACATTCTTGGACACAAATTGGAATACA	439
354-5_R9	AGTTAAAAGGTATTGATTTTAAAGAAGATGGAAACATTCTTGGACACAAATTGGAATACA	439
354-5_R11	AGTTAAAAGGTATTGATTTTAAAGAAGATGGAAACATTCTTGGACACAAATTGGAATACA	438
354-5_R12	AGTTAAAAGGTATTGATTTTAAAGAAGATGGAAACATTCTTGGACACAAATTGGAATACA	439

eGFP

1,640 1,660 1,680

expected sequence of Eef1a-FLAG-2A-eGFP allele	ACTATAACTCACACAATGTATACATCATGGCAGACAAACAAAAGAATGGAATCAAAGTTA	1680
347-10 LHA clone 1	ACTATAACTCACACAATGTATACATCATGGCAGACAAACAAAAGAATGGAATCAAAGTTA	1680
347-10 LHA clone 2	ACTATAACTCACACAATGTATACATCATGGCAGACAAACAAAAGAATGGAATCAAAGTTA	1680
347-10 LHA clone3	ACTATAACTCACACAATGTATACATCATGGCAGACAAACAAAAGAATGGAATCAAAGTTA	1680
354-3 LHA clone 1	ACTATAACTCACACAATGTATACATCATGGCAGACAAACAAAAGAATGGAATCAAAGTTA	1680
354-3 LHA clone 2	ACTATAACTCACACAATGTATACATCATGGCAGACAAACAAAAGAATGGAATCAAAGTTA	1680
354-3 LHA clone 3	ACTATAACTCACACAATGTATACATCATGGCAGACAAACAAAAGAATGGAATCAAAGTTA	1680
354-5_L5	ACTATAACTCACACAATGTATACATCATGGCAGACAAACAAAAGAATGGAATCAAAGTTA	1680
354-5_L6	ACTATAACTCACACAATGTATACATCATGGCAGACAAACAAAAGAATGGAATCAAAGTTA	1680
354-5_L8	ACTATAACTCACACAATGTATACATCATGGCAGACAAACAAAAGAATGGAATCAAAGTTA	1680
347-10_R5	ACTATAACTCACACAATGTATACATCATGGCAGACAAACAAAAGAATGGAATCAAAGTTA	499
347-10_R6	ACTATAACTCACACAATGTATACATCATGGCAGACAAACAAAAGAATGGAATCAAAGTTA	498
347-10_R7	ACTATAACTCACACAATGTATACATCATGGCAGACAAACAAAAGAATGGAATCAAAGTTA	498
354-3_R1	ACTATAACTCACACAATGTATACATCATGGCAGACAAACAAAAGAATGGAATCAAAGTTA	499
354-3_R2	ACTATAACTCACACAATGTATACATCATGGCAGACAAACAAAAGAATGGAATCAAAGTTA	499
354-3_R10	ACTATAACTCACGCAATGTATACATCATGGCAGACAAACAAAAGAATGGAATCAAAGTTA	499
354-5_R9	ACTATAACTCACACAATGTATACATCATGGCAGACAAACAAAAGAATGGAATCAAAGTTA	499
354-5_R11	ACTATAACTCACACAATGTATACATCATGGCAGACAAACAAAAGAATGGAATCAAAGTTA	498
354-5_R12	ACTATAACTCACACAATGTATACATCATGGCAGACAAACAAAAGAATGGAATCAAAGTTA	499

eGFP

	1,700 · 1,720 · 1,740	
expected sequence of Eef1a-FLAG-2A-eGFP allele	ACTTCAAAATTAGACACAACATTGAAGATGGAAGCGTTCAACTAGCAGACCATTATCAAC	1740
347-10 LHA clone 1	ACTTCAAAATTAGACACAACATTGAAGATGGAAGCGTTCAACTAGCAGACCATTATCAAC	1740
347-10 LHA clone 2	ACTTCAAAATTAGACACAACATTGAAGATGGAAGCGTTCAACTAGCAGACCATTATCAAC	1740
347-10 LHA clone3	ACTTCAAAATTAGACACAACATTGAAGATGGAAGCGTTCAACTAGCAGACCATTATCAAC	1740
354-3 LHA clone 1	ACTTCAAAATTAGACACAACATTGAAGATGGAAGCGTTCAACTAGCAGACCATTATCAAC	1740
354-3 LHA clone 2	ACTTCAAAATTAGACACAACATTGAAGATGGAAGCGTTCAACTAGCAGACCATTATCAAC	1740
354-3 LHA clone 3	ACTTCAAAATTAGACACAACATTGAAGATGGAAGCGTTCAACTAGCAGACCATTATCAAC	1740
354-5_L5	ACTTCAAAATTAGACACAACATTGAAGATGGAAGCGTTCAACTAGCAGACCATTATCAAC	1740
354-5_L6	ACTTCAAAATTAGACACAACATTGAAGATGGAAGCGTTCAACTAGCAGACCATTATCAAC	1740
354-5_L8	ACTTCAAAATTAGACACAACATTGAAGATGGAAGCGTTCAACTAGCAGACCATTATCAAC	1740
347-10_R5	ACTTCAAAATTAGACACAACATTGAAGATGGAAGCGTTCAACTAGCAGACCATTATCAAC	559
347-10_R6	ACTTCAAAATTAGACACAACATTGAAGATGGAAGCGTTCAACTAGCAGACCATTATCAAC	558
347-10_R7	ACTTCAAAATTAGACACAACATTGAAGATGGAAGCGTTCAACTAGCAGACCATTATCAAC	558
354-3_R1	ACTTCAAAATTAGACACAACATTGAAGATGGAAGCGTTCAACTAGCAGACCATTATCAAC	559
354-3_R2	ACTTCAAAATTAGACACAACATTGAAGATGGAAGCGTTCAACTAGCAGACCATTATCAAC	559
354-3_R10	ACTTCAAAATTAGACACAACATTGAAGATGGAAGCGTTCAACTAGCAGACCATTATCAAC	559
354-5_R9	ACTTCAAAATTAGACACAACATTGAAGATGGAAGCGTTCAACTAGCAGACCATTATCAAC	559
354-5_R11	ACTTCAAAATTAGACACAACATTGAAGATGGAAGCGTTCAACTAGCAGACCATTATCAAC	558
354-5_R12	ACTTCAAAATTAGACACAACATTGAAGATGGAAGCGTTCAACTAGCAGACCATTATCAAC	559

eGFP

	1,760 · 1,780 · 1,800	
expected sequence of Eef1a-FLAG-2A-eGFP allele	AAAATACTCCAATTGGCGATGGCCCTGTCCTTTTACCAGACAACCATTACCTGTCCACAC	1800
347-10 LHA clone 1	AAAATACTCCAATTGGCGATGGCCCTGTCCTTTTACCAGACAACCATTACCTGTCCACAC	1800
347-10 LHA clone 2	AAAATACTCCAATTGGCGATGGCCCTGTCCTTTTACCAGACAACCATTACCTGTCCACAC	1800
347-10 LHA clone3	AAAATACTCCAATTGGCGATGGCCCTGTCCTTTTACCAGACAACCATTACCTGTCCACAC	1800
354-3 LHA clone 1	AAAATACTCCAATTGGCGATGGCCCTGTCCTTTTACCAGACAACCATTACCTGTCCACAC	1800
354-3 LHA clone 2	AAAATACTCCAATTGGCGATGGCCCTGTCCTTTTACCAGACAACCATTACCTGTCCACAC	1800
354-3 LHA clone 3	AAAATACTCCAATTGGCGATGGCCCTGTCCTTTTACCAGACAACCATTACCTGTCCACAC	1800
354-5_L5	AAAATACTCCAATTGGCGATGGCCCTGTCCTTTTACCAGACAACCATTACCTGTCCACAC	1800
354-5_L6	AAAATACTCCAATTGGCGATGGCCCTGTCCTTTTACCAGACAACCATTACCTGTCCACAC	1800
354-5_L8	AAAATACTCCAATTGGCGATGGCCCTGTCCTTTTACCAGACAACCATTACCTGTCCACAC	1800
347-10_R5	AAAATACTCCAATTGGCGATGGCCCTGTCCTTTTACCAGACAACCATTACCTGTCCACAC	619
347-10_R6	AAAATACTCCAATTGGCGATGGCCCTGTCCTTTTACCAGACAACCATTACCTGTCCACAC	618
347-10_R7	AAAATACTCCAATTGGCGATGGCCCTGTCCTTTTACCAGACAACCATTACCTGTCCACAC	618
354-3_R1	AAAATACTCCAATTGGCGATGGCCCTGTCCTTTTACCAGACAACCATTACCTGTCCACAC	619
354-3_R2	AAAATACTCCAATTGGCGATGGCCCTGTCCTTTTACCAGACAACCATTACCTGTCCACAC	619
354-3_R10	AAAATACTCCAATTGGCGATGGCCCTGTCCTTTTACCAGACAACCATTACCTGTCCACAC	619
354-5_R9	AAAATACTCCAATTGGCGATGGCCCTGTCCTTTTACCAGACAACCATTACCTGTCCACAC	619
354-5_R11	AAAATACTCCAATTGGCGATGGCCCTGTCCTTTTACCAGACAACCATTACCTGTCCACAC	618
354-5_R12	AAAATACTCCAATTGGCGATGGCCCTGTCCTTTTACCAGACAACCATTACCTGTCCACAC	619

eGFP Pr875

	1,820 · 1,840 · 1,860	
expected sequence of Eef1a-FLAG-2A-eGFP allele	AATCTGCCCTTTCGAAAGATCCCAACGAAAAGAGAGACCACATGGTCCTTCTTGAGTTTG	1860
347-10 LHA clone 1	AATCTGCCCTTTCGAAAGATCCCAACGAAAA-----------------------------	1831
347-10 LHA clone 2	AATCTGCCCTTTCGAAAGATCCCAACGAAAA-----------------------------	1831
347-10 LHA clone3	AATCTGCCCTTTCGAAAGATCCCAACGAAAA-----------------------------	1831
354-3 LHA clone 1	AATCTGCCCTTTCGAAAGATCCCAACGAAAA-----------------------------	1831
354-3 LHA clone 2	AATCTGCCCTTTCGAAAGATCCCAACGAAAA-----------------------------	1831
354-3 LHA clone 3	AATCTGCCCTTTCGAAAGATCCCAACGAAAA-----------------------------	1831
354-5_L5	AATCTGCCCTTTCGAAAGATCCCAACGAAAA-----------------------------	1831
354-5_L6	AATCTGCCCTTTCGAAAGATCCCAACGAAAA-----------------------------	1831
354-5_L8	AATCTGCCCTTTCGAAAGATCCCAACGAAAA-----------------------------	1831
347-10_R5	AATCTGCCCTTTCGAAAGATCCCAACGAAAAGAGAGACCACATGGTCCTTCTTGAGTTTG	679
347-10_R6	AATCTGCCCTTTCGAAAGATCCCAACGAAAAGAGAGACCACATGGTCCTTCTTGAGTTTG	678
347-10_R7	AATCTGCCCTTTCGAAAGATCCCAACGAAAAGAGAGACCACATGGTCCTTCTTGAGTTTG	678
354-3_R1	AATCTGCCCTTTCGAAAGATCCCAACGAAAAGAGAGACCACATGGTCCTTCTTGAGTTTG	679
354-3_R2	AATCTGCCCTTTCGAAAGATCCCAACGAAAAGAGAGACCACATGGTCCTTCTTGAGTTTG	679
354-3_R10	AATCTGCCCTTTCGAAAGATCCCAACGAAAAGAGAGACCACATGGTCCTTCTTGAGTTTG	679
354-5_R9	AATCTGCCCTTTCGAAAGATCCCAACGAAAAGAGAGACCACATGGTCCTTCTTGAGTTTG	679
354-5_R11	AATCTGCCCTTTCGAAAGATCCCAACGAAAAGAGAGACCACATGGTCCTTCTTGAGTTTG	678
354-5_R12	AATCTGCCCTTTCGAAAGATCCCAACGAAAAGAGAGACCACATGGTCCTTCTTGAGTTTG	679

	eGFP; 1,880; 1,900; 1,920	
expected sequence of Eef1a-FLAG-2A-eGFP allele	TAACAGCTGCTGGGATTACACATGGCATGGATGAACTATACAAATAAAAAAGAAGTGACC	1920
347-10 LHA clone 1	--	1831
347-10 LHA clone 2	--	1831
347-10 LHA clone3	--	1831
354-3 LHA clone 1	--	1831
354-3 LHA clone 2	--	1831
354-3 LHA clone 3	--	1831
354-5_L5	--	1831
354-5_L6	--	1831
354-5_L8	--	1831
347-10_R5	TAACAGCTGCTGGGATTACACATGGCATGGATGAACTATACAAATAAAAAAGAAGTGACC	739
347-10_R6	TAACAGCTGCTGGGATTACACATGGCATGGATGAACTATACAAATAAAAAAGAAGTGACC	738
347-10_R7	TAACAGCTGCTGGGATTACACATGGCATGGATGAACTATACAAATAAAAAAGAAGTGACC	738
354-3_R1	TAACAGCTGCTGGGATTACACATGGCATGGATGAACTATACAAATAAAAAAGAAGTGACC	739
354-3_R2	TAACAGCTGCTGGGATTACACATGGCATGGATGAACTATACAAATAAAAAAGAAGTGACC	739
354-3_R10	TAACAGCTGCTGGGATTACACATGGCATGGATGAACTATACAAATAAAAAAGAAGTGACC	739
354-5_R9	TAACAGCTGCTGGGATTACACATGGCATGGATGAACTATACAAATAAAAAAGAAGTGACC	739
354-5_R11	TAACAGCTGCTGGGATTACACATGGCATGGATGAACTATACAAATAAAAAAGAAGTGACC	738
354-5_R12	TAACAGCTGCTGGGATTACACATGGCATGGATGAACTATACAAATAAAAAAGAAGTGACC	739
	1,940; 1,960; 1,980	
expected sequence of Eef1a-FLAG-2A-eGFP allele	TATGAAAATTAAATACTGCATAATGTTATTCCGAACTAAAATATTTCATCGAAAACATTT	1980
347-10 LHA clone 1	--	1831
347-10 LHA clone 2	--	1831
347-10 LHA clone3	--	1831
354-3 LHA clone 1	--	1831
354-3 LHA clone 2	--	1831
354-3 LHA clone 3	--	1831
354-5_L5	--	1831
354-5_L6	--	1831
354-5_L8	--	1831
347-10_R5	TATGAAAATTAAATACTGCATAATGTTATTCCGAACTAAAATATTTCATCGAAAACATTT	799
347-10_R6	TATGAAAATTAAATACTGCATAATGTTATTCCGAACTAAAATATTTCATCGAAAACATTT	798
347-10_R7	TATGAAAATTAAATACTGCATAATGTTATTCCGAACTAAAATATTTCATCGAAAACATTT	798
354-3_R1	TATGAAAATTAAATACTGCATAATGTTATTCCGAACTAAAATATTTCATCGAAAACATTT	799
354-3_R2	TATGAAAATTAAATACTGCATAATGTTATTCCGAACTAAAATATTTCATCGAAAACATTT	799
354-3_R10	TATGAAAATTAAATACTGCATAATGTTATTCCGAACTAAAATATTTCATCGAAAACATTT	799
354-5_R9	TATGAAAATTAAATACTGCATAATGTTATTCCGAACTAAAATATTTCATCGAAAACATTT	799
354-5_R11	TATGAAAATTAAATACTGCATAATGTTATTCCGAACTAAAATATTTCATCGAAAACATTT	798
354-5_R12	TATGAAAATTAAATACTGCATAATGTTATTCCGAACTAAAATATTTCATCGAAAACATTT	799
	2,000; 2,020; 2,040	
expected sequence of Eef1a-FLAG-2A-eGFP allele	GAGGAGGAAAAACATGTAAATTCTGTCAAATAAAAAAATTGGAGTTTATTTGTTGTATTT	2040
347-10 LHA clone 1	--	1831
347-10 LHA clone 2	--	1831
347-10 LHA clone3	--	1831
354-3 LHA clone 1	--	1831
354-3 LHA clone 2	--	1831
354-3 LHA clone 3	--	1831
354-5_L5	--	1831
354-5_L6	--	1831
354-5_L8	--	1831
347-10_R5	GAGGAGGAAAAACATGTAAATTCTGTCAAATAAAAAAATTGGAGTTTATTTGTTGTATTT	859
347-10_R6	GAGGAGGAAAAACATGTAAATTCTGTCAAATAAAAAAATTGGAGTTTATTTGTTGTATTT	858
347-10_R7	GAGGAGGAAAAACATGTAAATTCTGTCAAATAAAAAAATTGGAGTTTATTTGTTGTATTT	858
354-3_R1	GAGGAGGAAAAACATGTAAATTCTGTCAAATAAAAAAATTGGAGTTTATTTGTTGTATTT	859
354-3_R2	GAGGAGGAAAAACATGTAAATTCTGTCAAATAAAAAAATTGGAGTTTATTTGTTGTATTT	859
354-3_R10	GAGGAGGAAAAACATGTAAATTCTGTCAAATAAAAAAATTGGAGTTTATTTGTTGTATTT	859
354-5_R9	GAGGAGGAAAAACATGTAAATTCTGTCAAATAAAAAAATTGGAGTTTATTTGTTGTATTT	859
354-5_R11	GAGGAGGAAAAACATGTAAATTCTGTCAAATAAAAAAATTGGAGTTTATTTGTTGTATTT	858
354-5_R12	GAGGAGGAAAAACATGTAAATTCTGTCAAATAAAAAAATTGGAGTTTATTTGTTGTATTT	859

	2,060 / 2,080 / 2,100	
expected sequence of Eef1a-FLAG-2A-eGFP allele	TTTTCACTAAATTAGTGTGGGCAGTGAATTCCATTTAGCTGATTAAAATTCATTTTGCGG	2100
347-10 LHA clone 1	--	1831
347-10 LHA clone 2	--	1831
347-10 LHA clone3	--	1831
354-3 LHA clone 1	--	1831
354-3 LHA clone 2	--	1831
354-3 LHA clone 3	--	1831
354-5_L5	--	1831
354-5_L6	--	1831
354-5_L8	--	1831
347-10_R5	TTTTCACTAAATTAGTGTGGGCAGTGAATTCCATTTAGCTGATTAAAATTCATTTTGCGG	919
347-10_R6	TTTTCACTAAATTAGTGTGGGCAGTGAATTCCATTTAGCTGATTAAAATTCATTTTGCGG	918
347-10_R7	TTTTCACTAAATTAGTGTGGGCAGTGAATTCCATTTAGCTGATTAAAATTCATTTTGCGG	918
354-3_R1	TTTTCACTAAATTAGTGTGGGCAGTGAATTCCATTTAGCTGATTAAAATTCATTTTGCGG	919
354-3_R2	TTTTCACTAAATTAGTGTGGGCAGTGAATTCCATTTAGCTGATTAAAATTCATTTTGCGG	919
354-3_R10	TTTTCACTAAATTAGTGTGGGCAGTGAATTCCATTTAGCTGATTAAAATTCATTTTGCGG	919
354-5_R9	TTTTCACTAAATTAGTGTGGGCAGTGAATTCCATTTAGCTGATTAAAATTCATTTTGCGG	919
354-5_R11	TTTTCACTAAATTAGTGTGGGCAGTGAATTCCATTTAGCTGATTAAAATTCATTTTGCGG	918
354-5_R12	TTTTCACTAAATTAGTGTGGGCAGTGAATTCCATTTAGCTGATTAAAATTCATTTTGCGG	919

	2,120 / 2,140 / 2,160	
expected sequence of Eef1a-FLAG-2A-eGFP allele	CTAAATATTGTCCCGATTTGCGGTCACCAATTAATAGTGCGACCTTACAGAAAAATTTTC	2160
347-10 LHA clone 1	--	1831
347-10 LHA clone 2	--	1831
347-10 LHA clone3	--	1831
354-3 LHA clone 1	--	1831
354-3 LHA clone 2	--	1831
354-3 LHA clone 3	--	1831
354-5_L5	--	1831
354-5_L6	--	1831
354-5_L8	--	1831
347-10_R5	CTAAATATTGTCCCGATTTGCGGTCACCAATTAATAGTGCGACCTTACAGAAAAATTTTC	979
347-10_R6	CTAAATATTGTCCCGATTTGCGGTCACCAATTAATAGTGCGACCTTACAGAAAAATTTTC	978
347-10_R7	CTAAATATTGTCCCGATTTGCGGTCACCAATTAATAGTGCGACCTTACAGAAAAATTTTC	978
354-3_R1	CTAAATATTGTCCCGATTTGCGGTCACCAATTAATAGTGCGACCTTACAGAAAAATTTTC	979
354-3_R2	CTAAATATTGTCCCGATTTGCGGTCACCAATTAATAGTGCGACCTTACAGAAAAATTTTC	979
354-3_R10	CTAAATATTGTCCCGATTTGCGGTCACCAATTAATAGTGCGACCTTACAGAAAAATTTTC	979
354-5_R9	CTAAATATTGTCCCGATTTGCGGTCACCAATTAATAGTGCGACCTTACAGAAAAATTTTC	979
354-5_R11	CTAAATATTGTCCCGATTTGCGGTCACCAATTAATAGTGCGACCTTACAGAAAAATTTTC	978
354-5_R12	CTAAATATTGTCCCGATTTGCGGTCACCAATTAATAGTGCGACCTTACAGAAAAATTTTC	979

	2,180 / 2,200 / 2,220	
expected sequence of Eef1a-FLAG-2A-eGFP allele	TGTCGCGCTAAATTGCAAATGCACCTGGATAAAAAAGCGCGAAGAAAATAAAGCGGAATT	2220
347-10 LHA clone 1	--	1831
347-10 LHA clone 2	--	1831
347-10 LHA clone3	--	1831
354-3 LHA clone 1	--	1831
354-3 LHA clone 2	--	1831
354-3 LHA clone 3	--	1831
354-5_L5	--	1831
354-5_L6	--	1831
354-5_L8	--	1831
347-10_R5	TGTCGCGCTAAATTGCAAATGCACCTGGATAAAAAAGCGCGAAGAAAATAAAGCGG----	1035
347-10_R6	TGTCGCGCTAAATTGCAAATGCACCTGGATAAAAAAGCGCGAAGAAAATAAAGCGG----	1034
347-10_R7	TGTCGCGCTAAATTGCAAATGCACCTGGATAAAAAAGCGCGAAGAAAATAAAGCGG----	1034
354-3_R1	TGTCGCGCTAAATTGCAAATGCACCTGGATAAAAAAGCGCGAAGAAAATAAAGCGG----	1035
354-3_R2	TGTCGCGCTAAATTGCAAATGCACCTGGATAAAAAAGCGCGAAGAAAATAAAGCGG----	1035
354-3_R10	TGTCGCGCTAAATTGCAAATGCACCTGGATAAAAAAGCGCGAAGAAAATAAAGCGG----	1035
354-5_R9	TGTCGCGCTAAATTGCAAATGCACCTGGATAAAAAAGCGCGAAGAAAATAAAGCGG----	1035
354-5_R11	TGTCGCGCTAAATTGCAAATGCACCTGGATAAAAAAGCGCGAAGAAAATAAAGCGG----	1034
354-5_R12	TGTCGCGCTAAATTGCAAATGCACCTGGATAAAAAAGCGCGAAGAAAATAAAGCGG----	1035

	2,240 · 2,260 · 2,280	
expected sequence of Eef1a-FLAG-2A-eGFP allele	AATGTTTGCGAGTGTACTTCGTATTCGCCCGAAGGTGTTCTATAAAATTTAACTAAGCCT	2280
347-10 LHA clone 1	--	1831
347-10 LHA clone 2	--	1831
347-10 LHA clone3	--	1831
354-3 LHA clone 1	--	1831
354-3 LHA clone 2	--	1831
354-3 LHA clone 3	--	1831
354-5_L5	--	1831
354-5_L6	--	1831
354-5_L8	--	1831
347-10_R5	--	1035
347-10_R6	--	1034
347-10_R7	--	1034
354-3_R1	--	1035
354-3_R2	--	1035
354-3_R10	--	1035
354-5_R9	--	1035
354-5_R11	--	1034
354-5_R12	--	1035

	2,300 · 2,320 · 2,340	
expected sequence of Eef1a-FLAG-2A-eGFP allele	TTTTTAAACAAAAACAGACGATATCAATCTTCTTGCATGATACAAAAAAATTTAAGCTTA	2340
347-10 LHA clone 1	--	1831
347-10 LHA clone 2	--	1831
347-10 LHA clone3	--	1831
354-3 LHA clone 1	--	1831
354-3 LHA clone 2	--	1831
354-3 LHA clone 3	--	1831
354-5_L5	--	1831
354-5_L6	--	1831
354-5_L8	--	1831
347-10_R5	--	1035
347-10_R6	--	1034
347-10_R7	--	1034
354-3_R1	--	1035
354-3_R2	--	1035
354-3_R10	--	1035
354-5_R9	--	1035
354-5_R11	--	1034
354-5_R12	--	1035

	2,360 · 2,380 · 2,400	
expected sequence of Eef1a-FLAG-2A-eGFP allele	GTGTAAATATGGCTTAATGTTTTTAAGTTCGAGTATTTTCAAGTCATTAAACGGTGTTTT	2400
347-10 LHA clone 1	--	1831
347-10 LHA clone 2	--	1831
347-10 LHA clone3	--	1831
354-3 LHA clone 1	--	1831
354-3 LHA clone 2	--	1831
354-3 LHA clone 3	--	1831
354-5_L5	--	1831
354-5_L6	--	1831
354-5_L8	--	1831
347-10_R5	--	1035
347-10_R6	--	1034
347-10_R7	--	1034
354-3_R1	--	1035
354-3_R2	--	1035
354-3_R10	--	1035
354-5_R9	--	1035
354-5_R11	--	1034
354-5_R12	--	1035

		2,420 · 2,440 · 2,460	
expected sequence of Eef1a-FLAG-2A-eGFP allele		ACTATGTGAATATCAACTGTTTGCTCACACAATTATCTTCAGTAATAATTTTTACTGCTT	2460
347-10 LHA clone 1		--	1831
347-10 LHA clone 2		--	1831
347-10 LHA clone3		--	1831
354-3 LHA clone 1		--	1831
354-3 LHA clone 2		--	1831
354-3 LHA clone 3		--	1831
354-5_L5		--	1831
354-5_L6		--	1831
354-5_L8		--	1831
347-10_R5		--	1035
347-10_R6		--	1034
347-10_R7		--	1034
354-3_R1		--	1035
354-3_R2		--	1035
354-3_R10		--	1035
354-5_R9		--	1035
354-5_R11		--	1034
354-5_R12		--	1035

		2,480 · 2,500 · 2,520	
expected sequence of Eef1a-FLAG-2A-eGFP allele		AATTATGTTTAAGTTGATGATGATTGCGGTACCGTGTTTGAGTAGCACATCTTGTCTAAT	2520
347-10 LHA clone 1		--	1831
347-10 LHA clone 2		--	1831
347-10 LHA clone3		--	1831
354-3 LHA clone 1		--	1831
354-3 LHA clone 2		--	1831
354-3 LHA clone 3		--	1831
354-5_L5		--	1831
354-5_L6		--	1831
354-5_L8		--	1831
347-10_R5		--	1035
347-10_R6		--	1034
347-10_R7		--	1034
354-3_R1		--	1035
354-3_R2		--	1035
354-3_R10		--	1035
354-5_R9		--	1035
354-5_R11		--	1034
354-5_R12		--	1035

		2,540 · 2,560 · 2,580	
expected sequence of Eef1a-FLAG-2A-eGFP allele		AACAGATACCATTTCTAATTGTATGGTAGGCATGCTACCGAACATCAAAACGTTGCTCAC	2580
347-10 LHA clone 1		--	1831
347-10 LHA clone 2		--	1831
347-10 LHA clone3		--	1831
354-3 LHA clone 1		--	1831
354-3 LHA clone 2		--	1831
354-3 LHA clone 3		--	1831
354-5_L5		--	1831
354-5_L6		--	1831
354-5_L8		--	1831
347-10_R5		--	1035
347-10_R6		--	1034
347-10_R7		--	1034
354-3_R1		--	1035
354-3_R2		--	1035
354-3_R10		--	1035
354-5_R9		--	1035
354-5_R11		--	1034
354-5_R12		--	1035

expected sequence of Eef1a-FLAG-2A-eGFP allele	ACGTCGGTTATCCAGTTACAAGGCAAGATGTACTAATCGGCCACGGTATTTCGGTATCCA	2640
347-10 LHA clone 1	--	1831
347-10 LHA clone 2	--	1831
347-10 LHA clone3	--	1831
354-3 LHA clone 1	--	1831
354-3 LHA clone 2	--	1831
354-3 LHA clone 3	--	1831
354-5_L5	--	1831
354-5_L6	--	1831
354-5_L8	--	1831
347-10_R5	--	1035
347-10_R6	--	1034
347-10_R7	--	1034
354-3_R1	--	1035
354-3_R2	--	1035
354-3_R10	--	1035
354-5_R9	--	1035
354-5_R11	--	1034
354-5_R12	--	1035

expected sequence of Eef1a-FLAG-2A-eGFP allele	AGAATAAAACGTGTGTGATTGCGTATATTAGCGAACATGTGCAACAAGATGTTAAAAACA	2700
347-10 LHA clone 1	--	1831
347-10 LHA clone 2	--	1831
347-10 LHA clone3	--	1831
354-3 LHA clone 1	--	1831
354-3 LHA clone 2	--	1831
354-3 LHA clone 3	--	1831
354-5_L5	--	1831
354-5_L6	--	1831
354-5_L8	--	1831
347-10_R5	--	1035
347-10_R6	--	1034
347-10_R7	--	1034
354-3_R1	--	1035
354-3_R2	--	1035
354-3_R10	--	1035
354-5_R9	--	1035
354-5_R11	--	1034
354-5_R12	--	1035

expected sequence of Eef1a-FLAG-2A-eGFP allele	CCTGTTAACTAATAGTCGTTACCATAGTGACTAATATTAATGCAAATGTAAAAGAATTCC	2760
347-10 LHA clone 1	--	1831
347-10 LHA clone 2	--	1831
347-10 LHA clone3	--	1831
354-3 LHA clone 1	--	1831
354-3 LHA clone 2	--	1831
354-3 LHA clone 3	--	1831
354-5_L5	--	1831
354-5_L6	--	1831
354-5_L8	--	1831
347-10_R5	--	1035
347-10_R6	--	1034
347-10_R7	--	1034
354-3_R1	--	1035
354-3_R2	--	1035
354-3_R10	--	1035
354-5_R9	--	1035
354-5_R11	--	1034
354-5_R12	--	1035

	2,780 · 2,800 · 2,820	
expected sequence of Eef1a-FLAG-2A-eGFP allele	GTAAAAAACTTTCAAAGTTCATTTTTAGGAGAGTTATTTGACAAAACATAATTTTTGCAA	2820
347-10 LHA clone 1	--	1831
347-10 LHA clone 2	--	1831
347-10 LHA clone3	--	1831
354-3 LHA clone 1	--	1831
354-3 LHA clone 2	--	1831
354-3 LHA clone 3	--	1831
354-5_L5	--	1831
354-5_L6	--	1831
354-5_L8	--	1831
347-10_R5	--	1035
347-10_R6	--	1034
347-10_R7	--	1034
354-3_R1	--	1035
354-3_R2	--	1035
354-3_R10	--	1035
354-5_R9	--	1035
354-5_R11	--	1034
354-5_R12	--	1035

	2,840 · 2,860 · 2,880	
expected sequence of Eef1a-FLAG-2A-eGFP allele	TTAATTATTCTTACGCTTCATTGAAGATCATACTTAATAATTTTTTTAATAGTAATTATT	2880
347-10 LHA clone 1	--	1831
347-10 LHA clone 2	--	1831
347-10 LHA clone3	--	1831
354-3 LHA clone 1	--	1831
354-3 LHA clone 2	--	1831
354-3 LHA clone 3	--	1831
354-5_L5	--	1831
354-5_L6	--	1831
354-5_L8	--	1831
347-10_R5	--	1035
347-10_R6	--	1034
347-10_R7	--	1034
354-3_R1	--	1035
354-3_R2	--	1035
354-3_R10	--	1035
354-5_R9	--	1035
354-5_R11	--	1034
354-5_R12	--	1035

	2,900 · 2,920 · 2,940	
expected sequence of Eef1a-FLAG-2A-eGFP allele	AGTTATTCTTGAAAATTGGTGTGGACACTCAAAACGCGTCGAATGCCCCTCTATTTTTGC	2940
347-10 LHA clone 1	--	1831
347-10 LHA clone 2	--	1831
347-10 LHA clone3	--	1831
354-3 LHA clone 1	--	1831
354-3 LHA clone 2	--	1831
354-3 LHA clone 3	--	1831
354-5_L5	--	1831
354-5_L6	--	1831
354-5_L8	--	1831
347-10_R5	------------------GTGTGGACACTCAAAACGCGTCGAATGCCCCTCTATTTTTGC	1077
347-10_R6	------------------GTGTGGACACTCAAAACGCGTCGAATGCCCCTCTATTTTTGC	1076
347-10_R7	------------------GTGTGGACACTCAAAACGCGTCGAATGCCCCTCTATTTTTGC	1076
354-3_R1	------------------GTGTGGACACTCAAAACGCGTCGAATGCCCCTCTATTTTTGC	1077
354-3_R2	------------------GTGTGGACACTCAAAACGCGTCGAATGCCCCTCTATTTTTGC	1077
354-3_R10	------------------GTGTGGACACTCAAAACGCGTCGAATGCCCCTCTATTTTTGC	1077
354-5_R9	------------------GTGTGGACACTCAAAACGCGTCGAATGCCCCTCTATTTTTGC	1077
354-5_R11	------------------GTGTGGACACTCAAAACGCGTCGAATGCCCCTCTATTTTTGC	1076
354-5_R12	------------------GTGTGGACACTCAAAACGCGTCGAATGCCCCTCTATTTTTGC	1077

```
                                                                             2,960                      2,980                      3,000
expected sequence of Eef1a-FLAG-2A-eGFP allele  TGATACGTCAGCGAGTGTGTCATTCTGGCCGCTAGCAGTTGAATTTTTGAACGTTTCACC 3000
347-10 LHA clone 1                              ------------------------------------------------------------ 1831
347-10 LHA clone 2                              ------------------------------------------------------------ 1831
347-10 LHA clone3                               ------------------------------------------------------------ 1831
354-3 LHA clone 1                               ------------------------------------------------------------ 1831
354-3 LHA clone 2                               ------------------------------------------------------------ 1831
354-3 LHA clone 3                               ------------------------------------------------------------ 1831
354-5_L5                                        ------------------------------------------------------------ 1831
354-5_L6                                        ------------------------------------------------------------ 1831
354-5_L8                                        ------------------------------------------------------------ 1831
347-10_R5                                       TGATACGTCAGCGAGTGTGTCATTCTGGCCGCTAGCAGTTGAATTTTTGAACGTTTCACC 1137
347-10_R6                                       TGATACGTCAGCGAGTGTGTCATTCTGGCCGCTAGCAGTTGAATTTTTGAACGTTTCACC 1136
347-10_R7                                       TGATACGTCAGCGAGTGTGTCATTCTGGCCGCTAGCAGTTGAATTTTTGAACGTTTCACC 1136
354-3_R1                                        TGATACGTCAGCGAGTGTGTCATTCTGGCCGCTAGCAGTTGAATTTTTGAACGTTTCACC 1137
354-3_R2                                        TGATACGTCAGCGAGTGTGTCATTCTGGCCGCTAGCAGTTGAATTTTTGAACGTTTCACC 1137
354-3_R10                                       TGATACGTCAGCGAGTGTGTCATTCTGGCCGCTAGCAGTTGAATTTTTGAACGTTTCACC 1137
354-5_R9                                        TGATACGTCAGCGAGTGTGTCATTCTGGCCGCTAGCAGTTGAATTTTTGAACGTTTCACC 1137
354-5_R11                                       TGATACGTCAGCGAGTGTGTCATTCTGGCCGCTAGCAGTTGAATTTTTGAACGTTTCACC 1136
354-5_R12                                       TGATACGTCAGCGAGTGTGTCATTCTGGCCGCTAGCAGTTGAATTTTTGAACGTTTCACC 1137
```

```
                                                                             3,020                      3,040                      3,060
expected sequence of Eef1a-FLAG-2A-eGFP allele  AGAAAACTAAGTCAGCATTGTTACTTACGTCACTATTGAACCAATAATGACTCATCTCTG 3060
347-10 LHA clone 1                              ------------------------------------------------------------ 1831
347-10 LHA clone 2                              ------------------------------------------------------------ 1831
347-10 LHA clone3                               ------------------------------------------------------------ 1831
354-3 LHA clone 1                               ------------------------------------------------------------ 1831
354-3 LHA clone 2                               ------------------------------------------------------------ 1831
354-3 LHA clone 3                               ------------------------------------------------------------ 1831
354-5_L5                                        ------------------------------------------------------------ 1831
354-5_L6                                        ------------------------------------------------------------ 1831
354-5_L8                                        ------------------------------------------------------------ 1831
347-10_R5                                       AGAAAACTAAGTCAGCATTGTTACTTACGTCACTATTGAACCAATAATGACTCATCTCTG 1197
347-10_R6                                       AGAAAACTAAGTCAGCATTGTTACTTACGTCACTATTGAACCAATAATGACTCATCTCTG 1196
347-10_R7                                       AGAAAACTAAGTCAGCATTGTTACTTACGTCACTATTGAACCAATAATGACTCATCTCTG 1196
354-3_R1                                        AGAAAACTAAGTCAGCATTGTTACTTACGTCACTATTGAACCAATAATGACTCATCTCTG 1197
354-3_R2                                        AGAAAACTAAGTCAGCATTGTTACTTACGTCACTATTGAACCAATAATGACTCATCTCTG 1197
354-3_R10                                       AGAAAACTAAGTCAGCATTGTTACTTACGTCACTATTGAACCAATAATGACTCATCTCTG 1197
354-5_R9                                        AGAAAACTAAGTCAGCATTGTTACTTACGTCACTATTGAACCAATAATGACTCATCTCTG 1197
354-5_R11                                       AGAAAACTAAGTCAGCATTGTTACTTACGTCACTATTGAACCAATAATGACTCATCTCTG 1196
354-5_R12                                       AGAAAACTAAGTCAGCATTGTTACTTACGTCACTATTGAACCAATAATGACTCATCTCTG 1197
```

```
                                                                                                    Pr877
                                                                             3,080                      3,100
expected sequence of Eef1a-FLAG-2A-eGFP allele  CTAACTTAGTACATAATTCATCCAAAATTTCGGCAGCGATGTTGCGTTTATTATTG 3116
347-10 LHA clone 1                              -------------------------------------------------------- 1831
347-10 LHA clone 2                              -------------------------------------------------------- 1831
347-10 LHA clone3                               -------------------------------------------------------- 1831
354-3 LHA clone 1                               -------------------------------------------------------- 1831
354-3 LHA clone 2                               -------------------------------------------------------- 1831
354-3 LHA clone 3                               -------------------------------------------------------- 1831
354-5_L5                                        -------------------------------------------------------- 1831
354-5_L6                                        -------------------------------------------------------- 1831
354-5_L8                                        -------------------------------------------------------- 1831
347-10_R5                                       CTAACTTAGTACATAATTCATCCAAAATTTCGGCAGCGATGTTGCGTTTATTATTG 1253
347-10_R6                                       CTAACTTAGTACATAATTCATCCAAAATTTCGGCAGCGATGTTGCGTTTATTATTG 1252
347-10_R7                                       CTAACTTAGTACATAATTCATCCAAAATTTCGGCAGCGATGTTGCGTTTATTATTG 1252
354-3_R1                                        CTAACTTAGTACATAATTCATCCAAAATTTCGGCAGCGATGTTGCGTTTATTATTG 1253
354-3_R2                                        CTAACTTAGTACATAATTCATCCAAAATTTCGGCAGCGATGTTGCGTTTATTATTG 1253
354-3_R10                                       CTAACTTAGTACATAATTCATCCAAAATTTCGGCAGCGATGTTGCGTTTATTATTG 1253
354-5_R9                                        CTAACTTAGTACATAATTCATCCAAAATTTCGGCAGCGATGTTGCGTTTATTATTG 1253
354-5_R11                                       CTAACTTAGTACATAATTCATCCAAAATTTCGGCAGCGATGTTGCGTTTATTATTG 1252
354-5_R12                                       CTAACTTAGTACATAATTCATCCAAAATTTCGGCAGCGATGTTGCGTTTATTATTG 1253
```

S5 Fig. Alignment of upstream and downstream integration sites from *Eef1a*$^{FLAG\text{-}P2A\text{-}eGFP}$ colonies. Nucleotide alignment of the regions encoding the up- and downstream integration sites from the founder colony, 347-10, and two transgenic offspring, 354-3 and 354-5. Three cloned amplicons were sequenced for each animal at each site. Primer binding sites are indicated in green on the expected sequence. Differences are highlighted in red boxes. Note that the regions that do not overlap between the upstream and downstream sequences are shown as gaps at the beginning and end of the alignment.
