# Supplementary material for: CRISPR/Cas9-mediated gene knockin in the hydroid Hydractinia symbiolongicarpus
Source: BMC Genomics. 2018 Sep 3;19:649. doi: 10.1186/s12864-018-5032-z (PMC6122657; doi:10.1186/s12864-018-5032-z)
Supplement: Supplementary file 19 — Alignment of upstream and downstream integration sites from Eef1aSF-P2A-tdTom colonies. Nucleotide alignment of the region encoding the upstream integration site from the founder colony, 350–10, and one transgenic offspring, 355–8. Three cloned amplicons were sequenced for each animal at each site. Primer binding sites are indicated in green on the expected sequence. (PDF 3670 kb) [file 12864_2018_5032_MOESM19_ESM.pdf]

```
                                        Pr845
                                        20                  40                  60
expected_sequence_EF1a-SF-P2A-tdTom  GTAGAAACTGGTGTATTGTCCCCTGGTATGATCGTTACATTTGCCCCTGCCAATGTAACC 60
350-10_c2                            GTAGAAACTGGTGTATTGTCCCCTGGTATGATCGTTACATTTGCCCCTGCCAATGTAACC 60
350-10_c3                            GTAGAAACTGGTGTATTGTCCCCTGGTATGATCGTTACATTTGCCCCTGCCAATGTAACC 60
350-10_c5                            GTAGAAACTGGTGTATTGTCCCCTGGTATGATCGTTACATTTGCCCCTGCCAATGTAACC 60
355-8_c11                            GTAGAAACTGGTGTATTGTCCCCTGGTATGATCGTTACATTTGCCCCTGCCAATGTAACC 60
355-8_c13                            GTAGAAACTGGTGTATTGTCCCCTGGTATGATCGTTACATTTGCCCCTGCCAATGTAACC 60
355-8_c14                            GTAGAAACTGGTGTATTGTCCCCTGGTATGATCGTTACATTTGCCCCTGCCAATGTAACC 60
```

```
                                        80                  100                 120
expected_sequence_EF1a-SF-P2A-tdTom  ACTGAGGTCAAATCTGTAGAAATGCACCACGAATCCTTAACTGAAGCACATCCAGGTGAT 120
350-10_c2                            ACTGAGGTCAAATCTGTAGAAATGCACCACGAATCCTTAACTGAAGCACATCCAGGTGAT 120
350-10_c3                            ACTGAGGTCAAATCTGTAGAAATGCACCACGAATCCTTAACTGAAGCACATCCAGGTGAT 120
350-10_c5                            ACTGAGGTCAAATCTGTAGAAATGCACCACGAATCCTTAACTGAAGCACATCCAGGTGAT 120
355-8_c11                            ACTGAGGTCAAATCTGTAGAAATGCACCACGAATCCTTAACTGAAGCACATCCAGGTGAT 120
355-8_c13                            ACTGAGGTCAAATCTGTAGAAATGCACCACGAATCCTTAACTGAAGCACATCCAGGTGAT 120
355-8_c14                            ACTGAGGTCAAATCTGTAGAAATGCACCACGAATCCTTAACTGAAGCACATCCAGGTGAT 120
```

```
                                        140                 160                 180
expected_sequence_EF1a-SF-P2A-tdTom  AATGTTGGTTTTAATGTCAAAAATGTGTCTATCAAGGATATCAAACGTGGTATGGTTGCG 180
350-10_c2                            AATGTTGGTTTTAATGTCAAAAATGTGTCTATCAAGGATATCAAACGTGGTATGGTTGCG 180
350-10_c3                            AATGTTGGTTTTAATGTCAAAAATGTGTCTATCAAGGATATCAAACGTGGTATGGTTGCG 180
350-10_c5                            AATGTTGGTTTTAATGTCAAAAATGTGTCTATCAAGGATATCAAACGTGGTATGGTTGCG 180
355-8_c11                            AATGTTGGTTTTAATGTCAAAAATGTGTCTATCAAGGATATCAAACGTGGTATGGTTGCG 180
355-8_c13                            AATGTTGGTTTTAATGTCAAAAATGTGTCTATCAAGGATATCAAACGTGGTATGGTTGCG 180
355-8_c14                            AATGTTGGTTTTAATGTCAAAAATGTGTCTATCAAGGATATCAAACGTGGTATGGTTGCG 180
```

```
                                        200                 220                 240
expected_sequence_EF1a-SF-P2A-tdTom  TCTGACAGCAAAAATGATCCAGCCAAAGAAGCCAAAACCTTTTATGCACAGGTATTCTTT 240
350-10_c2                            TCTGACAGCAAAAATGATCCAGCCAAAGAAGCCAAAACCTTTTATGCACAGGTATTCTTT 240
350-10_c3                            TCTGACAGCAAAAATGATCCAGCCAAAGAAGCCAAAACCTTTTATGCACAGGTATTCTTT 240
350-10_c5                            TCTGACAGCAAAAATGATCCAGCCAAAGAAGCCAAAACCTTTTATGCACAGGTATTCTTT 240
355-8_c11                            TCTGACAGCAAAAATGATCCAGCCAAAGAAGCCAAAACCTTTTATGCACAGGTATTCTTT 240
355-8_c13                            TCTGACAGCAAAAATGATCCAGCCAAAGAAGCCAAAACCTTTTATGCACAGGTATTCTTT 240
355-8_c14                            TCTGACAGCAAAAATGATCCAGCCAAAGAAGCCAAAACCTTTTATGCACAGGTATTCTTT 240
```

```
                                        260                 280                 300
expected_sequence_EF1a-SF-P2A-tdTom  AATTTTTGTACTTAACTTATCAGTTTGTATAAGCGTGTTGGCCGTGGCTTGCCACTTGCT 300
350-10_c2                            AATTTTTGTACTTAACTTATCAGTTTGTATAAGCGTGTTGGCCGTGGCTTGCCACTTGCT 300
350-10_c3                            AATTTTTGTACTTAACTTATCAGTTTGTATAAGCGTGTTGGCCGTGGCTTGCCACTTGCT 300
350-10_c5                            AATTTTTGTACTTAACTTATCAGTTTGTATAAGCGTGTTGGCCGTGGCTTGCCACTTGCT 300
355-8_c11                            AATTTTTGTACTTAACTTATCAGTTTGTATAAGCGTGTTGGCCGTGGCTTGCCACTTGCT 300
355-8_c13                            AATTTTTGTACTTAACTTATCAGTTTGTATAAGCGTGTTGGCCGTGGCTTGCCACTTGCT 300
355-8_c14                            AATTTTTGTACTTAACTTATCAGTTTGTATAAGCGTGTTGGCCGTGGCTTGCCACTTGCT 300
```

```
                                        320                 340                 360
expected_sequence_EF1a-SF-P2A-tdTom  TAGCATGATAAATCAATCAACTTGACATTCCATCAATTTTGTTCAACAGAAACAGGACCT 360
350-10_c2                            TAGCATGATAAATCAATCAACTTGACATTCCATCAATTTTGTTCAACAGAAACAGGACCT 360
350-10_c3                            TAGCATGATAAATCAATCAACTTGACATTCCATCAATTTTGTTCAACAGAAACAGGACCT 360
350-10_c5                            TAGCATGATAAATCAATCAACTTGACATTCCATCAATTTTGTTCAACAGAAACAGGACCT 360
355-8_c11                            TAGCATGATAAATCAATCAACTTGACATTCCATCAATTTTGTTCAACAGAAACAGGACCT 360
355-8_c13                            TAGCATGATAAATCAATCAACTTGACATTCCATCAATTTTGTTCAACAGAAACAGGACCT 360
355-8_c14                            TAGCATGATAAATCAATCAACTTGACATTCCATCAATTTTGTTCAACAGAAACAGGACCT 360
```

```
                                        380                 400                 420
expected_sequence_EF1a-SF-P2A-tdTom  TTAAATAGCAAACAAAATATTGAAATAAAACTGTATGATTTTGTCACTATCGTTTATGTG 420
350-10_c2                            TTAAATAGCAAACAAAATATTGAAATAAAACTGTATGATTTTGTCACTATCGTTTATGTG 420
350-10_c3                            TTAAATAGCAAACAAAATATTGAAATAAAACTGTATGATTTTGTCACTATCGTTTATGTG 420
350-10_c5                            TTAAATAGCAAACAAAATATTGAAATAAAACTGTATGATTTTGTCACTATCGTTTATGTG 420
355-8_c11                            TTAAATAGCAAACAAAATATTGAAATAAAACTGTATGATTTTGTCACTATCGTTTATGTG 420
355-8_c13                            TTAAATAGCAAACAAAATATTGAAATAAAACTGTATGATTTTGTCACTATCGTTTATGTG 420
355-8_c14                            TTAAATAGCAAACAAAATATTGAAATAAAACTGTATGATTTTGTCACTATCGTTTATGTG 420
```

```
                                                                 440                         460                         480
                                                                 |                           |                           |
expected_sequence_EF1a-SF-P2A-tdTom  CCCACCATGAAAATTTTACAAAGAAGTGGAACTGTTTGAAAGTTCATTAGCAAGAACAAA 480
                         350-10_c2  CCCACCATGAAAATTTTACAAAGAAGTGGAACTGTTTGAAAGTTCATTAGCAAGAACAAA 480
                         350-10_c3  CCCACCATGAAAATTTTACAAAGAAGTGGAACTGTTTGAAAGTTCATTAGCAAGAACAAA 480
                         350-10_c5  CCCACCATGAAAATTTTACAAAGAAGTGGAACTGTTTGAAAGTTCATTAGCAAGAACAAA 480
                         355-8_c11  CCCACCATGAAAATTTTACAAAGAAGTGGAACTGTTTGAAAGTTCATTAGCAAGAACAAA 480
                         355-8_c13  CCCACCATGAAAATTTTACAAAGAAGTGGAACTGTTTGAAAGTTCATTAGCAAGAACAAA 480
                         355-8_c14  CCCACCATGAAAATTTTACAAAGAAGTGGAACTGTTTGAAAGTTCATTAGCAAGAACAAA 480
                                                                 500                         520                         540
                                                                 |                           |                           |
expected_sequence_EF1a-SF-P2A-tdTom  TCCTAGAAATGTTAGATTTGTGTCAAGAATTTCAAGATTTTTGTTTCTTGGCAAGTTTTT 540
                         350-10_c2  TCCTAGAAATGTTAGATTTGTGTCAAGAATTTCAAGATTTTTGTTTCTTGGCAAGTTTTT 540
                         350-10_c3  TCCTAGAAATGTTAGATTTGTGTCAAGAATTTCAAGATTTTTGTTTCTTGGCAAGTTTTT 540
                         350-10_c5  TCCTAGAAATGTTAGATTTGTGTCAAGAATTTCAAGATTTTTGTTTCTTGGCAAGTTTTT 540
                         355-8_c11  TCCTAGAAATGTTAGATTTGTGTCAAGAATTTCAAGATTTTTGTTTCTTGGCAAGTTTTT 540
                         355-8_c13  TCCTAGAAATGTTAGATTTGTGTCAAGAATTTCAAGATTTTTGTTTCTTGGCAAGTTTTT 540
                         355-8_c14  TCCTAGAAATGTTAGATTTGTGTCAAGAATTTCAAGATTTTTGTTTCTTGGCAAGTTTTT 540
                                                                 560                         580                         600
                                                                 |                           |                           |
expected_sequence_EF1a-SF-P2A-tdTom  AGCACTTGCTTAGGGAACTATTTAAAATATTAATTTTTATTGAATAAAGAACCAACCTGT 600
                         350-10_c2  AGCACTTGCTTAGGGAACTATTTAAAATATTAATTTTTATTGAATAAAGAACCAACCTGT 600
                         350-10_c3  AGCACTTGCTTAGGGAACTATTTAAAATATTAATTTTTATTGAATAAAGAACCAACCTGT 600
                         350-10_c5  AGCACTTGCTTAGGGAACTATTTAAAATATTAATTTTTATTGAATAAAGAACCAACCTGT 600
                         355-8_c11  AGCACTTGCTTAGGGAACTATTTAAAATATTAATTTTTATTGAATAAAGAACCAACCTGT 600
                         355-8_c13  AGCACTTGCTTAGGGAACTATTTAAAATATTAATTTTTATTGAATAAAGAACCAACCTGT 600
                         355-8_c14  AGCACTTGCTTAGGGAACTATTTAAAATATTAATTTTTATTGAATAAAGAACCAACCTGT 600
                                                                 620                         640                         660
                                                                 |                           |                           |
expected_sequence_EF1a-SF-P2A-tdTom  TTGACTTTTATTAGTAATAAAGCTAAAATTATGGCCTAAATCATAATTTATTATGTGATT 660
                         350-10_c2  TTGACTTTTATTAGTAATAAAGCTAAAATTATGGCCTAAATCATAATTTATTATGTGATT 660
                         350-10_c3  TTGACTTTTATTAGTAATAAAGCTAAAATTATGGCCTAAATCATAATTTATTATGTGATT 660
                         350-10_c5  TTGACTTTTATTAGTAATAAAGCTAAAATTATGGCCTAAATCATAATTTATTATGTGATT 660
                         355-8_c11  TTGACTTTTATTAGTAATAAAGCTAAAATTATGGCCTAAATCATAATTTATTATGTGATT 660
                         355-8_c13  TTGACTTTTATTAGTAATAAAGCTAAAATTATGGCCTAAATCATAATTTATTATGTGATT 660
                         355-8_c14  TTGACTTTTATTAGTAATAAAGCTAAAATTATGGCCTAAATCATAATTTATTATGTGATT 660
                                                                 680                         700                         720
                                                                 |                           |                           |
expected_sequence_EF1a-SF-P2A-tdTom  TGGGAAAGAAATTTGTGTTGATTTTTCCATTAATATTGTAAAAACAAAATCGAAAGTTAA 720
                         350-10_c2  TGGGAAAGAAATTTGTGTTGATTTTTCCATTAATATTGTAAAAACAAAATCGAAAGTTAA 720
                         350-10_c3  TGGGAAAGAAATTTGTGTTGATTTTTCCATTAATATTGTAAAAACAAAATCGAAAGTTAA 720
                         350-10_c5  TGGGAAAGAAATTTGTGTTGATTTTTCCATTAATATTGTAAAAACAAAATCGAAAGTTAA 720
                         355-8_c11  TGGGAAAGAAATTTGTGTTGATTTTTCCATTAATATTGTAAAAACAAAATCGAAAGTTAA 720
                         355-8_c13  TGGGAAAGAAATTTGTGTTGATTTTTCCATTAATATTGTAAAAACAAAATCGAAAGTTAA 720
                         355-8_c14  TGGGAAAGAAATTTGTGTTGATTTTTCCATTAATATTGTAAAAACAAAATCGAAAGTTAA 720
                                                                 740                         760                         780
                                                                 |                           |                           |
expected_sequence_EF1a-SF-P2A-tdTom  TTTCTTTCAAAAATTACTTTTAGGTCATCATTCTCAATCATCCAGGTGAGATTCATGCTG 780
                         350-10_c2  TTTCTTTCAAAAATTACTTTTAGGTCATCATTCTCAATCATCCAGGTGAGATTCATGCTG 780
                         350-10_c3  TTTCTTTCAAAAATTACTTTTAGGTCATCATTCTCAATCATCCAGGTGAGATTCATGCTG 780
                         350-10_c5  TTTCTTTCAAAAATTACTTTTAGGTCATCATTCTCAATCATCCAGGTGAGATTCATGCTG 780
                         355-8_c11  TTTCTTTCAAAAATTACTTTTAGGTCATCATTCTCAATCATCCAGGTGAGATTCATGCTG 780
                         355-8_c13  TTTCTTTCAAAAATTACTTTTAGGTCATCATTCTCAATCATCCAGGTGAGATTCATGCTG 780
                         355-8_c14  TTTCTTTCAAAAATTACTTTTAGGTCATCATTCTCAATCATCCAGGTGAGATTCATGCTG 780
                                                                 800                         820                         840
                                                                 |                           |                           |
expected_sequence_EF1a-SF-P2A-tdTom  GTTACCAACCTGTTTTGGATTGCCACACTGCTCACGTTGCCTGCAAATTTACAGAATTAA 840
                         350-10_c2  GTTACCAACCTGTTTTGGATTGCCACACTGCTCACGTTGCCTGCAAATTTACAGAATTAA 840
                         350-10_c3  GTTACCAACCTGTTTTGGATTGCCACACTGCTCACGTTGCCTGCAAATTTACAGAATTAA 840
                         350-10_c5  GTTACCAACCTGTTTTGGATTGCCACACTGCTCACGTTGCCTGCAAATTTACAGAATTAA 840
                         355-8_c11  GTTACCAACCTGTTTTGGATTGCCACACTGCTCACGTTGCCTGCAAATTTACAGAATTAA 840
                         355-8_c13  GTTACCAACCTGTTTTGGATTGCCACACTGCTCACGTTGCCTGCAAATTTACAGAATTAA 840
                         355-8_c14  GTTACCAACCTGTTTTGGATTGCCACACTGCTCACGTTGCCTGCAAATTTACAGAATTAA 840
                                                                 860                         880                         900
                                                                 |                           |                           |
expected_sequence_EF1a-SF-P2A-tdTom  AGCAGAAATGTGATAGACGAAGTGGTAAAGTTTTAGAAGAAAATCCCAAAATGGTTAAAT 900
                         350-10_c2  AGCAGAAATGTGATAGACGAAGTGGTAAAGTTTTAGAAGAAAATCCCAAAATGGTTAAAT 900
                         350-10_c3  AGCAGAAATGTGATAGACGAAGTGGTAAAGTTTTAGAAGAAAATCCCAAAATGGTTAAAT 900
                         350-10_c5  AGCAGAAATGTGATAGACGAAGTGGTAAAGTTTTAGAAGAAAATCCCAAAATGGTTAAAT 900
                         355-8_c11  AGCAGAAATGTGATAGACGAAGTGGTAAAGTTTTAGAAGAAAATCCCAAAATGGTTAAAT 900
                         355-8_c13  AGCAGAAATGTGATAGACGAAGTGGTAAAGTTTTAGAAGAAAATCCCAAAATGGTTAAAT 900
                         355-8_c14  AGCAGAAATGTGATAGACGAAGTGGTAAAGTTTTAGAAGAAAATCCCAAAATGGTTAAAT 900
```

Supporting Figure S7

	920 … 940 … 960	
expected_sequence_EF1a-SF-P2A-tdTom	CTGGTGATGCTGCTATGGTTACTTTGACTCCTTCTAAGCCTATGTGTGTTGAAGCTTTCT	960
350-10_c2	CTGGTGATGCTGCTATGGTTACTTTGACTCCTTCTAAGCCTATGTGTGTTGAAGCTTTCT	960
350-10_c3	CTGGTGATGCTGCTATGGTTACTTTGACTCCTTCTAAGCCTATGTGTGTTGAAGCTTTCT	960
350-10_c5	CTGGTGATGCTGCTATGGTTACTTTGACTCCTTCTAAGCCTATGTGTGTTGAAGCTTTCT	960
355-8_c11	CTGGTGATGCTGCTATGGTTACTTTGACTCCTTCTAAGCCTATGTGTGTTGAAGCTTTCT	960
355-8_c13	CTGGTGATGCTGCTATGGTTACTTTGACTCCTTCTAAGCCTATGTGTGTTGAAGCTTTCT	960
355-8_c14	CTGGTGATGCTGCTATGGTTACTTTGACTCCTTCTAAGCCTATGTGTGTTGAAGCTTTCT	960

	980 … 1,000 … 1,020	
expected_sequence_EF1a-SF-P2A-tdTom	CTGACTACCAGCCATTGGGTCGTTTTGCTGTACGTGACATGAGACAAACTGTTGCTGTTG	1020
350-10_c2	CTGACTACCAGCCATTGGGTCGTTTTGCTGTACGTGACATGAGACAAACTGTTGCTGTTG	1020
350-10_c3	CTGACTACCAGCCATTGGGTCGTTTTGCTGTACGTGACATGAGACAAACTGTTGCTGTTG	1020
350-10_c5	CTGACTACCAGCCATTGGGTCGTTTTGCTGTACGTGACATGAGACAAACTGTTGCTGTTG	1020
355-8_c11	CTGACTACCAGCCATTGGGTCGTTTTGCTGTACGTGACATGAGACAAACTGTTGCTGTTG	1020
355-8_c13	CTGACTACCAGCCATTGGGTCGTTTTGCTGTACGTGACATGAGACAAACTGTTGCTGTTG	1020
355-8_c14	CTGACTACCAGCCATTGGGTCGTTTTGCTGTACGTGACATGAGACAAACTGTTGCTGTTG	1020

	1,040 … 1,060 … 1,080	
expected_sequence_EF1a-SF-P2A-tdTom	GAGTTATCAAGAGTGTTGAAAAGGCCGATGCTACTGGTAAAATGACAAAATCAGCTCAAA	1080
350-10_c2	GAGTTATCAAGAGTGTTGAAAAGGCCGATGCTACTGGTAAAATGACAAAATCAGCTCAAA	1080
350-10_c3	GAGTTATCAAGAGTGTTGAAAAGGCCGATGCTACTGGTAAAATGACAAAATCAGCTCAAA	1080
350-10_c5	GAGTTATCAAGAGTGTTGAAAAGGCCGATGCTACTGGTAAAATGACAAAATCAGCTCAAA	1080
355-8_c11	GAGTTATCAAGAGTGTTGAAAAGGCCGATGCTACTGGTAAAATGACAAAATCAGCTCAAA	1080
355-8_c13	GAGTTATCAAGAGTGTTGAAAAGGCCGATGCTACTGGTAAAATGACAAAATCAGCTCAAA	1080
355-8_c14	GAGTTATCAAGAGTGTTGAAAAGGCCGATGCTACTGGTAAAATGACAAAATCAGCTCAAA	1080

	1,100 … 1,120 … 1,140	
expected_sequence_EF1a-SF-P2A-tdTom	AGGCTACCGGAAAGAAAAAATGGTCACATCCACAATTTGAAAAAGGTGGAGGTTCAGGAG	1140
350-10_c2	AGGCTACCGGAAAGAAAAAATGGTCACATCCACAATTTGAAAAAGGTGGAGGTTCAGGAG	1140
350-10_c3	AGGCTACCGGAAAGAAAAAATGGTCACATCCACAATTTGAAAAAGGTGGAGGTTCAGGAG	1140
350-10_c5	AGGCTACCGGAAAGAAAAAATGGTCACATCCACAATTTGAAAAAGGTGGAGGTTCAGGAG	1140
355-8_c11	AGGCTACCGGAAAGAAAAAATGGTCACATCCACAATTTGAAAAAGGTGGAGGTTCAGGAG	1140
355-8_c13	AGGCTACCGGAAAGAAAAAATGGTCACATCCACAATTTGAAAAAGGTGGAGGTTCAGGAG	1140
355-8_c14	AGGCTACCGGAAAGAAAAAATGGTCACATCCACAATTTGAAAAAGGTGGAGGTTCAGGAG	1140

	1,160 … 1,180 … 1,200	
expected_sequence_EF1a-SF-P2A-tdTom	GTGGATCAGGTGGAGGTTCATGGAGCCATCCACAATTTGAAAAAGGTGCTAGTGGAGAAG	1200
350-10_c2	GTGGATCAGGTGGAGGTTCATGGAGCCATCCACAATTTGAAAAAGGTGCTAGTGGAGAAG	1200
350-10_c3	GTGGATCAGGTGGAGGTTCATGGAGCCATCCACAATTTGAAAAAGGTGCTAGTGGAGAAG	1200
350-10_c5	GTGGATCAGGTGGAGGTTCATGGAGCCATCCACAATTTGAAAAAGGTGCTAGTGGAGAAG	1200
355-8_c11	GTGGATCAGGTGGAGGTTCATGGAGCCATCCACAATTTGAAAAAGGTGCTAGTGGAGAAG	1200
355-8_c13	GTGGATCAGGTGGAGGTTCATGGAGCCATCCACAATTTGAAAAAGGTGCTAGTGGAGAAG	1200
355-8_c14	GTGGATCAGGTGGAGGTTCATGGAGCCATCCACAATTTGAAAAAGGTGCTAGTGGAGAAG	1200

	1,220 … 1,240 … 1,260	
expected_sequence_EF1a-SF-P2A-tdTom	ATTATAAAGATGATGATGATAAAGGTTCAGGTGCTACAAATTTTTCATTATTAAAACAAG	1260
350-10_c2	ATTATAAAGATGATGATGATAAAGGTTCAGGTGCTACAAATTTTTCATTATTAAAACAAG	1260
350-10_c3	ATTATAAAGATGATGATGATAAAGGTTCAGGTGCTACAAATTTTTCATTATTAAAACAAG	1260
350-10_c5	ATTATAAAGATGATGATGATAAAGGTTCAGGTGCTACAAATTTTTCATTATTAAAACAAG	1260
355-8_c11	ATTATAAAGATGATGATGATAAAGGTTCAGGTGCTACAAATTTTTCATTATTAAAACAAG	1260
355-8_c13	ATTATAAAGATGATGATGATAAAGGTTCAGGTGCTACAAATTTTTCATTATTAAAACAAG	1260
355-8_c14	ATTATAAAGATGATGATGATAAAGGTTCAGGTGCTACAAATTTTTCATTATTAAAACAAG	1260

	1,280 … 1,300 … 1,320	
expected_sequence_EF1a-SF-P2A-tdTom	CTGGTGATGTTGAAGAAAATCCAGGTCCAATGACTTCCAAAGGTGAAGAGGTTATCAAAG	1320
350-10_c2	CTGGTGATGTTGAAGAAAATCCAGGTCCAATGACTTCCAAAGGTGAAGAGGTTATCAAAG	1320
350-10_c3	CTGGTGATGTTGAAGAAAATCCAGGTCCAATGACTTCCAAAGGTGAAGAGGTTATCAAAG	1320
350-10_c5	CTGGTGATGTTGAAGAAAATCCAGGTCCAATGACTTCCAAAGGTGAAGAGGTTATCAAAG	1320
355-8_c11	CTGGTGATGTTGAAGAAAATCCAGGTCCAATGACTTCCAAAGGTGAAGAGGTTATCAAAG	1320
355-8_c13	CTGGTGATGTTGAAGAAAATCCAGGTCCAATGACTTCCAAAGGTGAAGAGGTTATCAAAG	1320
355-8_c14	CTGGTGATGTTGAAGAAAATCCAGGTCCAATGACTTCCAAAGGTGAAGAGGTTATCAAAG	1320

	1,340 … 1,360 … 1,380	
expected_sequence_EF1a-SF-P2A-tdTom	AATTTATGCGTTTCAAAGTTCGAATGGAAGGGAGTATGAATGGACATGAGTTTGAGATAG	1380
350-10_c2	AATTTATGCGTTTCAAAGTTCGAATGGAAGGGAGTATGAATGGACATGAGTTTGAGATAG	1380
350-10_c3	AATTTATGCGTTTCAAAGTTCGAATGGAAGGGAGTATGAATGGACATGAGTTTGAGATAG	1380
350-10_c5	AATTTATGCGTTTCAAAGTTCGAATGGAAGGGAGTATGAATGGACATGAGTTTGAGATAG	1380
355-8_c11	AATTTATGCGTTTCAAAGTTCGAATGGAAGGGAGTATGAATGGACATGAGTTTGAGATAG	1380
355-8_c13	AATTTATGCGTTTCAAAGTTCGAATGGAAGGGAGTATGAATGGACATGAGTTTGAGATAG	1380
355-8_c14	AATTTATGCGTTTCAAAGTTCGAATGGAAGGGAGTATGAATGGACATGAGTTTGAGATAG	1380

```
                                       1,400                    1,420                    1,440
expected_sequence_EF1a-SF-P2A-tdTom  AAGGAGAAGGGGAAGGTAGACCCTATGAAGGCACTCAAACGGCTAAGTTAAAAGTTACAA 1440
350-10_c2                            AAGGAGAAGGGGAAGGTAGACCCTATGAAGGCACTCAAACGGCTAAGTTAAAAGTTACAA 1440
350-10_c3                            AAGGAGAAGGGGAAGGTAGACCCTATGAAGGCACTCAAACGGCTAAGTTAAAAGTTACAA 1440
350-10_c5                            AAGGAGAAGGGGAAGGTAGACCCTATGAAGGCACTCAAACGGCTAAGTTAAAAGTTACAA 1440
355-8_c11                            AAGGAGAAGGGGAAGGTAGACCCTATGAAGGCACTCAAACGGCTAAGTTAAAAGTTACAA 1440
355-8_c13                            AAGGAGAAGGGGAAGGTAGACCCTATGAAGGCACTCAAACGGCTAAGTTAAAAGTTACAA 1440
355-8_c14                            AAGGAGAAGGGGAAGGTAGACCCTATGAAGGCACTCAAACGGCTAAGTTAAAAGTTACAA 1440
                                       1,460                    1,480                    1,500
expected_sequence_EF1a-SF-P2A-tdTom  AGGGAGGTCCACTGCCATTTGCTTGGGATATTTTGTCTCCTCAATTCATGTATGGTTCGA 1500
350-10_c2                            AGGGAGGTCCACTGCCATTTGCTTGGGATATTTTGTCTCCTCAATTCATGTATGGTTCGA 1500
350-10_c3                            AGGGAGGTCCACTGCCATTTGCTTGGGATATTTTGTCTCCTCAATTCATGTATGGTTCGA 1500
350-10_c5                            AGGGAGGTCCACTGCCATTTGCTTGGGATATTTTGTCTCCTCAATTCATGTATGGTTCGA 1500
355-8_c11                            AGGGAGGTCCACTGCCATTTGCTTGGGATATTTTGTCTCCTCAATTCATGTATGGTTCGA 1500
355-8_c13                            AGGGAGGTCCACTGCCATTTGCTTGGGATATTTTGTCTCCTCAATTCATGTATGGTTCGA 1500
355-8_c14                            AGGGAGGTCCACTGCCATTTGCTTGGGATATTTTGTCTCCTCAATTCATGTATGGTTCGA 1500
                                       1,520                    1,540                    1,560
expected_sequence_EF1a-SF-P2A-tdTom  AAGCATACGTTAAACACCCTGCAGATATTCCGGACTATAAGAAGTTATCGTTTCCAGAAG 1560
350-10_c2                            AAGCATACGTTAAACACCCTGCAGATATTCCGGACTATAAGAAGTTATCGTTTCCAGAAG 1560
350-10_c3                            AAGCATACGTTAAACACCCTGCAGATATTCCGGACTATAAGAAGTTATCGTTTCCAGAAG 1560
350-10_c5                            AAGCATACGTTAAACACCCTGCAGATATTCCGGACTATAAGAAGTTATCGTTTCCAGAAG 1560
355-8_c11                            AAGCATACGTTAAACACCCTGCAGATATTCCGGACTATAAGAAGTTATCGTTTCCAGAAG 1560
355-8_c13                            AAGCATACGTTAAACACCCTGCAGATATTCCGGACTATAAGAAGTTATCGTTTCCAGAAG 1560
355-8_c14                            AAGCATACGTTAAACACCCTGCAGATATTCCGGACTATAAGAAGTTATCGTTTCCAGAAG 1560
                                       1,580                    1,600                    1,620
expected_sequence_EF1a-SF-P2A-tdTom  GTTTTAAATGGGAACGCGTTATGAACTTCGAAGATGGTGGGTTAGTAACAGTAACACAGG 1620
350-10_c2                            GTTTTAAATGGGAACGCGTTATGAACTTCGAAGATGGTGGGTTAGTAACAGTAACACAGG 1620
350-10_c3                            GTTTTAAATGGGAACGCGTTATGAACTTCGAAGATGGTGGGTTAGTAACAGTAACACAGG 1620
350-10_c5                            GTTTTAAATGGGAACGCGTTATGAACTTCGAAGATGGTGGGTTAGTAACAGTAACACAGG 1620
355-8_c11                            GTTTTAAATGGGAACGCGTTATGAACTTCGAAGATGGTGGGTTAGTAACAGTAACACAGG 1620
355-8_c13                            GTTTTAAATGGGAACGCGTTATGAACTTCGAAGATGGTGGGTTAGTAACAGTAACACAGG 1620
355-8_c14                            GTTTTAAATGGGAACGCGTTATGAACTTCGAAGATGGTGGGTTAGTAACAGTAACACAGG 1620
                                       1,640                    1,660                    1,680
expected_sequence_EF1a-SF-P2A-tdTom  ATAGCAGCTTGCAAGATGGTACGTTAATCTACAAAGTGAAAATGAGAGGCACCAATTTTC 1680
350-10_c2                            ATAGCAGCTTGCAAGATGGTACGTTAATCTACAAAGTGAAAATGAGAGGCACCAATTTTC 1680
350-10_c3                            ATAGCAGCTTGCAAGATGGTACGTTAATCTACAAAGTGAAAATGAGAGGCACCAATTTTC 1680
350-10_c5                            ATAGCAGCTTGCAAGATGGTACGTTAATCTACAAAGTGAAAATGAGAGGCACCAATTTTC 1680
355-8_c11                            ATAGCAGCTTGCAAGATGGTACGTTAATCTACAAAGTGAAAATGAGAGGCACCAATTTTC 1680
355-8_c13                            ATAGCAGCTTGCAAGATGGTACGTTAATCTACAAAGTGAAAATGAGAGGCACCAATTTTC 1680
355-8_c14                            ATAGCAGCTTGCAAGATGGTACGTTAATCTACAAAGTGAAAATGAGAGGCACCAATTTTC 1680
                                       1,700                    1,720                    1,740
expected_sequence_EF1a-SF-P2A-tdTom  CACCAGATGGACCAGTCATGCAGAAGAAAACTATGGGCTGGGAAGCAAGCACCGAAAGGC 1740
350-10_c2                            CACCAGATGGACCAGTCATGCAGAAGAAAACTATGGGCTGGGAAGCAAGCACCGAAAGGC 1740
350-10_c3                            CACCAGATGGACCAGTCATGCAGAAGAAAACTATGGGCTGGGAAGCAAGCACCGAAAGGC 1740
350-10_c5                            CACCAGATGGACCAGTCATGCAGAAGAAAACTATGGGCTGGGAAGCAAGCACCGAAAGGC 1740
355-8_c11                            CACCAGATGGACCAGTCATGCAGAAGAAAACTATGGGCTGGGAAGCAAGCACCGAAAGGC 1740
355-8_c13                            CACCAGATGGACCAGTCATGCAGAAGAAAACTATGGGCTGGGAAGCAAGCACCGAAAGGC 1740
355-8_c14                            CACCAGATGGACCAGTCATGCAGAAGAAAACTATGGGCTGGGAAGCAAGCACCGAAAGGC 1740
                                       1,760                    1,780                    1,800
expected_sequence_EF1a-SF-P2A-tdTom  TATATCCTAGGGATGGTGTTTTAAAAGGAGAGATCCATCAAGCTTTGAAACTAAAAGACG 1800
350-10_c2                            TATATCCTAGGGATGGTGTTTTAAAAGGAGAGATCCATCAAGCTTTGAAACTAAAAGACG 1800
350-10_c3                            TATATCCTAGGGATGGTGTTTTAAAAGGAGAGATCCATCAAGCTTTGAAACTAAAAGACG 1800
350-10_c5                            TATATCCTAGGGATGGTGTTTTAAAAGGAGAGATCCATCAAGCTTTGAAACTAAAAGACG 1800
355-8_c11                            TATATCCTAGGGATGGTGTTTTAAAAGGAGAGATCCATCAAGCTTTGAAACTAAAAGACG 1800
355-8_c13                            TATATCCTAGGGATGGTGTTTTAAAAGGAGAGATCCATCAAGCTTTGAAACTAAAAGACG 1800
355-8_c14                            TATATCCTAGGGATGGTGTTTTAAAAGGAGAGATCCATCAAGCTTTGAAACTAAAAGACG 1800
                                       1,820                    1,840                    1,860
expected_sequence_EF1a-SF-P2A-tdTom  GTGGCCACTATTTGGTGGAATTTAAGACCATATACATGGCTAAGAAACCGGTACAGTTAC 1860
350-10_c2                            GTGGCCACTATTTGGTGGAATTTAAGACCATATACATGGCTAAGAAACCGGTACAGTTAC 1860
350-10_c3                            GTGGCCACTATTTGGTGGAATTTAAGACCATATACATGGCTAAGAAACCGGTACAGTTAC 1860
350-10_c5                            GTGGCCACTATTTGGTGGAATTTAAGACCATATACATGGCTAAGAAACCGGTACAGTTAC 1860
355-8_c11                            GTGGCCACTATTTGGTGGAATTTAAGACCATATACATGGCTAAGAAACCGGTACAGTTAC 1860
355-8_c13                            GTGGCCACTATTTGGTGGAATTTAAGACCATATACATGGCTAAGAAACCGGTACAGTTAC 1860
355-8_c14                            GTGGCCACTATTTGGTGGAATTTAAGACCATATACATGGCTAAGAAACCGGTACAGTTAC 1860
```

```
                                                                   1,880                1,900                1,920
expected_sequence_EF1a-SF-P2A-tdTom CGGGATATTATTACGTGGATACTAAGCTAGATATAACATCTCACAACGAGGACTACACAA 1920
                         350-10_c2 CGGGATATTATTACGTGGATACTAAGCTAGATATAACATCTCACAACGAGGACTACACAA 1920
                         350-10_c3 CGGGATATTATTACGTGGATACTAAGCTAGATATAACATCTCACAACGAGGACTACACAA 1920
                         350-10_c5 CGGGATATTATTACGTGGATACTAAGCTAGATATAACATCTCACAACGAGGACTACACAA 1920
                         355-8_c11 CGGGATATTATTACGTGGATACTAAGCTAGATATAACATCTCACAACGAGGACTACACAA 1920
                         355-8_c13 CGGGATATTATTACGTGGATACTAAGCTAGATATAACATCTCACAACGAGGACTACACAA 1920
                         355-8_c14 CGGGATATTATTACGTGGATACTAAGCTAGATATAACATCTCACAACGAGGACTACACAA 1920
                                                                   1,940                1,960                1,980
expected_sequence_EF1a-SF-P2A-tdTom TTGTGGAACAATATGAAAGATCAGAAGGCCGTCATCATTTGTTTCTTGGCCATGGCACTG 1980
                         350-10_c2 TTGTGGAACAATATGAAAGATCAGAAGGCCGTCATCATTTGTTTCTTGGCCATGGCACTG 1980
                         350-10_c3 TTGTGGAACAATATGAAAGATCAGAAGGCCGTCATCATTTGTTTCTTGGCCATGGCACTG 1980
                         350-10_c5 TTGTGGAACAATATGAAAGATCAGAAGGCCGTCATCATTTGTTTCTTGGCCATGGCACTG 1980
                         355-8_c11 TTGTGGAACAATATGAAAGATCAGAAGGCCGTCATCATTTGTTTCTTGGCCATGGCACTG 1980
                         355-8_c13 TTGTGGAACAATATGAAAGATCAGAAGGCCGTCATCATTTGTTTCTTGGCCATGGCACTG 1980
                         355-8_c14 TTGTGGAACAATATGAAAGATCAGAAGGCCGTCATCATTTGTTTCTTGGCCATGGCACTG 1980
                                                                   2,000                2,020                2,040
expected_sequence_EF1a-SF-P2A-tdTom GTTCCACCGGTAGTGGATCATCAGGTACTGCTTCAAGTGAGGACAACAACATGGCGGTGA 2040
                         350-10_c2 GTTCCACCGGTAGTGGATCATCAGGTACTGCTTCAAGTGAGGACAACAACATGGCGGTGA 2040
                         350-10_c3 GTTCCACCGGTAGTGGATCATCAGGTACTGCTTCAAGTGAGGACAACAACATGGCGGTGA 2040
                         350-10_c5 GTTCCACCGGTAGTGGATCATCAGGTACTGCTTCAAGTGAGGACAACAACATGGCGGTGA 2040
                         355-8_c11 GTTCCACCGGTAGTGGATCATCAGGTACTGCTTCAAGTGAGGACAACAACATGGCGGTGA 2040
                         355-8_c13 GTTCCACCGGTAGTGGATCATCAGGTACTGCTTCAAGTGAGGACAACAACATGGCGGTGA 2040
                         355-8_c14 GTTCCACCGGTAGTGGATCATCAGGTACTGCTTCAAGTGAGGACAACAACATGGCGGTGA 2040
                                                                   2,060                2,080                2,100
expected_sequence_EF1a-SF-P2A-tdTom TAAAAGAGTTCATGAGATTTAAAGTGCGAATGGAGGGCTCAATGAATGGACACGAATTTG 2100
                         350-10_c2 TAAAAGAGTTCATGAGATTTAAAGTGCGAATGGAGGGCTCAATGAATGGACACGAATTTG 2100
                         350-10_c3 TAAAAGAGTTCATGAGATTTAAAGTGCGAATGGAGGGCTCAATGAATGGACACGAATTTG 2100
                         350-10_c5 TAAAAGAGTTCATGAGATTTAAAGTGCGAATGGAGGGCTCAATGAATGGACACGAATTTG 2100
                         355-8_c11 TAAAAGAGTTCATGAGATTTAAAGTGCGAATGGAGGGCTCAATGAATGGACACGAATTTG 2100
                         355-8_c13 TAAAAGAGTTCATGAGATTTAAAGTGCGAATGGAGGGCTCAATGAATGGACACGAATTTG 2100
                         355-8_c14 TAAAAGAGTTCATGAGATTTAAAGTGCGAATGGAGGGCTCAATGAATGGACACGAATTTG 2100
                                                                   2,120                2,140                2,160
expected_sequence_EF1a-SF-P2A-tdTom AAATTGAAGGGGAGGGTGAAGGACGACCGTACGAAGGAACACAAACAGCCAAATTGAAAG 2160
                         350-10_c2 AAATTGAAGGGGAGGGTGAAGGACGACCGTACGAAGGAACACAAACAGCCAAATTGAAAG 2160
                         350-10_c3 AAATTGAAGGGGAGGGTGAAGGACGACCGTACGAAGGAACACAAACAGCCAAATTGAAAG 2160
                         350-10_c5 AAATTGAAGGGGAGGGTGAAGGACGACCGTACGAAGGAACACAAACAGCCAAATTGAAAG 2160
                         355-8_c11 AAATTGAAGGGGAGGGTGAAGGACGACCGTACGAAGGAACACAAACAGCCAAATTGAAAG 2160
                         355-8_c13 AAATTGAAGGGGAGGGTGAAGGACGACCGTACGAAGGAACACAAACAGCCAAATTGAAAG 2160
                         355-8_c14 AAATTGAAGGGGAGGGTGAAGGACGACCGTACGAAGGAACACAAACAGCCAAATTGAAAG 2160
                                                                   2,180                2,200                2,220
expected_sequence_EF1a-SF-P2A-tdTom TAACTAAGGGAGGTCCTCTTCCTTTTGCATGGGACATCTTATCTCCACAATTTATGTACG 2220
                         350-10_c2 TAACTAAGGGAGGTCCTCTTCCTTTTGCATGGGACATCTTATCTCCACAATTTATGTACG 2220
                         350-10_c3 TAACTAAGGGAGGTCCTCTTCCTTTTGCATGGGACATCTTATCTCCACAATTTATGTACG 2220
                         350-10_c5 TAACTAAGGGAGGTCCTCTTCCTTTTGCATGGGACATCTTATCTCCACAATTTATGTACG 2220
                         355-8_c11 TAACTAAGGGAGGTCCTCTTCCTTTTGCATGGGACATCTTATCTCCACAATTTATGTACG 2220
                         355-8_c13 TAACTAAGGGAGGTCCTCTTCCTTTTGCATGGGACATCTTATCTCCACAATTTATGTACG 2220
                         355-8_c14 TAACTAAGGGAGGTCCTCTTCCTTTTGCATGGGACATCTTATCTCCACAATTTATGTACG 2220
                                                                   2,240                2,260                2,280
expected_sequence_EF1a-SF-P2A-tdTom GATCTAAAGCCTATGTCAAACATCCAGCTGATATTCCCGATTACAAGAAACTTAGCTTTC 2280
                         350-10_c2 GATCTAAAGCCTATGTCAAACATCCAGCTGATATTCCCGATTACAAGAAACTTAGCTTTC 2280
                         350-10_c3 GATCTAAAGCCTATGTCAAACATCCAGCTGATATTCCCGATTACAAGAAACTTAGCTTTC 2280
                         350-10_c5 GATCTAAAGCCTATGTCAAACATCCAGCTGATATTCCCGATTACAAGAAACTTAGCTTTC 2280
                         355-8_c11 GATCTAAAGCCTATGTCAAACATCCAGCTGATATTCCCGATTACAAGAAACTTAGCTTTC 2280
                         355-8_c13 GATCTAAAGCCTATGTCAAACATCCAGCTGATATTCCCGATTACAAGAAACTTAGCTTTC 2280
                         355-8_c14 GATCTAAAGCCTATGTCAAACATCCAGCTGATATTCCCGATTACAAGAAACTTAGCTTTC 2280
                                                                   2,300                2,320                2,340
expected_sequence_EF1a-SF-P2A-tdTom CAGAGGGATTCAAATGGGAACGTGTGATGAATTTCGAAGATGGAGGTTTAGTTACAGTAA 2340
                         350-10_c2 CAGAGGGATTCAAATGGGAACGTGTGATGAATTTCGAAGATGGAGGTTTAGTTACAGTAA 2340
                         350-10_c3 CAGAGGGATTCAAATGGGAACGTGTGATGAATTTCGAAGATGGAGGTTTAGTTACAGTAA 2340
                         350-10_c5 CAGAGGGATTCAAATGGGAACGTGTGATGAATTTCGAAGATGGAGGTTTAGTTACAGTAA 2340
                         355-8_c11 CAGAGGGATTCAAATGGGAACGTGTGATGAATTTCGAAGATGGAGGTTTAGTTACAGTAA 2340
                         355-8_c13 CAGAGGGATTCAAATGGGAACGTGTGATGAATTTCGAAGATGGAGGTTTAGTTACAGTAA 2340
                         355-8_c14 CAGAGGGATTCAAATGGGAACGTGTGATGAATTTCGAAGATGGAGGTTTAGTTACAGTAA 2340
```

Supporting Figure S7

```
                                                       2,360                    2,380                    2,400
expected_sequence_EF1a-SF-P2A-tdTom  CCCAAGACAGTTCTTTACAAGATGGAACTTTGATCTATAAAGTCAAAATGAGAGGAACAA 2400
                         350-10_c2  CCCAAGACAGTTCTTTACAAGATGGAACTTTGATCTATAAAGTCAAAATGAGAGGAACAA 2400
                         350-10_c3  CCCAAGACAGTTCTTTACAAGATGGAACTTTGATCTATAAAGTCAAAATGAGAGGAACAA 2400
                         350-10_c5  CCCAAGACAGTTCTTTACAAGATGGAACTTTGATCTATAAAGTCAAAATGAGAGGAACAA 2400
                         355-8_c11  CCCAAGACAGTTCTTTACAAGATGGAACTTTGATCTATAAAGTCAAAATGAGAGGAACAA 2400
                         355-8_c13  CCCAAGACAGTTCTTTACAAGATGGAACTTTGATCTATAAAGTCAAAATGAGAGGAACAA 2400
                         355-8_c14  CCCAAGACAGTTCTTTACAAGATGGAACTTTGATCTATAAAGTCAAAATGAGAGGAACAA 2400

                                                                                  Pr899
                                                       2,420                    2,440
expected_sequence_EF1a-SF-P2A-tdTom  ATTTCCCACCAGATGGGCCTGTTATGCAAAAGAAAACGATGGGTTGGGAGGCATCAA 2457
                         350-10_c2  ATTTCCCACCAGATGGGCCTGTTATGCAAAAGAAAACGATGGGTTGGGAGGCATCAA 2457
                         350-10_c3  ATTTCCCACCAGATGGGCCTGTTATGCAAAAGAAAACGATGGGTTGGGAGGCATCAA 2457
                         350-10_c5  ATTTCCCACCAGATGGGCCTGTTATGCAAAAGAAAACGATGGGTTGGGAGGCATCAA 2457
                         355-8_c11  ATTTCCCACCAGATGGGCCTGTTATGCAAAAGAAAACGATGGGTTGGGAGGCATCAA 2457
                         355-8_c13  ATTTCCCACCAGATGGGCCTGTTATGCAAAAGAAAACGATGGGTTGGGAGGCATCAA 2457
                         355-8_c14  ATTTCCCACCAGATGGGCCTGTTATGCAAAAGAAAACGATGGGTTGGGAGGCATCAA 2457
```

Fig S6. Alignment of upstream and downstream integration sites from *Eef1a*$^{SF\text{-}P2A\text{-}tdTom}$ colonies.

Nucleotide alignment of the region encoding the upstream integration site from the founder colony, 350-10, and one transgenic offspring, 355-8. Three cloned amplicons were sequenced for each animal at each site. Primer binding sites are indicated in green on the expected sequence.
